# *Tcf7l2* in hepatocytes regulates de novo lipogenesis in diet-induced non-alcoholic fatty liver disease in mice

**DOI:** 10.1007/s00125-023-05878-8

**Published:** 2023-02-10

**Authors:** Da Som Lee, Tae Hyeon An, Hyunmi Kim, Eunsun Jung, Gyeonghun Kim, Seung Yeon Oh, Jun Seok Kim, Hye Jin Chun, Jaeeun Jung, Eun-Woo Lee, Baek-Soo Han, Dai Hoon Han, Yong-Ho Lee, Tae-Su Han, Keun Hur, Chul-Ho Lee, Dae-Soo Kim, Won Kon Kim, Jun Won Park, Seung-Hoi Koo, Je Kyung Seong, Sang Chul Lee, Hail Kim, Kwang-Hee Bae, Kyoung-Jin Oh

**Affiliations:** 1 Graduate School of Medical Science and Engineering, Korea Advanced Institute of Science and Technology, Daejeon, Republic of Korea; 2 Metabolic Regulation Research Center, Korea Research Institute of Bioscience and Biotechnology (KRIBB), Daejeon, Republic of Korea; 3 Department of Functional Genomics, KRIBB School of Bioscience, University of Science and Technology (UST), Daejeon, Republic of Korea; 4 Biotherapeutics Translational Research Center, Korea Research Institute of Bioscience and Biotechnology (KRIBB), Daejeon, Republic of Korea; 5 College of Veterinary Medicine, Seoul National University, Seoul, Republic of Korea; 6 Korea Mouse Phenotyping Center (KMPC), Seoul National University, Seoul, Republic of Korea; 7 Division of Life Sciences, Korea University, Seoul, Republic of Korea; 8 Department of Systems Biology, Glycosylation Network Research Center, Yonsei University, Seoul, Republic of Korea; 9 Environmental Diseases Research Center, Korea Research Institute of Bioscience and Biotechnology (KRIBB), Daejeon, Republic of Korea; 10 Biodefense Research Center, Korea Research Institute of Bioscience and Biotechnology (KRIBB), Daejeon, Republic of Korea; 11 Department of Surgery, Yonsei University College of Medicine, Seoul, Republic of Korea; 12 Department of Internal Medicine, Yonsei University College of Medicine, Seoul, Republic of Korea; 13 Department of Biochemistry and Cell Biology, School of Medicine, Kyungpook National University, Daegu, Republic of Korea; 14 Laboratory Animal Resource Center, Korea Research Institute of Bioscience and Biotechnology (KRIBB), Daejeon, Republic of Korea; 15 Division of Biomedical Convergence, College of Biomedical Science, Kangwon National University, ChunCheon-si, Gangwon-do Republic of Korea

**Keywords:** Carbohydrate, De novo lipogenesis, Fatty acid, Glucose, Insulin, Lipogenesis, NAFLD, TCF7L2, TG accumulation, Type 2 diabetes

## Abstract

**Aims/hypothesis:**

Non-alcoholic fatty liver disease (NAFLD) associated with type 2 diabetes may more easily progress towards severe forms of non-alcoholic steatohepatitis (NASH) and cirrhosis. Although the Wnt effector transcription factor 7-like 2 (TCF7L2) is closely associated with type 2 diabetes risk, the role of TCF7L2 in NAFLD development remains unclear. Here, we investigated how changes in TCF7L2 expression in the liver affects hepatic lipid metabolism based on the major risk factors of NAFLD development.

**Methods:**

*Tcf7l2* was selectively ablated in the liver of C57BL/6N mice by inducing the albumin (*Alb*) promoter to recombine *Tcf7l2* alleles floxed at exon 5 (liver-specific *Tcf7l2*-knockout [KO] mice: *Alb-Cre;Tcf7l2*^*f/f*^). *Alb-Cre;Tcf7l2*^*f/f*^ and their wild-type (*Tcf7l2*^*f/f*^) littermates were fed a high-fat diet (HFD) or a high-carbohydrate diet (HCD) for 22 weeks to reproduce NAFLD/NASH. Mice were refed a standard chow diet or an HCD to stimulate de novo lipogenesis (DNL) or fed an HFD to provide exogenous fatty acids. We analysed glucose and insulin sensitivity, metabolic respiration, mRNA expression profiles, hepatic triglyceride (TG), hepatic DNL, selected hepatic metabolites, selected plasma metabolites and liver histology.

**Results:**

*Alb-Cre;Tcf7l2*^*f/f*^ essentially exhibited increased lipogenic genes, but there were no changes in hepatic lipid content in mice fed a normal chow diet. However, following 22 weeks of diet-induced NAFLD/NASH conditions, liver steatosis was exacerbated owing to preferential metabolism of carbohydrate over fat. Indeed, hepatic *Tcf7l2* deficiency enhanced liver lipid content in a manner that was dependent on the duration and amount of exposure to carbohydrates, owing to cell-autonomous increases in hepatic DNL. Mechanistically, TCF7L2 regulated the transcriptional activity of *Mlxipl* (also known as *ChREBP*) by modulating *O*-GlcNAcylation and protein content of carbohydrate response element binding protein (ChREBP), and targeted *Srebf1* (also called *SREBP1*) via miRNA (miR)-33-5p in hepatocytes. Eventually, restoring *TCF7L2* expression at the physiological level in the liver of *Alb-Cre;Tcf7l2*^*f/f*^ mice alleviated liver steatosis without altering body composition under both acute and chronic HCD conditions.

**Conclusions/interpretation:**

In mice, loss of hepatic *Tcf7l2* contributes to liver steatosis by inducing preferential metabolism of carbohydrates via DNL activation. Therefore, TCF7L2 could be a promising regulator of the NAFLD associated with high-carbohydrate diets and diabetes since TCF7L2 deficiency may lead to development of NAFLD by promoting utilisation of excess glucose pools through activating DNL.

**Data availability:**

RNA-sequencing data have been deposited into the NCBI GEO under the accession number GSE162449 (www.ncbi.nlm.nih.gov/geo/query/acc.cgi?acc=GSE162449).

**Graphical abstract:**

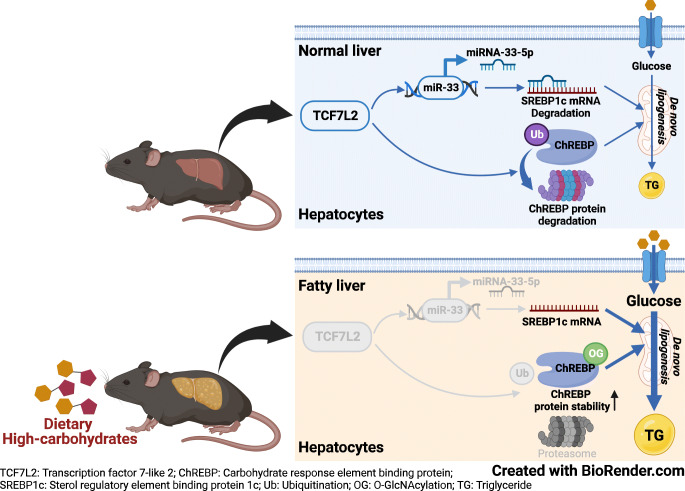

**Supplementary Information:**

The online version of this article (10.1007/s00125-023-05878-8) contains peer-reviewed but unedited supplementary material..

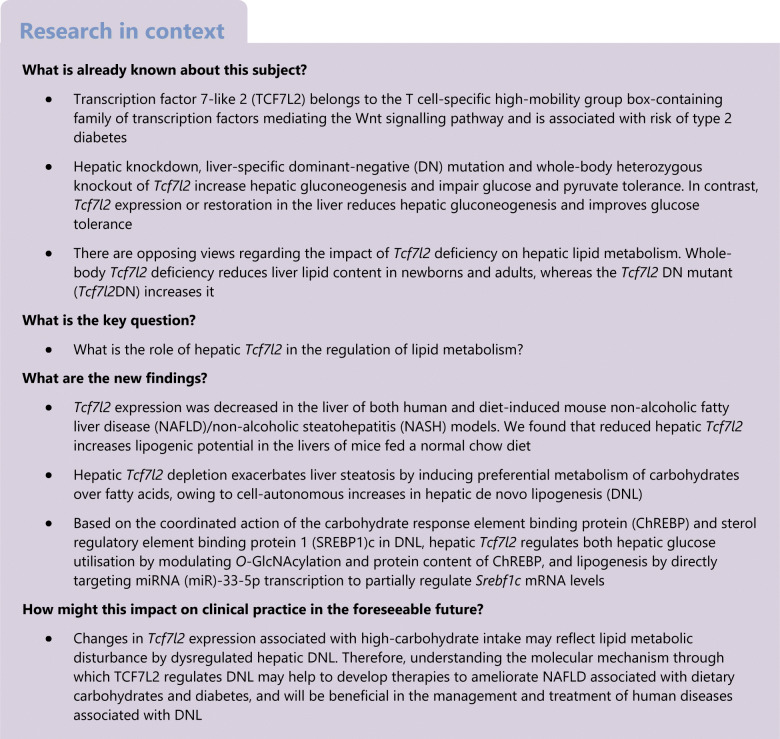



## Introduction

Transcription factor 7-like 2 (TCF7L2, also known as transcription factor 4 [TCF4]) belongs to the T cell-specific high-mobility group box-containing family of transcription factors that mediates the canonical Wnt signalling pathway [1, 2]. It is well known for its important role in cell development and regeneration [1, 2]. It has also been described as a critical transcription factor in maintaining glucose homeostasis [1–8]. In mice, reduced TCF7L2 expression is associated with impaired glucose metabolism and defective insulin signalling in the pancreatic beta cells [3] and adipose tissue [4, 5]. Loss of hepatic *Tcf7l2* disturbs hepatic glucose metabolism by promoting hepatic gluconeogenesis, resulting in impaired insulin signalling pathways [6–8].

According to genome-wide association studies in humans, *Tcf7l2* is one of the strongest risk factors associated with type 2 diabetes [9, 10]. However, it remains unclear whether the genetic alteration of *Tcf7l2* affects its expression. Therefore, *Tcf7l2*-deficient in vitro and in vivo models are being used to track the association between SNPs in *Tcf7l2* and *Tcf7l2* expression, and to gain insights into diabetes-related molecular pathways [1, 11]. Many studies have shown that alterations in *Tcf7l2* expression are metabolically associated with type 2 diabetes [1, 11].

*Tcf7l2* has also been proposed as a genetic factor that can explain the association between type 2 diabetes and non-alcoholic fatty liver disease (NAFLD), as genetic alterations in *Tcf7l2* can make individuals susceptible to NAFLD [12]. Recently, the coexistence of NAFLD and type 2 diabetes has emerged as an important issue because individuals with NAFLD plus type 2 diabetes have a poor metabolic profile and a higher risk of worsening with more advanced forms of disease, such as non-alcoholic steatohepatitis (NASH), cirrhosis and hepatocellular carcinoma (HCC) [13–15]. However, the association of TCF7L2 with NAFLD remains unclear and little is known about the underlying mechanisms and function of TCF7L2 in the pathogenesis of NAFLD.

NAFLD is characterised by excessive hepatic triglyceride (TG) accumulation, which is primarily caused by excessive exogenous fatty acid uptake and increased endogenous fatty acid synthesis (de novo lipogenesis [DNL]) in the liver [16–19]. In this study, to determine the role of hepatic *Tcf7l2* deficiency in NAFLD, we generated liver-specific *Tcf7l2* knockout (KO) mice (*Alb-Cre;Tcf7l2*^*f/f*^). Based on the major risk factors for NAFLD development [18], mice lacking hepatic *Tcf7l2* were given a high-fat diet (HFD) to provide fatty acids, or a refeeding/high-carbohydrate diet (HCD) to stimulate DNL. We thoroughly explored the underlying mechanisms and metabolic phenotype of liver TG accumulation owing to hepatic *Tcf7l2* deficiency in in vivo and in vitro systems.

## Methods

For detailed methods, please refer to the electronic supplementary material (ESM).

### Human liver tissue samples

Human liver tissue samples in ESM Fig. 1a were collected from 32 individuals who underwent hepatectomy or cholecystectomy at the university-affiliated Severance Hospital, Yonsei University College of Medicine, Republic of Korea, between September 2014 and October 2019, as previously described [20]. Liver histology was assessed by an experienced pathologist and, according to diagnostic criteria [21], the samples were put into the following groups: (1) normal (*n*=13); (2) simple steatosis (NAFLD; *n*=10); and (3) NASH (*n*=9). All individuals gave informed consent and the study protocol was approved by the Institutional Review Board at Severance Hospital (IRB No 4–2014–0674).

### Animal experiments

Seven-week-old male C57BL/6N mice were purchased from ORIENT BIO (ORIENT BIO, Republic of Korea). Hepatocyte-selective *Tcf7l2* ablation in C57BL/6N mice (liver-specific *Tcf7l2* KO mice: *Alb-Cre;Tcf7l2*^*f/f*^) was obtained by crossing mice carrying *Cre* recombinase driven by the albumin (*Alb*) promoter with mice carrying *Tcf7l2* alleles floxed at exon 5. Six-week-old male *Alb-Cre;Tcf7l2*^*f/f*^ and their wild-type littermates (*Tcf7l2*^*f/f*^ mice; developed in-house from *Tcf7l2*^*+/−*^ mice [*Tcf7l2*^tm2a(EUCOMM)Wtsi^; the EUCOMM Consortium; www.mousephenotype.org/data/genes/MGI:1202879, accessed 8 September 2022]) were given either an HFD (catalogue no. D12492; Research Diet, USA) or HCD (catalogue no. TD.98090; Envigo, USA) for the indicated periods. For GTTs and ITTs, mice were fasted for 16 h or 6 h, respectively, and injected intraperitoneally with 1.5 g/kg of glucose or 1 U/kg of insulin. Blood glucose levels were measured in samples taken from the tail vein (LifeScan; One-Touch, USA). $\dot{V}{\mathrm{O}}_2$, carbon dioxide production ($\dot{V}\mathrm{C}{\mathrm{O}}_2$), respiratory exchange ratio (RER; $\dot{V}\mathrm{C}{\mathrm{O}}_2$/$\dot{V}{\mathrm{O}}_2$) and energy expenditure were measured in mice at 10 weeks using the Oxylet Pro system (Panlab, Spain). All animal procedures were approved by the Institutional Animal Care and Use Committee of the Korea Research Institute of Bioscience & Biotechnology (KRIBB-AEC-21051) and were performed in accordance with the guidelines for the Care and Use of Laboratory Animals published by the US National Institutes of Health.

### Mouse primary hepatocyte culture

Primary hepatocytes were isolated from 8-week-old male C57BL/6N mice by collagenase perfusion [7]. The cells were maintained in Medium 199 (catalogue no. M4530; Sigma-Aldrich, UK) supplemented with 10% (vol./vol.) FBS, 1% (vol./vol.) antibiotics and 10 nmol/l dexamethasone, and were infected with adenovirus for 24 h. Subsequently, the cells were maintained in medium 199 without 10% FBS and were treated with 25 mmol/l glucose (catalogue no. G7021; Sigma-Aldrich) and/or 100 nmol/l insulin (catalogue no. I6634; Sigma-Aldrich) for 48 h, 250 μmol/l palmitic acid (catalogue no. P9767; Sigma-Aldrich) for 24 h, 300 μmol/l AICAR (catalogue no. A611700; Toronto Research Chemicals, Canada) for 1 h, or 1 μmol/l T0901317 (catalogue no. T2320; Sigma-Aldrich) for 24 h.

### Quantitative PCR

Total RNA from human liver tissues, mouse tissues or mouse primary hepatocytes was extracted using the easy-spin Total RNA Extraction Kit (catalogue no. 17221; iNtRON Biotechnology, Republic of Korea). Quantitative PCR (qPCR) was performed using the SYBR green PCR kit in a C1000 Touch Thermal Cycler (Bio-Rad Laboratories, USA). All data were normalised to the expression of ribosomal *L32*. Primer sequences are listed in ESM Table 1. miRNA (miR) expression was analysed as previously described [22] and the miR primer sequences are listed in ESM Table 2.

### Western blotting

Western blot analysis of extracts from mouse tissues and whole cells was performed as previously described [23]. Primary and secondary antibodies were used according to manufacturer’s instructions and are listed in ESM Table 3.

### Metabolite measurements

Plasma insulin and IGF-1 levels were measured with a Mouse Insulin ELISA Kit (catalogue no. 80-INSMSE01; ALPCO, USA) and Mouse IGF1 ELISA Kit (catalogue no. EMIGF1; Thermo Fisher Scientific), respectively. Total lipids from mouse livers or mouse primary hepatocytes were extracted using the Folch method as described previously [7]. Hepatic TG, cellular TG, plasma TG and NEFA levels were measured with colorimetric assay kits (TG: catalogue no. 461-09092; NEFA: catalogue no. 438-91691; Wako, Japan). Hepatic glycogen was measured using an EnzyChrom Glycogen Assay Kit (catalogue no. E2GN-100; BioAssay Systems, USA). Hepatic β-hydroxybutyrate (β-OH) was measured with a fluorometric assay kit (catalogue no. 700740; Cayman Chemical, USA).

### Immunofluorescence staining

Mouse pancreas tissues in ESM Fig. 1e and ESM Fig. 2d were fixed with 4% (wt/vol.) paraformaldehyde and were embedded in paraffin. Sliced specimens were immunostained using anti-insulin (catalogue no. 53-9769-82; Thermo Fisher Scientific) and anti-glucagon (catalogue no. ab92517; Abcam, USA) antibodies with DAPI (catalogue no. H-1800; Vector Laboratories, USA). The fluorescence images were taken using an OLYMPUS IX73 microscope (Olympus, Japan). Insulin-positive areas (beta cells), glucagon-positive areas (alpha cells) and pancreatic areas were quantified using Image J software (v 1.50i; https://imagej.nih.gov/ij/download.html). Antibody information is listed in ESM Table 4.

### Histological analysis

Liver tissue sections were stained with Oil Red O (catalogue no. O0625; Sigma-Aldrich) and H&E, as previously described [24]. Liver fibrosis was detected with Sirius red staining (catalogue no. ab150681; Abcam, USA) according to the manufacturer’s instructions. NAFLD Activity Score (NAS) and fibrosis stage were calculated as the sum of the scores for steatosis (0–3), lobular inflammation (0–3), hepatocyte ballooning (0–2), as assessed by H&E staining, and fibrosis (0–4), assessed by Sirius red staining [21, 25]. Histological samples were anonymised and assigned a number before the slides were analysed by an experienced pathologist.

### Fatty acid uptake assay

Mouse primary hepatocytes isolated from *Tcf7l2*^*f/f*^ or C57BL/6N mice were transfected with the indicated plasmid DNA vectors using Lipofectamine 3000 transfection reagent (catalogue no. L3000015; Invitrogen, USA). Fatty acid uptake in mouse primary hepatocytes was measured with the Fatty Acid Uptake Assay Kit (catalogue no. K408-100; Biovision, USA) according to the manufacturer’s instructions. Fluorescent fatty acid probes (green) were detected by a fluorescence microscope (DM IL LED FLUO; Leica Microsystems, Germany) and fluorescence intensity was quantified using Image J software (v 1.50i).

### Glucose uptake assay

Mouse primary hepatocytes isolated from *Tcf7l2*^*f/f*^ mice were infected with adenoviruses expressing *gfp* only and *Cre* and treated with 1 nmol/l insulin for 30 min. 2-Deoxyglucose (2-DG) levels in mouse primary hepatocytes were measured using the 2-DG Uptake Measurement Kit (catalogue no. CSR-OKP-PMG-K01; Cosmo Bio, Japan) according to the manufacturer’s instructions.

### Analysis of oxygen consumption rate via Seahorse assay

Fatty acid oxidation in mouse primary hepatocytes was assessed by analysing oxygen consumption rate (OCR) with an XF24 extracellular flux analyser (Seahorse Bioscience, USA). Mouse primary hepatocytes were seeded on collagen-coated XF24 cell culture microplates. Cells were treated with BSA or 250 μmol/l palmitate and/or 100 μmol/l etomoxir (catalogue no. E1905; Sigma-Aldrich), and then stimulated with 2.5 μmol/l oligomycin (catalogue no. O4876; Sigma-Aldrich), 10 μmol/l fluoro-carbonylcyanide phenylhydrazone (FCCP; catalogue no. C2920; Sigma-Aldrich), and 2 μmol/l rotenone (catalogue no. R8875; Sigma-Aldrich) plus 5 μmol/l antimycin A (catalogue no. A8674; Sigma-Aldrich), according to the manufacturer’s instructions. OCR levels were normalised to the amount of protein in each sample.

### DNL measurements

For in vitro DNL analysis, mouse primary hepatocytes were treated with 37 kBq of ^14^C-labelled glucose (catalogue no. NEC042; PerkinElmer, USA) and 10 nmol/l insulin for 48 h. Lipids were extracted from cells with chloroform and ^14^C radioactivity was measured using the Tri-Carb 2910 TR liquid scintillation analyser (PerkinElmer). For in vivo DNL analysis, mice were fasted for 24 h, then refed an HCD for 12 h. Subsequently, they were intraperitoneally injected with ^14^C-labelled sodium acetate (555 kBq/mouse; catalogue no. NEC553; PerkinElmer), or 3% (vol./vol.) ethanol in saline (136.9 mmol/l NaCl) as a control, and euthanised 1 h post injection. Lipids were extracted using the Folch method [7] and incorporation rates of [1-^14^C]acetic acid into lipids were measured using the Tri-Carb 2910 TR liquid scintillation analyser.

### mRNA sequencing

Total RNA from mouse liver was extracted using the easy-spin Total RNA Extraction Kit (iNtRON Biotechnology). The library preparation, mRNA sequencing and analysis were performed by eBiogen (Republic of Korea). Differentially expressed genes were determined based on counts from unique and multiple alignments using coverage in Bedtools (v 2.25.0; https://bedtools.readthedocs.io/en/latest/index.html). The read count (RC) data were processed based on the quantile normalisation method using EdgeR (v 3.20.1; https://bioconductor.org/packages/release/bioc/html/edgeR.html) within R (v 3.4.4; www.r-project.org) using Bioconductor (v 3.6; https://bioconductor.org/install/). From the results obtained by setting the RC (log_2_) value to 5 or more, genes with a log_2_ fold change greater than 1.5 (log_2_ fold-change cut-off >1.5) in KO vs wild-type comparisons were selected and analysed using Gene Set Enrichment Analysis (GSEA; www.gsea-msigdb.org/gsea/msigdb/mouse/annotate.jsp, accessed 1 November 2021), based on canonical pathways gene sets derived from the Reactome pathway database (www.gsea-msigdb.org/gsea/msigdb/mouse/genesets.jsp?collection=CP:REACTOME, accessed 1 November 2021).

### Plasmids and recombinant adenoviruses

Expression vectors for mouse *Tcf7l2*, nuclear *Srebf1c* and *Mlxipl(α)* were amplified by RT-PCR using liver RNA derived from C57BL/6N mice and inserted into pcDNA3-Flag or pcDNA3-HA expression vectors. The pGL4-6X *SRE*-luc (containing six copies of *SRE*) and pGL4-4X *ChoRE*-luc (containing four copies of *ChoRE*) constructs were a kind gift from S.-H. Koo (Korea University, Korea). Mouse *Fasn* (−2000/+200), *Pklr* (−191/+200), *Srebf1c* (−1195/+77), miR-33-5p (−852/+16), miR-33-5p (−1412/+16), *Mlxipl(α)* (−2538/+124) and *Mlxipl(β)* (−536/+352) promoter sequences were amplified by PCR using mouse genomic DNA and inserted into the pGL4-luciferase reporter vector. miRNA mimics (mmu-miR-33-5p, mmu-miR-132-3p, mmu-miR-212-3p and negative control) were purchased from GenePharma (Shanghai, China). Primer sequences are listed in ESM Table 5. In total, 50 μmol/l miRNA mimics were transfected into cells using Lipofectamine 3000 transfection reagent (Invitrogen), according to the manufacturer’s instructions. Adenoviruses expressing *gfp* only, *Tcf7l2* and *Cre* have been described previously [7]. For animal experiments, the viruses were purified on a CsCl gradient, dialysed against PBS buffer containing 10% (vol./vol.) glycerol and stored at −80°C.

### Luciferase assay

Luciferase assays were performed to determine the effects of TCF7L2 on the sterol regulatory element binding protein 1 (SREBP1)c, ChREBP/max-like protein X (MLX) transcriptional activities and the miR-33-5p promoter activity in HEPG2 cells (catalogue no. HB-8065; ATCC, USA) and *Srebf1* 3′UTR promoter activity in HEK293T cells (catalogue no. CRL-3216, ATCC). HEPG2 and HEK293T cells were tested for mycoplasma using the BioMycoX Mycoplasma PCR Detection Kit (catalogue no. D-50; CellSafe, Republic of Korea) and were found to be mycoplasma negative. Promoter activity was measured using a luciferase reporter assay system (catalogue no. E1910; Promega, USA) and normalised to β-galactosidase activity.

### Protein stability assay

HEPG2 cells were transfected with HA-tagged TCF7L2 and Flag-tagged ChREBPα for 48 h and treated with 10 μmol/l MG132 (catalogue no. M1157; AG Scientific, USA) or DMSO (catalogue no. D2650; Sigma-Aldrich) for 3 h.

### Immunoprecipitation and wheat germ agglutinin purification

Total lysates from liver tissues were centrifuged at 16,000 × *g* for 10 min and the supernatant was extracted. For immunoprecipitation, 3 mg of protein lysates were incubated with carbohydrate response element binding protein (ChREBP) antibody and 30 μl of protein G plus A agarose beads (catalogue no. IP05; Millipore, USA) was added to each sample. For wheat germ agglutinin (WGA) precipitation, 3 mg of protein lysates were incubated with 30 μl of WGA agarose beads (catalogue no. AL-1023; Vector Laboratories), after which beads were eluted in 2× sample buffer without β-mercaptoethanol.

### *Tcf7l2*-KO in alpha-mouse-liver-12 cell lines

To generate *Tcf7l2*-KO alpha-mouse-liver-12 (AML12) cell lines (catalogue no. CRL-2254; ATCC), we developed a CRISPR/Cas9 system for gene editing using standard methods [26]. The single-guide RNA (sgRNA) sequence was cloned into pSpCas9(BB)-2A-Puro(PX459), which was a gift from F. Zhang (Broad Institute of MIT and Harvard, Cambridge, USA). The target guide RNA (gRNA) sequence was 5′-AGCAATGAACACTTCACCCC-3′ (*Tcf7l2* exon 5; Genome Reference Consortium Mouse Reference 39 [GRCm39;]: 19:55,896,921–55,896,940; https://asia.ensembl.org/Mus_musculus/Info/Annotation). The vector expressing gRNA was transfected into AML12 cells and then cells were selected using puromycin. Cells tested negative for mycoplasma contamination. Genomic DNA was extracted from cells using the Exgene Tissue SV Kit (catalogue no. 104-152; GeneAll, Republic of Korea) Gene knockout was verified by PCR and western blot analysis. Cells were transfected with the pGL4-*Pklr* (−191/+200) promoter and then co-treated with 25 mmol/l glucose and 40 μmol/l OSMI-1 (catalogue no. ab235455; Abcam, UK) for 24 h.

### Chromatin immunoprecipitation

Cross-linking, nuclear isolation and chromatin immunoprecipitation (ChIP) assays were performed on AML12 cell line samples and mouse primary hepatocyte samples, using previously reported methods [27]. The precipitated DNA fragments were analysed by PCR.

### Statistical analysis

Statistical differences between two experimental groups were evaluated by the two-tailed unpaired Student’s *t* test. One-way ANOVA with Tukey’s multiple comparison test was performed using GraphPad Prism 8.0.1 (GraphPad Software, USA) when comparing three or more groups, as reported in the figure legends. Data are shown as mean±SD or mean±SEM. A *p* value <0.05 was considered statistically significant.

## Results

### Loss of hepatic *TCF7L2* exerts lipogenic potential

**Fig. 1:**
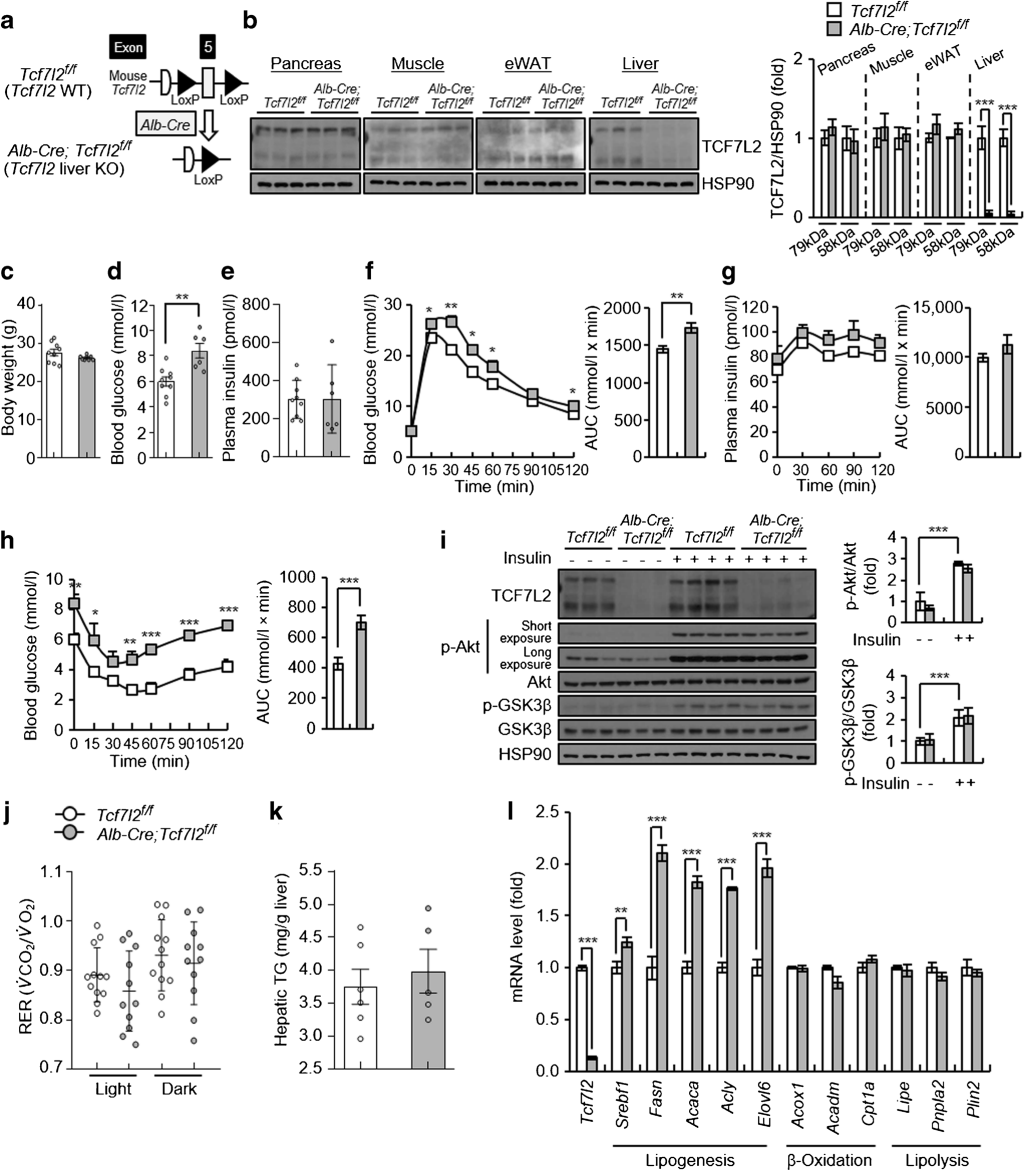
Metabolic phenotypes of *Alb-Cre;Tcf7l2*^*f/f*^ mice on a normal chow diet (NCD). (**a**) Strategy for generating *Alb-Cre;Tcf7l2*^*f/f*^ (mice with *Tcf7l2* liver-specific KO). (**b**) Representative western blot showing TCF7L2 expression in the peripheral tissues of 10-week-old *Tcf7l2*^*f/f*^ (*n*=3) and *Alb-Cre;Tcf7l2*^*f/f*^ (*n*=3) mice. Quantification of TCF7L2 protein levels is also shown. eWAT, epididymal white adipose tissue; WT, wild-type. (**c–e**) 10-week-old *Tcf7l2*^*f/f*^ (*n*=9) and *Alb-Cre;Tcf7l2*^*f/f*^ (*n*=6) mice fed an NCD were assessed for body weight (**c**), blood glucose levels (**d**) and plasma insulin levels (**e**). (**f**) GTT (1.5 g/kg body weight) in 10-week-old *Tcf7l2*^*f/f*^ (*n*=9) and *Alb-Cre;Tcf7l2*^*f/f*^ (*n*=6) mice fed an NCD, with glucose AUC is presented. *p<0.05, **p<0.01, *Alb-Cre;Tcf7l2*^*f/f*^ vs wild-type mice at the same time point, analysed by unpaired Student’s *t* test. (**g**) Plasma insulin concentration during GTT in 10-week-old *Tcf7l2*^*f/f*^ (*n*=9) and *Alb-Cre;Tcf7l2*^*f/f*^ (*n*=6) mice fed an NCD, with insulin AUC presented. (**h**) ITT (1 U/kg body weight) in 10-week-old *Tcf7l2*^*f/f*^ (*n*=9) and *Alb-Cre;Tcf7l2*^*f/f*^ (*n*=6) mice fed an NCD, with glucose AUC is presented. *p<0.05, **p<0.01, ***p<0.001, *Alb-Cre;Tcf7l2*^*f/f*^ vs wild-type mice at the same time point, analysed by unpaired Student’s *t* test. (**i**) Ten-week-old *Tcf7l2*^*f/f*^ (*n*=12) and *Alb-Cre;Tcf7l2*^*f/f*^ (*n*=12) mice were fasted for 6 h and then injected intraperitoneally with insulin (10 U/kg body weight) or saline for 10 min. Representative western blot showing the effects of hepatic *Tcf7l2* depletion on the hepatic insulin signalling pathway. The p-Akt/Akt and phosphorylated glycogen synthase kinase-3 β (p-GSK3β/GSK3β ratios are presented. (**j**) RER in 10-week-old *Tcf7l2*^*f/f*^ (*n*=12) and *Alb-Cre;Tcf7l2*^*f/f*^ (*n*=11) mice fed an NCD. RER was measured hourly in each metabolic chamber using the Oxylet Pro system. Average RER values for light (09:00–21:00 hours) and dark (21:00–09:00 hours) phases in a 12 h/12 h light/dark cycle are presented. (**k**, **l**) Ten-week-old *Tcf7l2*^*f/f*^ (*n*=6) and *Alb-Cre;Tcf7l2*^*f/f*^ (*n*=5) mice were fasted for 6 h, after which hepatic TG levels (**k**) and gene expression (**l**) were analysed. (**l**) Quantitative PCR (qPCR) analysis showing mRNA expression of genes involved in lipid metabolism in the liver of mice. The key in (**b**) also applies to (**c**–**i**) (**k**) and (**l**). Data in (**b**), (**i**) and (**l**) are presented as mean±SD; data in (**c–h**) (**j**) and (**k**) are presented as mean±SEM. *p<0.05, **p<0.01, ***p<0.001, analysed by *t* test

We found that *Tcf7l2* expression was significantly reduced in the liver of individuals with NAFLD and NASH (ESM Fig. 1a). Therefore, to gain new insights into the role of TCF7L2 in NAFLD development, we generated liver-specific *Tcf7l2* KO mice (*Alb-Cre;Tcf7l2*^*f/f*^; Fig. 1a,b and ESM Fig. 1b). Essentially, *Alb-Cre;Tcf7l2*^*f/f*^ mice exhibited increased fasting glucose levels without changes in body weight or plasma insulin and IGF-1 levels (Fig. 1c–e and ESM Fig. 1c). They also displayed impaired glucose and insulin tolerance (Fig. 1f,h and ESM Fig. 1d). However, there were no changes in insulin concentration patterns during GTT (Fig. 1g) or in islet glucagon-positive alpha cell and insulin-positive beta cell areas (ESM Fig. 1e,f). Additionally, hepatic *Tcf7l2* deficiency did not lead to changes in hepatic insulin signalling, RER, $\dot{V}{\mathrm{O}}_2$, $\dot{V}\mathrm{C}{\mathrm{O}}_2$ and energy expenditure, as compared with wild-type controls (Fig. 1i,j and ESM Fig. 1g). Although there were no significant changes in liver TG content (Fig. 1k), hepatic *Tcf7l2* depletion significantly enhanced hepatic expression of lipogenic genes, without altering genes involved in β-oxidation and lipolysis (Fig. 1l). Additionally, there were no changes in *Igf1* and *Igf1R* mRNA expression (ESM Fig. 1h).

### Hepatic *Tcf7l2* deficiency contributes to NAFLD development by inducing preferential metabolism of carbohydrates

**Fig. 2:**
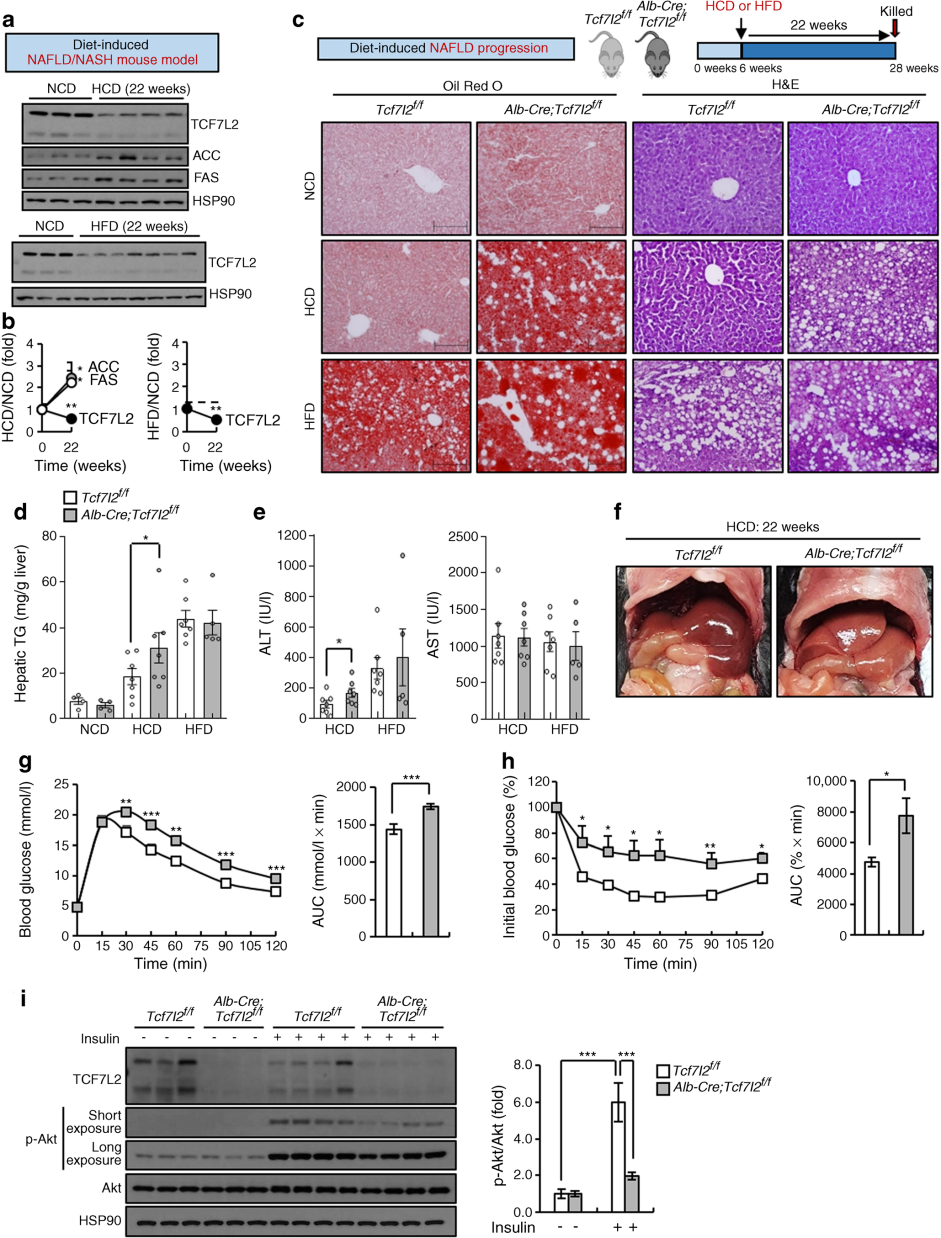
Effects of hepatic *Tcf7l2* depletion following 22 weeks of HCD and HFD feeding, used as models for diet-induced NAFLD/NASH. (**a**) Representative western blot showing TCF7L2, ACC and FAS protein levels in the liver of mice fed an HCD or HFD for 22 weeks. (**b**) Graph showing relative protein levels of TCF7L2, ACC and FAS in the liver of mice fed an HCD (*n*=4) or HFD (*n*=7) for 22 weeks compared with a normal chow diet (NCD; *n*=3). *p<0.05, **p<0.01, 22 weeks vs 0 weeks, analysed by unpaired Student’s *t* test. (**c**–**e**) Six-week-old *Tcf7l2*^*f/f*^ and *Alb-Cre;Tcf7l2*^*f/f*^ mice were fed an NCD for 22 weeks (*n*=4 for both genotypes; control groups), or were given an HCD (*n*=7 for both genotypes) or an HFD for 22 weeks (*Tcf7l2*^*f/f*^, *n*=7; *Alb-Cre;Tcf7l2*^*f/f*^, *n*=5) to induce NAFLD progression. Subsequently, frozen liver sections were stained using Oil Red O and H&E (representative images are shown; ×20 magnification; scale bars, 200 μm; **c**), and hepatic TG levels (**d**) and plasma alanine aminotransferase (ALT) and aspartate aminotransferase (AST) levels (**e**) were measured. (**f**) Six-week-old *Tcf7l2*^*f/f*^ (*n*=9) and *Alb-Cre;Tcf7l2*^*f/f*^ mice (*n*=11) were fed an HCD for 22 weeks. Subsequently, intra-abdominal liver images were taken (representative images shown). (**g**, **h**) Six-week-old *Tcf7l2*^*f/f*^ (*n*=9) and *Alb-Cre;Tcf7l2*^*f/f*^ mice (*n*=11) were fed an HCD for 22 weeks. Following this, a GTT (1.5 g/kg body weight; **g**) and ITT (1 U/kg body weight; **h**) were conducted. Glucose AUCs are also presented for both GTT and ITT. *p<0.05, **p<0.01, ***p<0.001, *Alb-Cre;Tcf7l2*^*f/f*^ vs wild-type mice at same time point, analysed by unpaired Student’s *t* test. (**i**) Six-week-old *Tcf7l2*^*f/f*^ (*n*=8) and *Alb-Cre;Tcf7l2*^*f/f*^ (*n*=9) mice were fed an HCD for 22 weeks. Mice were fasted for 6 h and then injected intraperitoneally with insulin (10 U/kg body weight) or saline for 10 min. Presented is a representative western blot showing the effects of hepatic *Tcf7l2* deficiency on the hepatic insulin signalling pathway. The p-Akt/Akt ratio is also shown. HSP90, heat shock protein 90. Key in (**d**) also applies to (**e**–**h**). Data in (**b**) and (**i**) are presented as mean±SD; data in (**d**) (**e**), (**g**) and (**h**) are presented as mean±SEM. *p<0.05, **p<0.01, ***p<0.001, analysed by *t* test

In rodents, studies have shown that feeding with an HFD or HCD for 22 weeks leads to the development of NAFLD, including non-alcoholic fatty liver (NAFL; simple steatosis) and NASH [28–30]. As with individuals with NAFLD, *Tcf7l2* expression was markedly decreased to below-normal levels in the liver of C57BL/6N mice fed an HFD or HCD for 22 weeks (Fig. 2a,b). To assess the role of TCF7L2 in the development of NAFLD, *Alb-Cre;Tcf7l2*^*f/f*^ mice were fed an HFD or HCD for 22 weeks (Fig. 2c). Although hepatic TCF7L2 expression was decreased in the liver of wild-type *Tcf7l2*^*f/f*^ mice by both HFD and HCD feeding for 22 weeks, hepatic TCF7L2 expression in wild-type *Tcf7l2*^*f/f*^ mice was clearly higher compared with that in *Alb-Cre;Tcf7l2*^*f/f*^ mice (ESM Fig. 2a). Under HCD conditions, hepatic *Tcf7l2* deficiency promoted lipid droplet formation and TG accumulation in the liver, resulting in elevated alanine aminotransferase (ALT) levels (Fig. 2c–e). In contrast, these differences were not observed when mice were fed an HFD (Fig. 2c–e). Consistently, genes responsible for lipogenesis and inflammation in NAFLD/NASH were found to be significantly increased in the liver of *Alb-Cre;Tcf7l2*^*f/f*^ mice, as compared with wild-type mice, following HCD feeding, whilst elevation of these genes was less consistently observed following HFD feeding (ESM Fig. 2b,c). However, there were no significant changes in islet size or islet glucagon-positive alpha cell and insulin-positive beta cell areas in mice fed an HFD or HCD (ESM Fig. 2d-g). Representative liver images of *Alb-Cre;Tcf7l2*^*f/f*^ mice fed an HCD for 22 weeks are shown in Fig. 2f. In response to 22 weeks of HCD, hepatic *Tcf7l2* deficiency caused impairment of glucose and insulin tolerance (Fig. 2g,h). This impeded hepatic insulin signalling, as confirmed by the reduced level of Akt phosphorylation on Ser473 in the liver of *Alb-Cre;Tcf7l2*^*f/f*^ mice vs wild-type mice (Fig. 2i). However, it did not affect Akt phosphorylation levels in epididymal white adipose tissue and skeletal muscle (ESM Fig. 2h).

### TCF7L2 does not affect HFD-induced intrahepatic TG accumulation

Compared with an HCD, an HFD did not have as marked an effect on NAFLD development in hepatic *Tcf7l2*-deficient mice. To investigate whether this could have been owing to saturation of liver lipid content under an HFD diet, we used *Alb-Cre;Tcf7l2*^*f/f*^ mice fed an HFD for 4 or 12 weeks. Hepatic *Tcf7l2* depletion exacerbated HFD-induced glucose intolerance and insulin intolerance without causing changes in body weight (ESM Fig. 3a–c). However, it did not alter hepatic TG content or plasma TG and NEFA levels (ESM Fig. 3d–g). To specifically address why hepatic *Tcf7l2* did not affect HFD-induced TG content, we analysed the expression of genes involved in various lipid-related metabolic pathways (ESM Fig. 3h). Notably, hepatic *Tcf7l2* deficiency increased the expression of several lipogenic genes (*Srebf1c* [encoded by the *Srebf1* gene]*, Fasn* and *Acaca*), whereas it decreased the expression of several fatty acid transporter genes (*Cd36*, *Slc27a1*, *Slc27a4* and *Fabp3*; ESM Fig. 3h). In contrast, adenovirus-mediated expression of hepatic *Tcf7l2* inversely regulated many of these genes (*Srebf1c*, *Fasn*, *Cd36*, *Slc27a4* and *Fabp3*) without altering HFD-induced hepatic TG content (ESM Fig. 3i,j). Further, in a cell-autonomous manner, *Tcf7l2* KO via Cre deletion of floxed sequences in primary hepatocytes impeded insulin-induced fatty acid uptake, as confirmed by use of a fluorescent fatty acid probe (ESM Fig. 4a), whereas forced expression of *Tcf7l2* in C57BL/6N primary mouse hepatocytes promoted it (ESM Fig. 4h). It is well known that HFD suppresses DNL [31]. Accordingly, fatty acids can inhibit insulin-stimulated glucose uptake [32, 33]. Therefore, we hypothesised that hepatic *Tcf7l2* deficiency did not affect HFD-induced liver lipid content, despite there being a decrease in fatty acid uptake, because lipogenesis is balanced by a relative increase in glucose-sensing in response to reduced fatty acid uptake. Indeed, *Tcf7l2* KO in primary hepatocytes impaired fatty acid-induced inhibition of glucose uptake (ESM Fig. 4b). Expression of L-type pyruvate kinase (L-PK; encoded by the *pklr* gene) and *Pklr*, a glucose-sensing marker, increased in the liver of *Alb-Cre;Tcf7l2*^*f/f*^ mice vs wild-type mice, even under HFD conditions (ESM Fig. 4c,d).

Fatty acid β-oxidation, another lipid-associated metabolic pathway, was also altered by hepatic *Tcf7l2* modulation under HFD conditions (ESM Fig. 3h,j). The AMP-activated protein kinase (AMPK)/acetyl-CoA carboxylase (ACC)/carnitine palmitoyltransferase 1α (CPT1α) axis is a key pathway for regulating fatty acid oxidation [34]. However, neither KO nor overexpression of *Tcf7l2* functionally affected fatty acid β-oxidation, as determined by changes in OCR following treatment with fatty acid and/or etomoxir, an irreversible inhibitor of CPT1α (ESM Fig. 4e,i). Further, *Tcf7l2* KO or overexpression did not affect AMPK phosphorylation levels following treatment with 5-aminoimidazole-4-carboxamide ribonucleotide (AICAR), a selective activator of AMPK, under HFD conditions (ESM Fig. 4f,g,j,k).

### Hepatic TCF7L2 regulates hepatic DNL in a cell-autonomous fashion

**Fig. 3:**
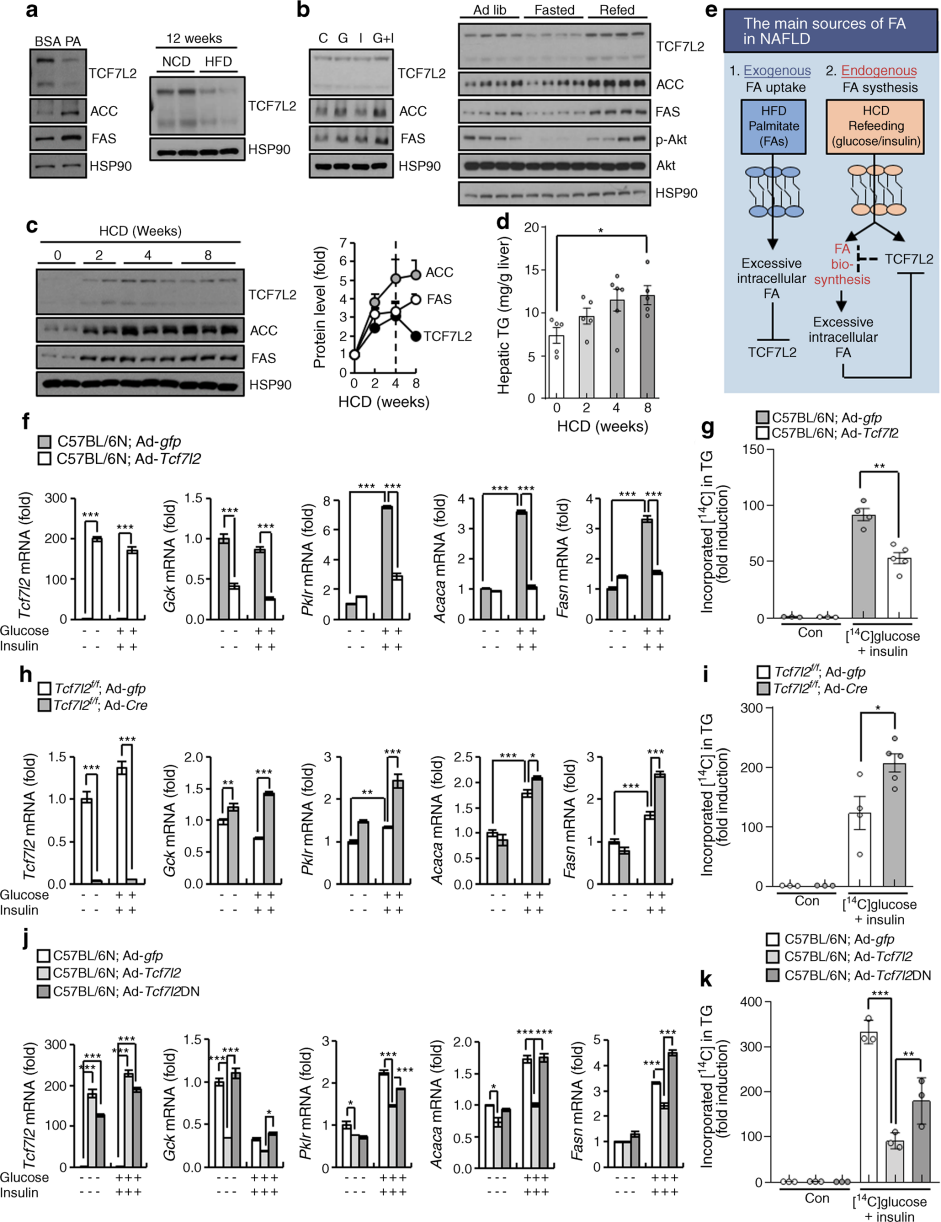
Cell-autonomous role of hepatic TCF7L2 in regulating DNL. (**a**) Representative western blots showing protein levels of TCF7L2, ACC and FAS in mouse primary hepatocytes treated with BSA or 250 μmol/l palmitic acid (PA) for 24 h (data are representative of *n*=3 independent experiments) and TCF7L2 protein levels in the liver of C57BL/6N mice fed an HFD (*n*=4) or a normal chow diet (NCD; *n*=4) for 12 weeks. (**b**) Representative western blots showing protein levels of TCF7L2, ACC and FAS in mouse primary hepatocytes treated with 25 mmol/l glucose (G) and/or 10 nmol/l insulin (I) or saline (control [C]) for 48 h (data are representative of *n*=3 independent experiments), and TCF7L2, ACC, FAS, p-Akt and Akt levels in the liver of 10-week old C57BL/6N mice under ad libitum (ad lib; *n*=4), 24 h fasted (*n*=4) or 24 h refed (*n*=4) conditions. Quantification data are presented in ESM Fig. 5a and ESM Fig. 5c. (**c**, **d**) C57BL/6N mice were fed an HCD for 2, 4 and 8 weeks (*n*=5 per group; for this experiment, at '0 weeks of HCD feeding', *n*=5 mice that had been fed an NCD for 8 weeks were used). Representative western blot showing TCF7L2, ACC and FAS protein levels is shown alongside the quantified relative protein levels (TCF7L2/heat shock protein 90 [HSP90], ACC/HSP90 and FAS/HSP90 ratios) (**c**; further quantification data, including details of statistical significance, are presented in ESM Fig. 5d). Hepatic TG levels are also presented (**d**). *p<0.05, analysed by one-way ANOVA with Tukey’s post hoc test. (**e**) Schematic diagram showing the hypothesised function of hepatocyte TCF7L2 in hepatic lipid metabolism. FA, fatty acid. (**f**) Primary hepatocytes were isolated from 10-week-old C57BL/6N mice. Cells were infected with adenovirus expressing *Tcf7l2* (Ad-*Tcf7l2*) or Ad-*gfp* and treated with 25 mmol/l glucose and 10 nmol/l insulin for 48 h. Subsequently, quantitative PCR (qPCR) analysis of *Tcf7l2* and glycolytic (*Gck* and *Pklr*) and lipogenic (*Acaca* and *Fasn*) genes was conducted (*n*=3 per group). (**g**) Primary hepatocytes were isolated from 10-week-old C57BL/6N mice. Cells were infected with Ad-*gfp* or Ad-*Tcf7l2* and treated with 37 kBq of [^14^C]glucose and 10 nmol/l insulin or 3% (vol./vol.) ethanol in saline (control [Con]) for 48 h. Incorporation of [^14^C]glucose into TG was measured to determine DNL rate. (**h**) Primary hepatocytes were isolated from 10-week-old *Tcf7l2*^*f/f*^ mice. Cells were infected with adenovirus expressing *Cre* (Ad-*Cre*) or Ad-*gfp* and treated with 25 mmol/l glucose and 10 nmol/l insulin for 48 h. Subsequently, qPCR analysis of *Tcf7l2* and glycolytic and lipogenic gene expression was conducted (*n*=3 per group). (**i**) Primary hepatocytes were isolated from 10-week-old *Tcf7l2*^*f/f*^ mice. Cells were infected with Ad-*gfp* or Ad-*Cre* and treated with 37 kBq of [^14^C]glucose and 10 nmol/l insulin or 3% (vol./vol.) ethanol in saline (Con) for 48 h. Incorporation of [^14^C]glucose into TG was measured to determine DNL rate. (**j**) Primary hepatocytes were isolated from 10-week-old C57BL/6N mice. Cells were infected with Ad-*gfp*, adenovirus expressing wild-type *Tcf7l2* (Ad-*Tcf7l2*) or adenovirus expressing *Tcf7l2* DN mutant (Ad-*Tcf7l2*DN) and treated with 25 mmol/l glucose and 10 nmol/l insulin for 48 h. Subsequently, qPCR analysis of *Tcf7l2* and glycolytic and lipogenic gene expression was conducted (*n*=3 per group). *p<0.05, ***p<0.001, analysed by one-way ANOVA with Tukey’s post hoc test. (**k**) Primary hepatocytes were isolated from 10-week-old C57BL/6N mice. Cells were infected with Ad-*gfp*, Ad-*Tcf7l2*WT or Ad-*Tcf7l2*DN and treated with 37 kBq of [^14^C]glucose and 10 nmol/l insulin or 3% (vol./vol.) ethanol in saline (Con) for 48 h. Incorporation of [^14^C]glucose into TG was measured to determine DNL rate. **p<0.01, ***p<0.001, analysed by one-way ANOVA with Tukey’s post hoc test. Data in (**c**), (**d**), (**g**) and (**i**–**k**) are presented as mean±SEM; data in (**f**) and (**h**) are presented as mean±SD. *p<0.05, **p<0.01, ***p<0.001, analysed by *t* test, unless stated otherwise

HFD provides excessive exogenous fatty acids, whereas HCD and refeeding mechanistically stimulate endogenous fatty acid synthesis from glucose by postprandial blood glucose and glucose-responsive insulin action, which are increased following carbohydrates consumption. Therefore, we hypothesised that the difference in effects of TCF7L2 on HCD- and HFD-induced fatty liver might originate from differential responses to exogenous fatty acid and glucose/insulin pools. Hepatic TCF7L2 expression decreased in primary hepatocytes treated with palmitic acid, the most common saturated fatty acid in the human body, and in the liver of HFD-fed mice (Fig. 3a). Conversely, hepatic TCF7L2 expression significantly increased following in vitro treatment with glucose/insulin and following in vivo refeeding (Fig. 3b and ESM Fig. 5a**–**c). To further investigate the relationship between TCF7L2 expression and carbohydrate sensing, we measured hepatic TCF7L2 expression at the same time as measuring the expression of lipogenic factors over varying HCD exposure time. Hepatic TCF7L2 expression increased after 2 and 4 weeks of HCD feeding vs baseline but was downregulated after 8 weeks of HCD feeding (Fig. 3c and ESM Fig. 5d,e). However, expression of the lipogenic factors ACC (encoded by *Acaca*) and fatty acid synthase (FAS; encoded by *Fasn*), and hepatic TG levels increased with HCD feeding (Fig. 3c,d and ESM Fig. 5d,e). Eventually, as shown in Fig. 2a, hepatic TCF7L2 expression decreased to below-normal levels after 22 weeks of HCD feeding, at which point excessive intrahepatic fatty acid was expected.

Hence, we hypothesised that although excessive increases in intrahepatic fatty acid as a result of long-term HCD feeding can eventually reduce hepatic TCF7L2 expression and activity, hepatic TCF7L2 restrains endogenous fatty acid synthesis to maintain lipid homeostasis (Fig. 3e). Therefore, we investigated whether TCF7L2 could regulate hepatic DNL in a cell-autonomous fashion. Adenovirus-mediated *Tcf7l2* expression suppressed the expression of glucose-/insulin-stimulated DNL genes involved in glycolysis (*Gck* and *Pklr*) and lipogenesis (*Acaca* and *Fasn*) and inhibited DNL in primary hepatocytes, as compared with control cells infected with Ad-*gfp* (Fig. 3f,g). Conversely, *Tcf7l2*^*f/f*^ primary hepatocytes infected with adenovirus expressing *Cre* (Ad-*Cre*) exhibited increased DNL genes and promoted DNL, as compared with cells infected with Ad-*gfp* (Fig. 3h,i). Consistent with previous reports, *Tcf7l2* deficiency did not significantly affect hepatic insulin signalling pathway in a cell-autonomous manner (ESM Fig. 5f). Further, to investigate *Tcf7l2* functional knockdown whilst avoiding potential redundancy of other TCF protein members, we generated a TCF7L2 dominant-negative (DN) mutant (TCF7L2DN) that lacks the N-terminal β-catenin interaction domain. TCF7L2DN inhibited the suppressive effects of TCF7L2 on DNL-associated genes and DNL in primary hepatocytes (Fig. 3j,k).

### Hepatic *Tcf7l2* depletion increases hepatic DNL in a time- and amount-dependent manner following carbohydrate loading

**Fig. 4:**
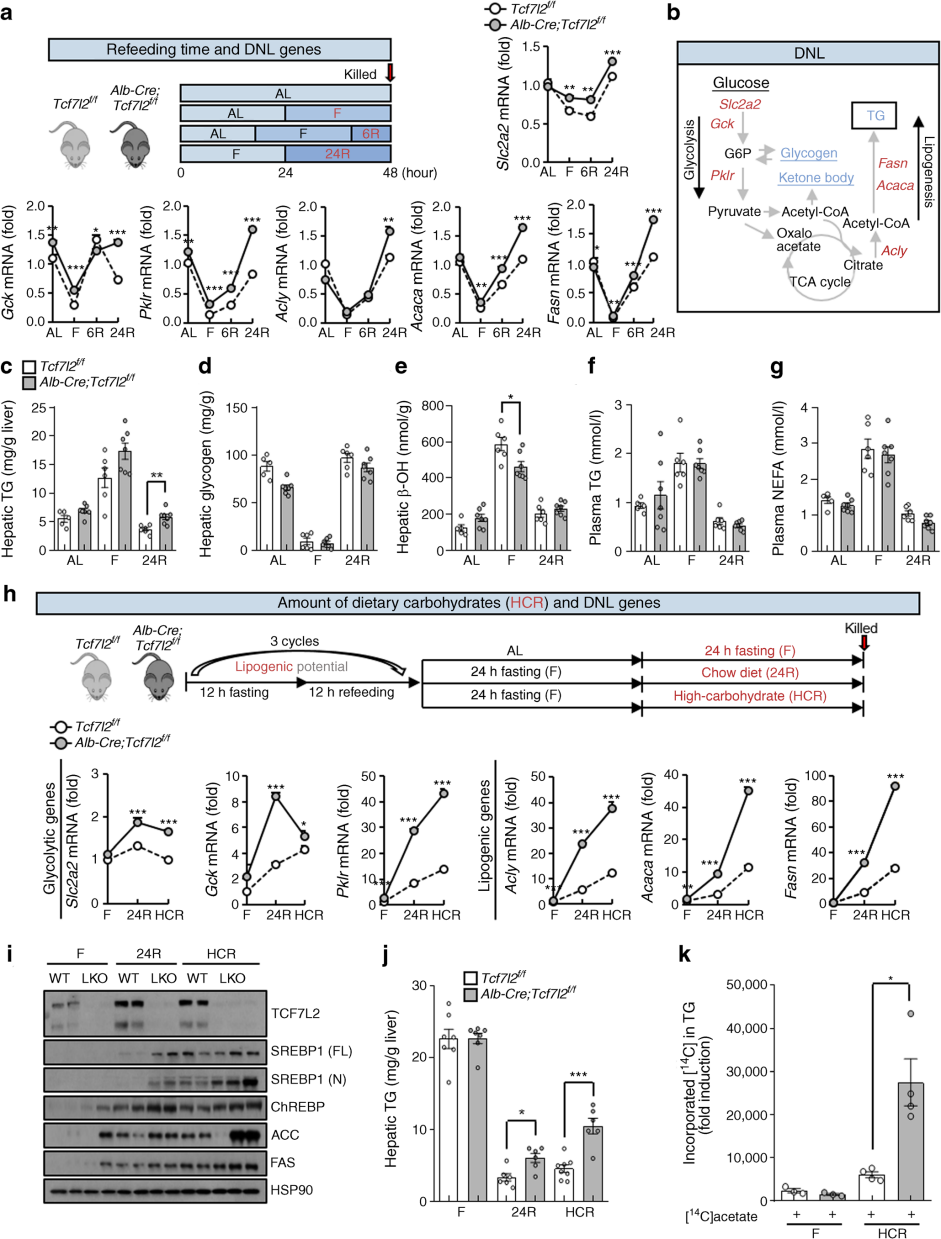
Changes in intrahepatic DNL levels in *Alb-Cre;Tcf7l2*^*f/f*^ mice following carbohydrate loading. (**a**) Ten-week-old *Tcf7l2*^*f/f*^ (*n*=5) and *Alb-Cre;Tcf7l2*^*f/f*^ (*n*=7) mice were fed regular chow ad libitum (AL; *Tcf7l2*^*f/f*^, *n*=5; *Alb-Cre;Tcf7l2*^*f/f*^, *n*=7), were fasted for 24 h (F; *Tcf7l2*^*f/f*^, *n*=6; *Alb-Cre;Tcf7l2*^*f/f*^, *n*=7), or were fasted for 24 h and refed for either 6 h (6R; *Tcf7l2*^*f/f*^, *n*=6; *Alb-Cre;Tcf7l2*^*f/f*^, *n*=7) or 24 h (24R; *Tcf7l2*^*f/f*^, *n*=6; *Alb-Cre;Tcf7l2*^*f/f*^, *n*=7). (**a**) The schematic diagram shows the experimental design. Also shown is quantitative PCR (qPCR) analysis of hepatic expression of glycolytic and lipogenic genes in mouse livers. *p<0.05, **p<0.01, ***p<0.001 *Alb-Cre;Tcf7l2*^*f/f*^ vs wild-type mice under the same conditions, analysed by unpaired Student’s *t* test. (**b**) Schematic diagram of the DNL pathway. Red text, DNL-associated genes; blue text, metabolites associated with DNL. G6P, glucose 6-phosphate; TCA, tricarboxylic acid. (**c–g**) Ten-week-old *Tcf7l2*^*f/f*^ and *Alb-Cre;Tcf7l2*^*f/f*^ mice were fed regular chow ad libitum (AL; *Tcf7l2*^*f/f*^, *n*=5; *Alb-Cre;Tcf7l2*^*f/f*^, *n*=7), were fasted for 24 h (F; *Tcf7l2*^*f/f*^, *n*=6; *Alb-Cre;Tcf7l2*^*f/f*^, *n*=7), or were fasted for 24 h and refed for either 6 h (6R; *Tcf7l2*^*f/f*^, *n*=6; *Alb-Cre;Tcf7l2*^*f/f*^, *n*=7) or 24 h (24 R; *Tcf7l2*^*f/f*^, *n*=6; *Alb-Cre;Tcf7l2*^*f/f*^, *n*=7). Levels of hepatic TG (**c**), hepatic glycogen (**d**), hepatic β-OH (**e**), plasma TG (**f**) and plasma NEFA (**g**) were measured. (**h–j**) Ten-week-old *Tcf7l2*^*f/f*^ and *Alb-Cre;Tcf7l2*^*f/f*^ mice were fasted for 24 h (F; *n*=7 for both genotypes), were fasted for 24 h and refed with a chow diet for 24 h (24R; *Tcf7l2*^*f/f*^, *n*=7; *Alb-Cre;Tcf7l2*^*f/f*^, *n*=6) or fasted for 24 h and refed an HCR for 24 h (*Tcf7l2*^*f/f*^, *n*=8; *Alb-Cre;Tcf7l2*^*f/f*^, *n*=6). The fasting-feeding cycle was repeated three times at 12 h intervals prior to study. A schematic diagram showing the experimental design is shown alongside qPCR analysis of glycolytic and lipogenic gene expression in mouse liver; *p<0.05, **p<0.01, ***p<0.001 *Alb-Cre;Tcf7l2*^*f/f*^ vs wild-type mice under the same conditions, analysed by unpaired Student’s *t* test (**h**). Representative western blot showing protein levels of TCF7L2, SREBP1 (full length [FL; 125 kDa] and nuclear [N; 60–70 kDa] forms, observed on the same blot), ChREBP, ACC and FAS in liver of *Alb-Cre;Tcf7l2*^*f/f*^ (LKO) and *Tcf7l2*^*f/f*^ (WT) mice (**i**). Hepatic TG levels (**j**). (**k**) The rate of hepatic DNL in *Tcf7l2*^*f/f*^ and *Alb-Cre;Tcf7l2*^*f/f*^ mice subjected to fasting for 24 h (*n*=3 for both genotypes) or HCR for 12 h (*n*=4 for both genotypes) and injected with [^14^C]acetate for 1 h. HSP90, heat shock protein 90. Key in (**j**) also applies to (**k**). Data in (**a**) and (**h**) are presented as mean±SD; data in (**c–g**), (**j**) and (**k**) are presented as mean±SEM. *p<0.05, **p<0.01, ***p<0.001, analysed by *t* test

To further assess the physiological function of hepatic *Tcf7l2* deficiency in hepatic DNL, *Alb-Cre;Tcf7l2*^*f/f*^ mice were fasted and then refed. Compared with wild-type mice, this promoted the expression of DNL genes involved in glycolysis (*Slc2a2*, *Gck* and *Pklr*) and lipogenesis (*Acly*, *Acaca* and *Fasn*) following refeeding in a time-dependent manner (Fig. 4a). These genes are shown (in red) in Fig. 4b, which depicts the DNL process. However, there were no changes in the expression of genes involved in lipolysis and β-oxidation upon refeeding (ESM Fig. 6). Importantly, loss of hepatic *Tcf7l2* led to increased liver TG content after 24 h of refeeding, but not levels of hepatic glycogen and β-OH, which are glucose products that are dispersed into and metabolised by other pathways during the DNL process (Fig. 4c–e). Additionally, there were no changes in plasma TG and NEFA levels (Fig. 4f,g). These data suggest that the activation of the glycolytic/lipogenic pathway for DNL is the primary factor that affects fat deposition in the liver of *Alb-Cre;Tcf7l2*^*f/f*^ mice.

Dietary carbohydrates are the primary stimulus for hepatic DNL. To further elucidate the carbohydrate dependency of *Tcf7l2* deficiency-induced hepatic TG accumulation, we compared normal chow refeeding with high-carbohydrate refeeding (HCR) in *Alb-Cre;Tcf7l2*^*f/f*^ mice. Hepatic *Tcf7l2* deficiency promoted expression of glycolytic and lipogenic factors and hepatic TG accumulation, which was more evident under HCR conditions compared with normal chow refeeding conditions (Fig. 4h–j). A high-carbohydrate intake markedly enhanced hepatic DNL induced by hepatic *Tcf7l2* depletion (Fig. 4k). These data suggest that hepatic *Tcf7l2* deficiency can aggravate liver steatosis and that the extent to which this occurs is dependent on carbohydrate intake, owing to increased hepatic DNL with carbohydrate loading.

### SREBP1c and ChREBP are core factors associated with lipid metabolism in hepatic *Tcf7l2*-deficient mice

**Fig. 5:**
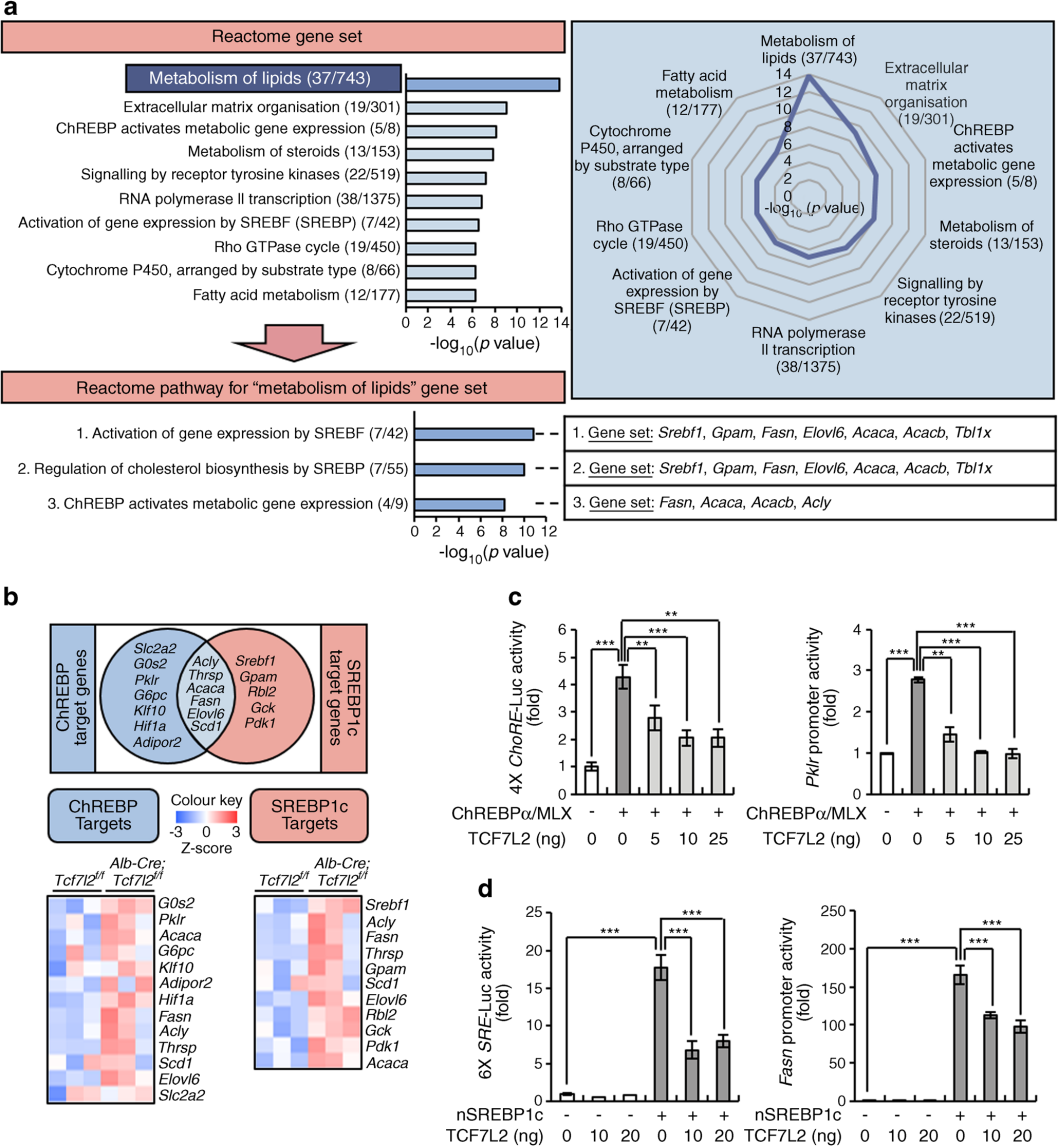
TCF7L2 regulates hepatic lipid metabolism by targeting SREBP1c and ChREBP pathways. (**a**) Comparative analysis of mRNA-sequencing data from liver samples from *Tcf7l2*^*f/f*^ and *Alb-Cre;Tcf7l2*^*f/f*^ mice refed a normal chow diet for 24 h (*n*=3 per group). Reactome enrichment analysis of 526 upregulated genes (log_2_ fold-change cut-off >1.5) in *Alb-Cre;Tcf7l2*^*f/f*^ mice compared with *Tcf7l2*^*f/f*^ mice is shown. The radial line graph relates to the data for the Reactome gene set. In addition, data for the major pathways relating to the most significantly altered gene set (‘metabolism of lipids’; see ESM Table 6 for complete gene set) are presented. Values in brackets are overlap/input numbers. Rho, Ras homologous; SREBF, sterol regulatory element-binding transcription factor (also known as sterol regulatory element binding protein [SREBP]). (**b**) Venn diagram showing SREBP1c and ChREBP target genes in liver samples from *Tcf7l2*^*f/f*^ and *Alb-Cre;Tcf7l2*^*f/f*^ mice refed a normal chow diet for 24 h (*n*=3 per group). Heatmaps visualise hepatic expression of SREBP1c and ChREBP target genes in mice (see ESM Table 7 for list of genes). (**c**, **d**) Luciferase (Luc) reporter assay data showing the effects of TCF7L2 expression on ChREBPα/MLX-induced activation of 4× ChoRE and *Pklr* promoters (**c**) and the effects of TCF7L2 expression on nuclear SREBP1c (nSREBP1c)-induced activation of 6× SRE and *Fasn* promoters (**d**) in the HEPG2 cell line (*n*=3 per group). Data in (**c**) and (**d**) are presented as mean±SEM. **p<0.01, ***p<0.001, analysed by one-way ANOVA with Tukey’s post hoc test

To investigate the major pathways and key instigators upregulated in the liver of *Alb-Cre;Tcf7l2*^*f/f*^ mice, we analysed mRNA-sequencing data from liver samples from refed mice. In total, 526 genes were upregulated (log_2_ fold-change cut-off >1.5) and were analysed using GSEA (www.gsea-msigdb.org/gsea/msigdb/mouse/annotate.jsp, accessed on 1 November 2021), based on canonical pathways gene sets derived from the Reactome pathway database (www.gsea-msigdb.org/gsea/msigdb/mouse/genesets.jsp?collection=CP:REACTOME, accessed on 1 November 2021). The lipid metabolism-related gene set was most significantly upregulated (its significance can be more intuitively and integratedly presented as a radial line graph; see Fig. 5a and ESM Table 6). Specifically, the SREBP- and ChREBP-related pathways were identified as major signalling pathways (Fig. 5a). Careful investigation of mRNA-sequencing data showed that specific target genes and commonly shared lipogenic target genes of SREBP1c and ChREBP increased in the liver of *Alb-Cre;Tcf7l2*^*f/f*^ mice (Fig. 5b and ESM Table 7). However, despite a reduction in *Srebf2* mRNA expression in the liver of *Alb-Cre;Tcf7l2*^*f/f*^ vs wild-type mice, the expression of its target cholesterogenic genes was not altered (ESM Fig. 7a,b and ESM Table 8). SREBP1c and ChREBP are the main transcription factors for DNL [35] that regulate target gene transcription by binding to the sterol regulatory element (SRE) and carbohydrate response element (ChoRE) within their target gene promoters, respectively [36]. In the HEPG2 cell line, transfection with TCF7L2 inhibited ChREBP/MLX-induced activation of *Pklr* promoters containing ChoRE as well as ChREBP/MLX-induced activation of the ChoRE promoter itself (Fig. 5c). Furthermore, TCF7L2 inhibited SREBP1c-induced activation of an *Fasn* promoter region containing the SRE only as well as SREBP1c-induced activation of the SRE promoter itself (Fig. 5d). Liver X receptors (LXRs) are nuclear receptors that can directly regulate both SREBP1c and ChREBP [35]. However, neither KO nor overexpression of *Tcf7l2* in primary hepatocytes affected lipogenic gene expression induced by the LXR agonist T0901317 (ESM Fig. 7c,d). These data suggest that TCF7L2 regulates ChREBP and SREBP1c transcriptional activities in an LXR-independent manner.

### TCF7L2 promotes ChREBP transcriptional activity by modulating *O*-GlcNAcylation and ChREBP protein stability

**Fig. 6:**
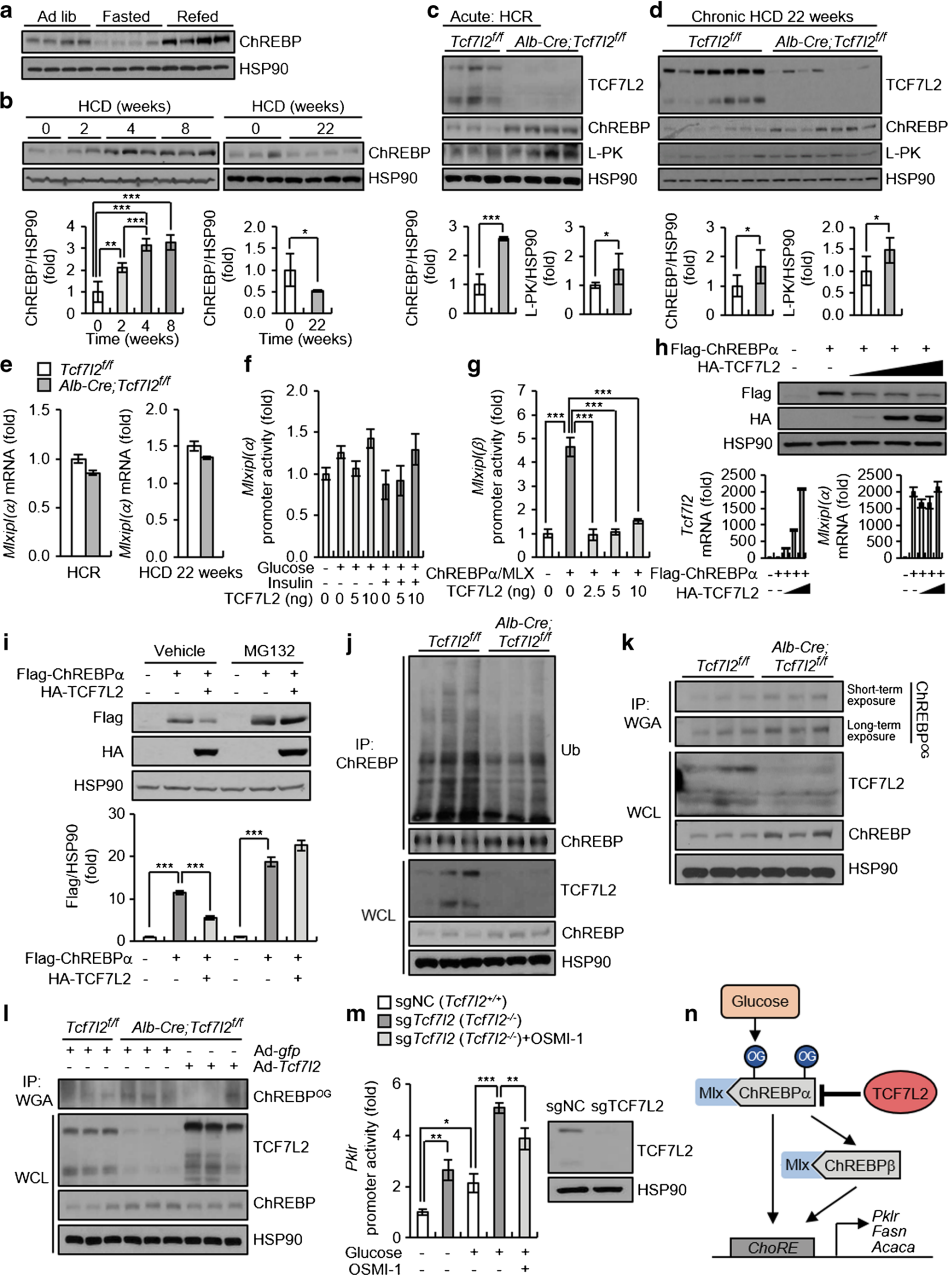
TCF7L2 regulates ChREBP transcriptional activity by modulating *O*-GlcNAcylation and protein stability of ChREBP. (**a**) Representative western blot showing protein levels of ChREBP in the liver of 10-week old C57BL/6N mice under ad libitum feeding (ad lib; *n*=4), 24 h fasted (*n*=4) or 24 h refed (*n*=4) conditions. (**b**) C57BL/6N mice were fed a an HCD for 2, 4 and 8 weeks (*n*=5 per group; for this experiment, at ‘0 weeks of HCD feeding’, *n*=5 mice that had been fed a normal chow diet [NCD] for 8 weeks were used) or they were fed an HCD for 22 weeks (*n*=4; for this experiment, at ‘0 weeks of HCD feeding’, *n*=3 mice that had been fed an NCD for 22 weeks were used). Representative western blot showing ChREBP protein levels in mouse livers is shown alongside the ChREBP/heat shock protein 90 (HSP90) ratio. **p<0.01, ***p<0.001, analysed by one-way ANOVA with Tukey’s post hoc test. (**c**) Ten-week-old *Tcf7l2*^*f/f*^ (*n*=8) and *Alb-Cre;Tcf7l2*^*f/f*^ (*n*=6) mice were subjected to HCR for 24 h. Representative western blots of protein levels of TCF7L2, ChREBP and L-PK in the liver are shown. ChREBP/HSP90 and L-PK/HSP90 ratios are also shown. (**d**) Six-week-old *Tcf7l2*^*f/f*^ (*n*=7) and *Alb-Cre;Tcf7l2*^*f/f*^ (*n*=7) mice were fed an HCD for 22 weeks. Representative western blots of protein levels of TCF7L2, ChREBP and L-PK in the liver are shown. ChREBP/HSP90 and L-PK/HSP90 ratios are also shown. (**e**) Ten-week-old *Tcf7l2*^*f/f*^ (*n*=8) and *Alb-Cre;Tcf7l2*^*f/f*^ (*n*=6) mice were subjected to HCR for 24 h or 6-week-old *Tcf7l2*^*f/f*^ (*n*=7) and *Alb-Cre;Tcf7l2*^*f/f*^ (*n*=7) mice were fed an HCD for 22 weeks. Quantitative PCR (qPCR) analysis of mRNA expression levels of *Mlxipl(α)* in the liver is shown. (**f**) Luciferase reporter assay showing the effects of TCF7L2 expression and 25 mmol/l glucose and/or 10 nmol/l insulin treatment on *Mlxipl(α)* promoter activity in the HEPG2 cell line (*n*=3 replicates). Data are representative of *n*=3 independent experiments. (**g**) Luciferase reporter assay showing the effects of TCF7L2 expression on ChREBPα/MLX-induced activation of the *Mlxipl(β)* promoter in the HEPG2 cell line (*n*=3 replicates). Data are representative of *n*=3 independent experiments. ***p<0.001, assessed by one-way ANOVA with Tukey’s post hoc test. (**h**) HEPG2 cells were transfected with 500 ng of Flag-tagged ChREBPα and various concentrations (250 ng, 500 ng or 1000 ng) of haemagglutinin (HA)-tagged TCF7L2 for 48 h (*n*=3 replicates). A representative western blot showing changes in Flag-tagged ChREBPα protein levels according to expression levels of HA-tagged TCF7L2 is presented. qPCR analysis of mRNA levels of *Tcf7l2* and *Mlxipl(α)* is also shown. Data are representative of *n*=3 independent experiments. (**i**) HEPG2 cells were transfected with HA-tagged TCF7L2 and Flag-tagged ChREBPα for 48 h and treated with 10 μmol/l MG132 or DMSO (vehicle) for 3 h (*n*=3 replicates). A representative western blot showing changes in ChREBPα protein stability by TCF7L2 expression is presented. Quantification of Flag-tagged ChREBPα is also shown. Data are representative of *n*=3 independent experiments. ***p<0.001, analysed by one-way ANOVA with Tukey’s post hoc test. (**j**) *Tcf7l2*^*f/f*^ (*n*=3) and *Alb-Cre;Tcf7l2*^*f/f*^ (*n*=3) mice were subjected to HCR for 24 h. Liver protein samples were immunoprecipitated with anti-ChREBP and a representative western blot of protein levels of ubiquitin (Ub), ChREBP, and TCF7L2 is shown. IP, immunoprecipitation; WCL, whole-cell lysate. Quantification data are presented in ESM Fig. 8a. (**k**) *Tcf7l2*^*f/f*^ (*n*=3) and *Alb-Cre;Tcf7l2*^*f/f*^ (*n*=3) mice were subjected to HCR for 24 h. Liver protein samples were immunoprecipitated with anti-WGA and a representative western blot showing protein levels of *O*-GlcNAcylated ChREBP (ChREBP^*O*G^), TCF7L2 and ChREBP is shown. The blots were given various film exposure times to clarify the protein bands (short-term exposure, 1 min; long-term exposure, 5 min). IP, immunoprecipitation; WCL, whole-cell lysate. Quantification data are presented in ESM Fig. 8b. (**l**) *Tcf7l2*^*f/f*^ and *Alb-Cre;Tcf7l2*^*f/f*^ mice were infected with Ad-*gfp* or adenovirus expressing *Tcf7l2* (Ad-*Tcf7l2*) via the tail vein and then subjected to HCR for 24 h (*n*=3 per group). Liver protein samples were immunoprecipitated with WGA. A representative western blot showing protein levels of *O*-GlcNAcylated ChREBP (ChREBP^*O*G^), TCF7L2 and ChREBP is shown. IP, immunoprecipitation; WCL, whole-cell lysate. Quantification data (including statistical significance) are presented in ESM Fig. 8c. (**m**) *Tcf7l2* WT (single-guide negative control [sgNC]; *Tcf7l2*^+/+^) and *Tcf7l2* KO (sg*Tcf7l2*; *Tcf7l2*^−/−^) AML12 cell lines were generated using the CRISPR-Cas9 system. Cells were transfected with the pGL4-*Pklr* (−191/+200) promoter and then co-treated with 25 mmol/l glucose and 40 μmol/l OSMI-1 for 24 h (*n*=3 replicates). Luciferase reporter assay data showing the effects of *Tcf7l2* KO and OSMI-1 on *Pklr* promoter activity under high-glucose conditions is shown. A representative western blot showing *Tcf7l2* KO in AML12 cell lines is also shown. Data are representative of *n*=3 independent experiments. *p<0.05, **p<0.01, ***p<0.001, analysed by one-way ANOVA with Tukey’s post hoc test. (**n**) Proposed mechanism underlying TCF7L2-induced regulation of ChREBP transcriptional activity. *O*G, *O*-linked GlcNAc modification. Key in (**e**) also applies to (**c**) and (**d**). Data in (**b**), (**f**), (**g**), (**i**) and (**m**) are presented as mean±SEM; data in (**c**–**e**) and (**h**) are presented as mean±SD. *p<0.05, **p<0.01, ***p<0.001, analysed by *t* test, unless stated otherwise

Among the two ChREBP isoforms (ChREBPα and ChREBPβ; both encoded by the *Mlxipl* gene) [37], the 100 kDa protein band corresponding to ChREBPα [35] was found to be significantly increased in the refed state and after 2, 4 and 8 weeks of HCD, but decreased after 22 weeks of chronic HCD (Fig. 6a,b). However, hepatic *Tcf7l2* deficiency resulted in increased protein expression of ChREBPα and its target L-PK (encoded by the *pklr* gene), not only under acute HCR conditions but also following 22 weeks of chronic HCD (Fig. 6c,d). However, there were no changes in mRNA levels and promoter activity of *Mlxipl(α)* (Fig. 6e,f). Therefore, we investigated whether TCF7L2 regulates ChREBPα protein levels to modulate ChREBPα transcriptional activity. As shown in Fig. 6g, TCF7L2 inhibited ChREBPα/MLX-induced *Mlxipl(β)* promoter activity. In a set of experiments using the same concentration ratio of Flag-tagged ChREBPα:HA-tagged TCF7L2 used in the promoter activity assay, ChREBPα protein levels appeared to gradually decrease in a TCF7L2 expression-level-dependent manner, without changes in *Mlxipl(α)* mRNA expression (Fig. 6h). Further, the TCF7L2-mediated reduction in ChREBPα protein expression was restored by treatment with the proteasome inhibitor MG132 (Fig. 6i). Conversely, ChREBP ubiquitination was found to be significantly decreased in the liver of hepatic *Tcf7l2-*deficient mice under HCR conditions (Fig. 6j and ESM Fig. 8a). *O*-GlcNAcylation of ChREBP is induced by high glucose levels, blocking its ubiquitination to increase glucose utilisation and lipogenic gene transcription [38]. Loss of hepatic *Tcf7l2* significantly increased *O*-GlcNAcylation of ChREBP under HCR conditions, whereas hepatic *Tcf7l2* restoration reduced it to normal levels (Fig. 6k,l and ESM Fig. 8b,c). Consistently, CRISPR/Cas9-mediated *Tcf7l2* KO in the AML12 cell line significantly promoted ChREBPα protein expression and the expression and promoter activity of its target gene *Pklr* in a cell-autonomous manner, under high-glucose conditions (ESM Fig. 9). Indeed, the high-glucose-stimulated promoter activity of the ChREBP target *Pklr* in *Tcf7l2* KO cell lines was suppressed by the *O*-GlcNAc transferase (OGT) inhibitor OSMI-1 (Fig. 6m). These data suggest that TCF7L2 regulates ChREBP transcriptional activity via *O*-GlcNAcylation (Fig. 6n).

### TCF7L2 regulates the transcription of miR-33-5p in the SREBP1c/miR-33-5p axis

**Fig. 7:**
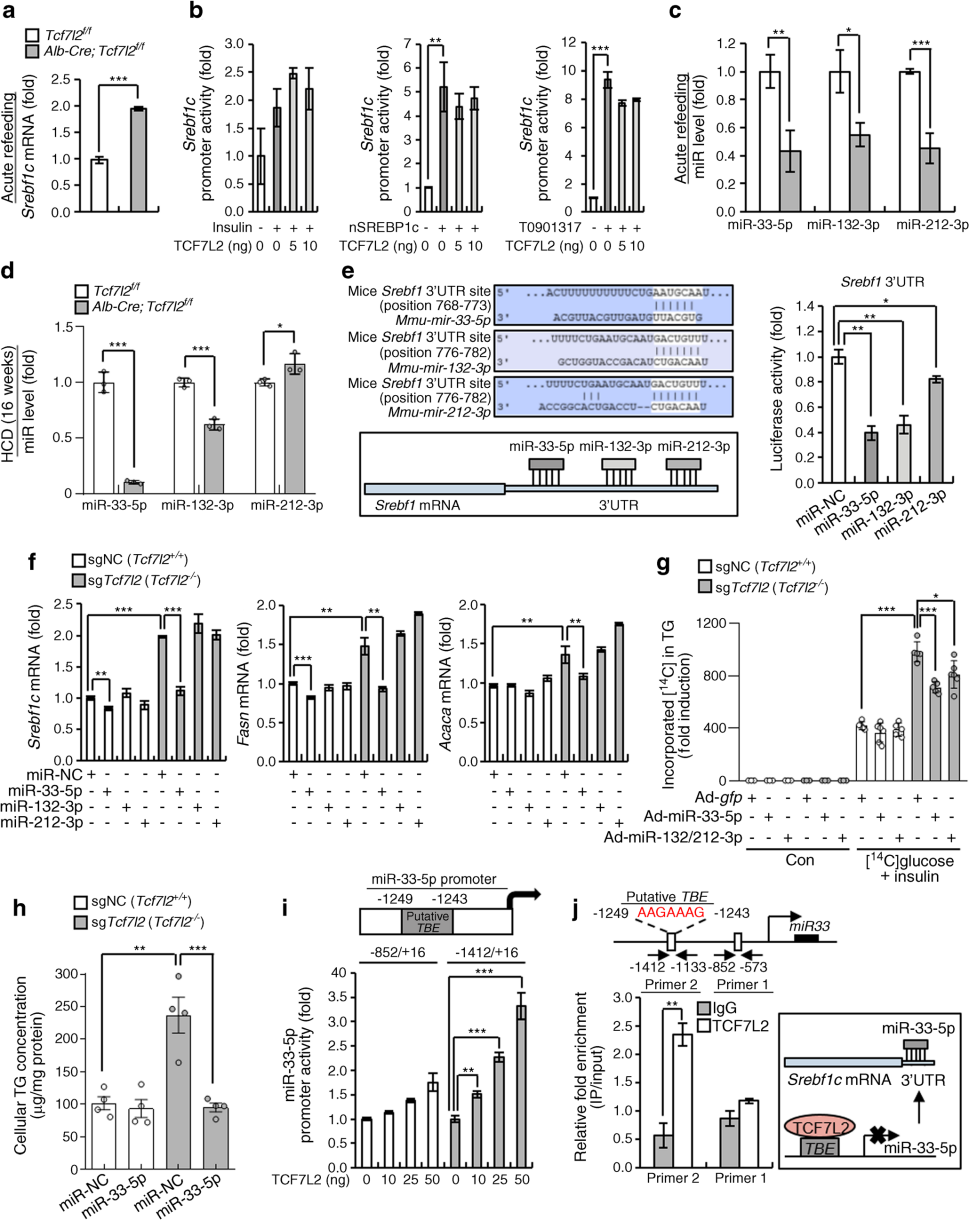
miR-33-5p is a transcriptional target of TCF7L2 in the SREBP1c/miR-33-5p axis. (**a**) Quantitative PCR (qPCR) analysis showing *Srebf1c* mRNA expression in the liver of *Tcf7l2*^*f/f*^ (*n*=6) and *Alb-Cre;Tcf7l2*^*f/f*^ (*n*=7) mice under refeeding conditions. (**b**) Luciferase reporter assay data showing the effects of TCF7L2 expression on *Srebf1c* promoter activity following induction by insulin, nuclear SREBP1c (nSREBP1c) and the LXR agonist T0901317 in the HEPG2 cell line. (**c**) qPCR analysis showing expression levels of miR-33-5p, miR-132-3p and miR-212-3p in the liver of *Tcf7l2*^*f/f*^ (*n*=6) and *Alb-Cre;Tcf7l2*^*f/f*^ (*n*=7) mice under refeeding conditions. (**d**) qPCR analysis showing expression levels of miR-33-5p, miR-132-3p, and miR-212-3p in primary hepatocytes from *Tcf7l2*^*f/f*^ (*n*=3) and *Alb-Cre;Tcf7l2*^*f/f*^ (*n*=3) mice fed an HCD for 16 weeks. (**e**) miR binding sites in the *Srebf1* 3′UTR are shown. Luciferase reporter assay data showing effects of miR-33-5p, miR-132-3p and miR-212-3p mimics on *Srebf1* 3′UTR activity in the HEK293T cell line are also presented (*n*=3 replicates). Data are representative of *n*=3 independent experiments. (**f**) *Tcf7l2* WT (single-guide negative control [sgNC]; *Tcf7l2*^+/+^) and *Tcf7l2* KO (sg*Tcf7l2*; *Tcf7l2*^−/−^) AML12 cell lines were generated using the CRISPR-Cas9 system. qPCR analysis showing mRNA levels of *Srebf1c*, *Fasn* and *Acaca* in sgNC and sg*Tcf7l2* AML12 cells transfected with miR mimics (*n*=3 replicates). Data are representative of *n*=3 independent experiments. (**g**) *Tcf7l2* WT (sgNC; *Tcf7l2*^+/+^) and *Tcf7l2* KO (sg*Tcf7l2*; *Tcf7l2*^−/−^) AML12 cell lines were generated using the CRISPR-Cas9 system. sgNC and sg*Tcf7l2* AML12 cells were infected with Ad-*gfp*, Ad-miR-33-5p or Ad-miR-132/212-3p and treated with 3% (vol./vol.) ethanol in saline (control [Con]) (Ad-*gfp*, *n*=3 for both genotypes; Ad-miR-33-5p, *n*=3 for both genotypes; Ad-miR-132/212-3p, *n*=3 for both genotypes) or were infected with Ad-*gfp*, Ad-miR-33-5p or Ad-miR-132/212-3p and treated with 37 kBq of [^14^C]glucose and 10 nmol/l insulin (Ad-*gfp*; *n*=5 for both genotypes; Ad-miR-33-5p, *n*=6 for sgNC and *n*=5 for sg*Tcf7l2*; Ad-miR-132/212-3p; *n*=5 for sgNC and *n*=6 for sg*Tcf7l2*) for 48 h. Incorporation of [^14^C]glucose into TG was measured to determine DNL rate. (**h**) *Tcf7l2* WT (sgNC; *Tcf7l2*^+/+^) and *Tcf7l2* KO (sg*Tcf7l2*; *Tcf7l2*^−/−^) AML12 cell lines were generated using the CRISPR-Cas9 system. Intracellular TG levels were measured in sgNC and sg*Tcf7l2* AML12 cells transfected with miR-33-5p mimics (*n*=4 per group). (**i**) Schematic diagram showing the location of the putative TBE site. Also presented is luciferase reporter assay data showing the effects of TCF7L2 expression on miR-33-5p promoter activity in the HEPG2 cell line (*n*=3 replicates). Data are representative of *n*=3 independent experiments. (**j**) Schematic diagram showing the location of the putative TBE and primers for ChIP assay targeting the miR-33-5p promoter. The bar graph presents ChIP assay data showing occupancy of TCF7L2 over the miR-33-5p promoter in the murine hepatocyte cell line AML12 (*n*=3 replicates). Data are representative of *n*=3 independent experiments. A diagram showing the proposed mechanism of TCF7L2-associated miR-33-5p regulation is also shown. IP, immunoprecipitation. Key in (**a**) also applies to (**c**). Data are presented as mean±SD. *p<0.05, **p<0.01, ***p<0.001, analysed by *t* test

Under refeeding conditions, hepatic *Tcf7l2* deficiency significantly upregulated *Srebf1c* mRNA levels (Fig. 7a). However, TCF7L2 did not affect *Srebf1c* promoter activity induced by insulin, SREBP1c (autoregulation) or the LXR agonist T0901317 (Fig. 7b). Therefore, we investigated whether TCF7L2 regulates miRs that can trigger *Srebf1c* mRNA decay during the development of hepatic steatosis [39–41]. We found that hepatic *Tcf7l2* deficiency decreased the hepatic expression of miR-33-5p, miR-132-3p and miR-212-3p under refeeding conditions (Fig. 7c). We noted that miR-33-5p showed apparently similar expression patterns to *Tcf7l2* during periods of HCD feeding and was markedly decreased in the liver and primary hepatocytes of *Alb-Cre;Tcf7l2*^*f/f*^ mice fed a chronic HCD (Fig. 7d and ESM Fig. 10 [comparative HCD feeding data for TCF7L2 are shown in Fig. 3c and ESM Fig. 5d,e]). miR-33-5p also had a potent inhibitory effect on the activity of the murine *Srebf1* 3′UTR, as determined by luciferase reporter assay (Fig. 7e). Similarly, compared with the other miRs investigated, miR-33-5p significantly reduced basal *Fasn* and *Acaca* expression and *Tcf7l2* deficiency-induced expression of *Srebf1c*, *Fasn* and *Acaca* (Fig. 7f). Consistent with this, miR-33-5p significantly reduced *Tcf7l2* deficiency-induced DNL and hepatic TG content (Fig. 7g,h). To further elucidate the relationship between TCF7L2 and miR-33-5p, we tested whether TCF7L2 could transcriptionally regulate miR-33-5p. Careful investigation of promoter sequences showed that the putative TCF-binding element (TBE) site was localised at −1249 to −1243 from the transcriptional start site of the miR-33-5p promoter (Fig. 7i). Indeed, expression of hepatic TCF7L2 enhanced activity of the miR-33-5p promoter, that included a putative TBE site (−1412 to +16), but did not affect the activity of the miR-33-5p promoter without a putative TBE site (−852 to +16; Fig. 7i). TCF7L2 specifically occupied the miR-33-5p promoter region containing the putative TBE site (−1249 to −1243; Fig. 7j).

### Restoration of hepatic Tcf7l2 alleviates hepatic steatosis induced by acute and chronic HCD

**Fig. 8:**
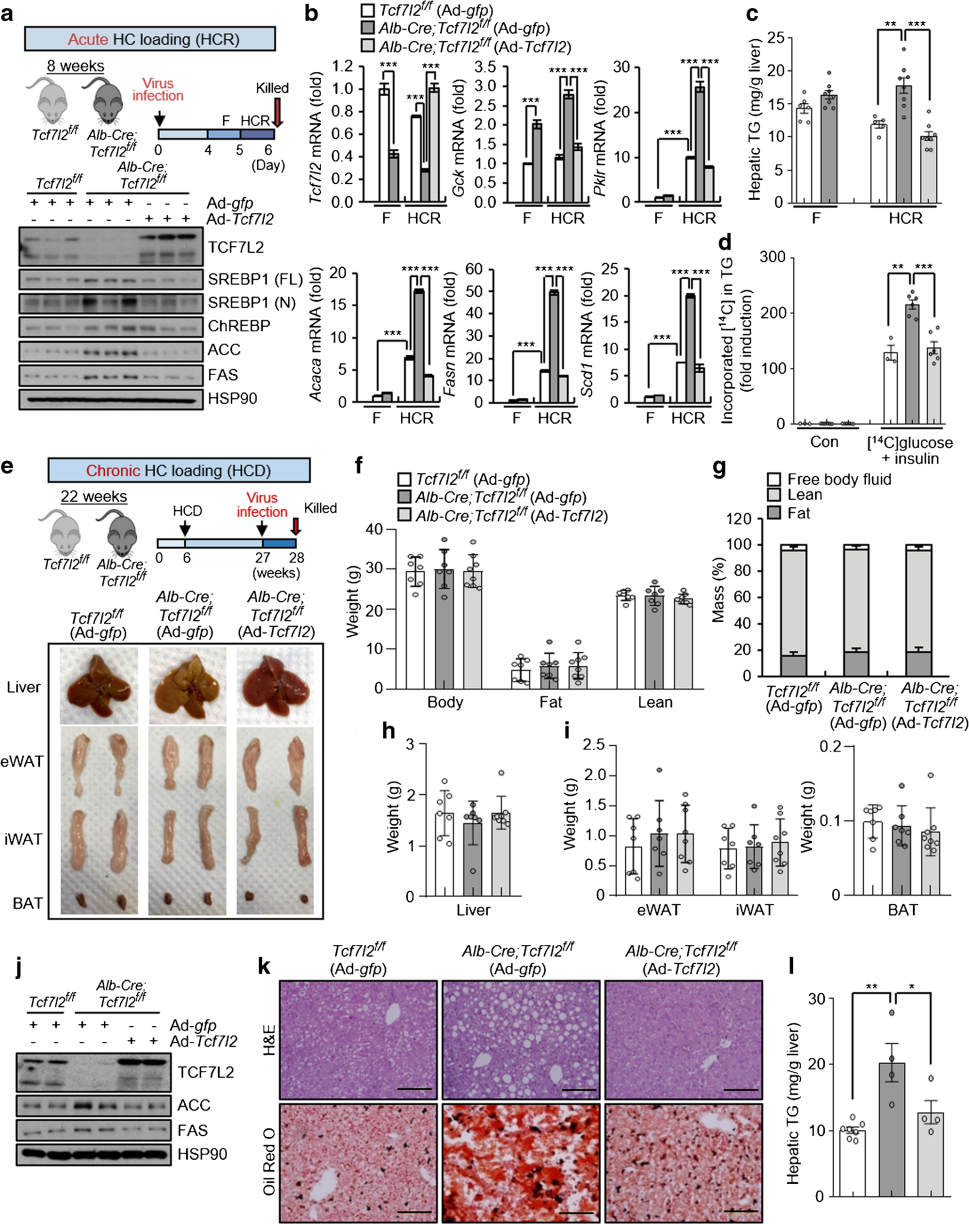
Effects of restoring hepatic *Tcf7l2* expression on liver steatosis induced by acute and chronic HCD feeding in *Alb-Cre;Tcf7l2*^*f/f*^ mice. (**a**–**c**) Eight-week-old *Tcf7l2*^*f/f*^ (*n*=6) and *Alb-Cre;Tcf7l2*^*f/f*^ (*n*=8) mice infected with Ad-*gfp* were fasted (F) for 24 h. Subsequently, *Tcf7l2*^*f/f*^ (*n*=5) and *Alb-Cre;Tcf7l2*^*f/f*^ mice infected with Ad-*gfp* (*n*=8) or Ad-*Tcf7l2* (*n*=8) were subjected to HCR for 24 h. A schematic diagram showing the experimental design for generation of the acute high-carbohydrate loading mouse model is shown alongside a representative western blot showing TCF7L2, SREBP1 (full length [FL] and nuclear [N] forms), ChREBP, ACC and FAS protein levels in mouse liver (**a**). Quantitative PCR (qPCR) analysis showing expression levels of *Tcf7l2* and glycolytic (*Gck* and *Pklr*) and lipogenic (*Acaca*, *Fasn* and *Scd1*) genes in mouse liver (**b**). Hepatic TG levels in mice (**c**). (**d**) DNL rate in primary hepatocytes isolated from *Tcf7l2*^*f/f*^ and *Alb-Cre;Tcf7l2*^*f/f*^ mice*.* Cells were infected with Ad-*gfp* or Ad-*Tcf7l2* and treated with [^14^C]glucose and 10 nmol/l insulin or 3% (vol./vol.) ethanol in saline (control [Con]) for 48 h. (**e**–**i**) Six-week-old *Tcf7l2*^*f/f*^ (*n*=7) and *Alb-Cre;Tcf7l2*^*f/f*^ (*n*=15) mice were fed an HCD for 22 weeks. At 21 weeks of HCD, *Tcf7l2*^*f/f*^ mice were infected with Ad-*gfp* (*n*=7) and *Alb-Cre;Tcf7l2*^*f/f*^ mice were infected with Ad-*gfp* (*n*=7) or Ad-*Tcf7l2* (*n*=8). A schematic diagram showing the experimental design for generation of the chronic high-carbohydrate loading mouse model is shown alongside representative images of livers, epididymal white adipose tissues (eWAT), inguinal white adipose tissues (iWAT) and brown adipose tissues (BAT) from mice (**e**). Body, fat and lean weights (**f**). Percentages of fat mass, lean mass and free body fluid (**g**). Liver weight (**h**). Weights of eWAT, iWAT and BAT (**i**). (**j**-**l**) Six-week-old *Tcf7l2*^*f/f*^ (*n*=7) and *Alb-Cre;Tcf7l2*^*f/f*^ (*n*=8) mice were fed an HCD for 22 weeks. At 21 weeks of HCD, *Tcf7l2*^*f/f*^ mice were infected with Ad-*gfp* (*n*=7) and *Alb-Cre;Tcf7l2*^*f/f*^ mice were infected with Ad-*gfp* (*n*=4) or Ad-*Tcf7l2* (*n*=4). A representative western blot showing TCF7L2, ACC and FAS protein levels in mouse liver (**j**; quantification data are presented in ESM Fig. 11d). Representative H&E and Oil Red O staining of liver sections (×20 magnification; scale bars, 200 μm; **k**). Hepatic TG levels (**l**). HSP90, heat shock protein 90. Key in (**b**) also applies to (**c**), (**d**), (**h**), (**i**) and (**l**). Data in (**b–d**), (**f–i**) and (**l**) are presented as mean±SEM. *p<0.05, **p<0.01, ***p<0.001, assessed by one-way ANOVA with Tukey’s post hoc test

To further investigate the physiological role of hepatic *Tcf7l2* in high-carbohydrate-induced fatty liver, we restored hepatic *Tcf7l2* expression in *Alb-Cre;Tcf7l2*^*f/f*^ mice under acute and chronic HCD conditions. The restoration of hepatic *Tcf7l2* expression in mice subjected to HCR reduced the expression of glycolytic and lipogenic factors to normal levels (Fig. 8a,b). This resulted in the restoration of hepatic DNL and TG levels (Fig. 8c,d). Under chronic HCD conditions, hepatic *Tcf7l2* restoration did not cause changes in body weight, adiposity or body composition, and it did not affect the weights of the liver, epididymal white adipose tissue, inguinal white adipose tissue or brown adipose tissues (Fig. 8e–i). Plasma TG and NEFA levels also remained unchanged (ESM Fig. 11a–c). However, similar to the model of acute HCD, hepatic *Tcf7l2* restoration significantly recovered protein levels of key lipogenic enzymes (ACC and FAS) and mRNA levels of glycolytic *Gck* and lipogenic *Fasn*, and restored chronic HCD-induced hepatic lipid droplet formation and hepatic TG levels as shown by Oil Red O staining of liver sections (Fig. 8j–l and ESM Fig. 11d,e). Indeed, adenovirus-mediated expression of hepatic *Tcf7l2* in wild-type C57BL/6N mice fed an HCD for 22 weeks decreased chronic HCD-induced hepatic lipid droplets and TG content, concomitant with reductions in DNL-associated genes, without changes in body weight (ESM Fig. 11f–j).

### *Alb-Cre;Tcf7l2*^*f/f*^ mice fed an HCD develop a NASH phenotype

To further emphasise the pathological role of hepatic *Tcf7l2* deficiency in the liver, we explored whether the absence of hepatic *Tcf7l2* exacerbated NAFLD into more severe forms. Mice lacking hepatic *Tcf7l2* displayed simple liver steatosis at 16 weeks of HCD feeding and progressive fatty liver at 34 weeks of a long-term HCD (ESM Fig. 12a). In detail, *Alb-Cre;Tcf7l2*^*f/f*^ mice fed an HCD for 34 weeks exhibited steatosis and lobular inflammation (F4/80-positive area) in the liver (ESM Fig. 12b,c). Although the data are not shown, we observed some hepatocellular ballooning with Mallory–Denk bodies in the liver. *Alb-Cre;Tcf7l2*^*f/f*^ mice had elevated NAS, which is the sum of scores for steatosis, lobular inflammation and ballooning (ESM Fig. 12d). Additionally, liver fibrosis, as determined by the proportion of the Sirius red-stained area, was significantly increased upon hepatic *Tcf7l2* deficiency (ESM Fig. 12e). Inflammation and fibrosis in the liver of *Alb-Cre;Tcf7l2*^*f/f*^ mice was further confirmed by analysis of mRNA and protein levels (ESM Fig. 12f,g).

## Discussion

This study aimed to explore how changes in TCF7L2 expression in the liver affect NAFLD development. We found that *Tcf7l2* expression was reduced in the liver of both human and diet-induced mouse models of NAFLD/NASH. To gain new insights into the relationship between reduced hepatic *Tcf7l2* expression and NAFLD development, we generated a mouse model with hepatocyte-specific *Tcf7l2* deficiency. Several previous studies have attempted to elucidate the role of TCF7L2 in hepatic lipid metabolism using mouse models [42–44]; however, contrasting results have been obtained. Whole-body *Tcf7l2* deficiency contributed to a reduction in hepatic lipid content, whereas *Tcf7l2*DN caused an increase in hepatic lipid content [38–40]. Despite advanced tools for in vitro-derived 3D cell culture systems, including liver organoids that can replace animal models, there are still several limitations to reproducing the in vivo process of diet-induced NAFLD, which is influenced by varying and complex factors. Therefore, we used a mouse model capable of inducing NAFLD in vivo.

Hepatic *Tcf7l2* deficiency exacerbated liver steatosis via carbohydrate dependency owing to cell-autonomous increases in DNL. During the DNL process, glucose 6-phosphate can be synthesised by or broken down into glycogen [45], and acetyl-CoA can be converted into or back from ketone bodies [44, 46]. However, hepatic glycogen and β-OH levels did not change in the *Alb-Cre;Tcf7l2*^*f/f*^ mice used in our study, as compared with wild-type mice, indicating that the glycolytic/lipogenic pathway may be the key signalling pathway for liver TG accumulation upon *Tcf7l2* deletion without interference from other signalling pathways. As a strategy to avoid the potentially redundant or compensatory effects of other TCF members following *Tcf7l2* deficiency [8, 44, 47], we performed *Tcf7l2*DN-mediated functional knockdown studies on hepatic DNL. Adenovirus-mediated *Tcf7l2*DN expression promoted hepatic DNL in a cell-autonomous manner, similar to that shown in *Tcf7l2*^*f/f*^-derived primary hepatocytes infected with adenovirus expressing *Cre.*

Excessive exogenous fatty acid uptake is one of the leading contributors to intrahepatic TG accumulation in the pathogenesis of NAFLD. However, hepatic *Tcf7l2* deficiency did not increase HFD-induced fat accumulation in the liver. Fatty acids typically inhibit glucose uptake [32, 33]. In the fatty acid/glucose axis, hepatic *Tcf7l2* deficiency alleviated inhibition of glucose uptake by fatty acids, resulting in relatively increased glucose uptake compared with controls. Further, this promoted glucose-sensing under HFD conditions, as confirmed by the expression of *pklr*, a glucose-sensing marker. This might contribute to balanced basal induction of hepatic lipogenesis under HFD conditions. This might be the reason why hepatic *Tcf7l2* KO did not alter hepatic lipid content despite the partially decreased fatty acid uptake observed under HFD conditions. Importantly, these data suggest that excessive fatty acid uptake is not the mechanism driving NAFLD following hepatic *Tcf7l2* loss.

Hepatic *Tcf7l2* expression increased under conditions that stimulate DNL by refeeding with both a standard chow diet or an HCD. Increased hepatic *Tcf7l2* expression suppressed hepatic DNL. However, excessively increased intrahepatic fatty acid owing to chronic HCD feeding eventually decreased hepatic TCF7L2 expression and activity. Therefore, the early induction of hepatic TCF7L2 expression by dietary carbohydrates may play an important role in maintaining hepatic lipid homeostasis, although reduced hepatic TCF7L2 expression may indicate a transition to a pathological state.

Chronic HCD feeding reduced ChREBP protein levels, whereas hepatic *Tcf7l2* deficiency maintained these. TCF7L2 modulated ChREBP protein content through *O*-GlcNAcylation, resulting in the regulation of ChREBP transcriptional activity as confirmed by treatment with the OGT inhibitor OSMI-1. ChREBP interacts with OGT and induces *O*-GlcNAcylation in liver cells [38]. However, TCF7L2 did not transcriptionally regulate OGT, as confirmed by reporter gene assay (data not shown). Therefore, TCF7L2 may be involved in the binding of OGT to ChREBP for regulation of *O*-GlcNAcylation. Host cell factor 1 (HCF-1) is a ChREBP-interacting DNL protein that recruits OGT to ChREBP, resulting in ChREBP *O*-GlcNAcylation and activation. TCF7L2 may directly target HCF-1 gene transcription [48].

miR-33-5p plays an important role in *Srebf1* mRNA decay during the development of hepatic steatosis [39, 49]. In our study, hepatic miR-33-5p displayed expression patterns similar to *Tcf7l2* during HCD feeding and was transcriptionally targeted by TCF7L2. Indeed, miR-33-5p reduced *Tcf7l2* deficiency-induced expression and function of *Srebf1c*. On the other hand, although miR-132-3p and miR-212-3p (other miRs that can cause *Srebf1c* mRNA decay [40, 41]) were decreased in primary hepatocytes and the liver of hepatic *Tcf7l2-*deficient mice, and reduced *Srebf1* 3′UTR luciferase activity, they did not inhibit the expression and function of *Srebf1c*. These data suggest that miR-33-5p is an important factor for regulating the expression and function of *Srebf1c* in hepatic *Tcf7l2* deficiency-induced liver steatosis.

### Conclusion

Although this study did not model the *Tcf7l2* SNP associated with the risk of type 2 diabetes, based on its potential to utilise the excess glucose pool more efficiently, we suggest that TCF7L2 may be a promising regulator of NAFLD associated with dietary carbohydrates and diabetes. Pathologically, hepatic *Tcf7l2* deficiency-induced fatty liver progressed to NASH, a severe form of NAFLD, demonstrating the potent effect of TCF7L2 on hepatic DNL. Therefore, *Alb-Cre;Tcf7l2*^*f/f*^ mice could be a useful mouse model to investigate NAFLD development and progression under HCD conditions.

## Supplementary Information

ESM (PDF 1.66 MB)

## Data Availability

RNA-sequencing data have been deposited into the NCBI GEO under the accession number GSE162449 (www.ncbi.nlm.nih.gov/geo/query/acc.cgi?acc=GSE162449). Data that support the findings of this study are available from the lead contact upon reasonable request.

## Abbreviations

ACC: Acetyl-CoA carboxylase
Ad-*gfp*: Adenovirus expressing *gfp*
AML12: Alpha-mouse-liver-12 (cell line)
AMPK: AMP-activated protein kinase
ChIP: Chromatin immunoprecipitation
ChoRE: Carbohydrate response element
ChREBP: Carbohydrate response element binding protein
CPT1α: Carnitine palmitoyltransferase 1α
2-DG: 2-Deoxyglucose
DN: Dominant negative
DNL: De novo lipogenesis
FAS: Fatty acid synthase
gRNA: Guide RNA
GSEA: Gene Set Enrichment Analysis
HCD: High-carbohydrate diet
HCF-1: Host cell factor 1
HCR: High-carbohydrate refeeding
HFD: High-fat diet
KO: Knockout
L-PK: L-type pyruvate kinase
LXR: Liver X receptor
miR: miRNA
MLX: Max-like protein X
NAFLD: Non-alcoholic fatty liver disease
NAS: Non-alcoholic fatty liver disease Activity Score
NASH: Non-alcoholic steatohepatitis
OCR: Oxygen consumption rate
OGT: *O*-GlcNAc transferase
β-OH: β-hydroxybutyrate
RC: Read count
RER: Respiratory exchange ratio
SRE: Sterol regulatory element
SREBP1: Sterol regulatory element binding protein 1
TBE: TCF-binding element
TCF7L2: Transcription factor 7-like 2
TG: Triglyceride
WGA: Wheat germ agglutinin

## References

[1] Jin T (2016). Current understanding on role of the Wnt signalling pathway effector TCF7L2 in glucose homeostasis. Endocr Rev.

[2] Migliorini A, Lickert H (2015). Beyond association: a functional role for Tcf7l2 in β-cell development. Mol Metab.

[3] Shu L, Matveyenko AV, Kerr-Conte J, Cho JH, McIntosh CH, Maedler K (2009). Decreased TCF7L2 protein levels in type 2 diabetes mellitus correlate with downregulation of GIP- and GLP-1 receptors and impaired beta-cell function. Hum Mol Genet.

[4] Nguyen-Tu MS, Martinez-Sanchez A, Leclerc I, Rutter GA, da Silva Xavier G (2021). Adipocyte-specific deletion of Tcf7l2 induces dysregulated lipid metabolism and impairs glucose tolerance in mice. Diabetologia.

[5] Geoghegan G, Simcox J, Seldin MM (2019). Targeted deletion of Tcf7l2 in adipocytes promotes adipocyte hypertrophy and impaired glucose metabolism. Mol Metab.

[6] Ip W, Shao W, Chiang YT, Jin T (2012). The Wnt signalling pathway effector TCF7L2 is upregulated by insulin and represses hepatic gluconeogenesis. Am J Physiol Endocrinol Metab.

[7] Oh KJ, Park J, Kim SS, Oh H, Choi CS, Koo SH (2012). TCF7L2 modulates glucose homeostasis by regulating CREB- and FoxO1-dependent transcriptional pathway in liver. PLoS Genet.

[8] Ip W, Shao W, Song Z, Chen Z, Wheeler MB, Jin T (2015). Liver-specific expression of dominant-negative transcription factor 7-like 2 causes progressive impairment in glucose homeostasis. Diabetes.

[9] Florez JC, Jablonski KA, Bayley N (2006). TCF7L2 polymorphisms and progression to diabetes in the Diabetes Prevention Program. N Engl J Med.

[10] Grant SF, Thorleifsson G, Reynisdottir I (2006). Variant of transcription factor 7-like 2 (TCF7L2) gene confers risk of type 2 diabetes. Nat Genet.

[11] Del Bosque-Plata L, Martínez-Martínez E, Espinoza-Camacho MÁ, Gragnoli C (2021). The role of TCF7L2 in type 2 diabetes. Diabetes.

[12] Musso G, Gambino R, Pacini G, Pagano G, Durazzo M, Cassader M (2009). Transcription factor 7-like 2 polymorphism modulates glucose and lipid homeostasis, adipokine profile, and hepatocyte apoptosis in NASH. Hepatology.

[13] Bril F, Cusi K (2017). Management of nonalcoholic fatty liver disease in patients with type 2 diabetes: a call to action. Diabetes Care.

[14] Xia MF, Bian H, Gao X (2019). NAFLD and diabetes: two sides of the same coin? Rationale for gene-based personalized NAFLD treatment. Front Pharmacol.

[15] Kim H, Lee DS, An TH (2021). Metabolic spectrum of liver failure in type 2 diabetes and obesity: from NAFLD to NASH to HCC. Int J Mol Sci.

[16] Fabbrini E, Magkos F (2015). Hepatic steatosis as a marker of metabolic dysfunction. Nutrients.

[17] Diraison F, Moulin P, Beylot M (2003). Contribution of hepatic de novo lipogenesis and reesterification of plasma non esterified fatty acids to plasma triglyceride synthesis during non-alcoholic fatty liver disease. Diabetes Metab.

[18] Donnelly KL, Smith CI, Schwarzenberg SJ, Jessurun J, Boldt MD, Parks EJ (2005). Sources of fatty acids stored in liver and secreted via lipoproteins in patients with nonalcoholic fatty liver disease. J Clin Invest.

[19] Solinas G, Borén J, Dulloo AG (2015). De novo lipogenesis in metabolic homeostasis: more friend than foe?. Mol Metab.

[20] Kim SH, Kim G, Han DH (2017). Ezetimibe ameliorates steatohepatitis via AMP activated protein kinase-TFEB-mediated activation of autophagy and NLRP3 inflammasome inhibition. Autophagy.

[21] Kleiner DE, Brunt EM, Van Natta M (2005). Design and validation of a histological scoring system for nonalcoholic fatty liver disease. Hepatology.

[22] Jung E, Seong Y, Jeon B, Kwon YS, Song H (2018). MicroRNAs of miR-17-92 cluster increase gene expression by targeting mRNA-destabilization pathways. Biochim Biophys Acta Gene Regul Mech.

[23] Byun SK, An TH, Son MJ (2017). HDAC11 inhibits myoblast differentiation through repression of MyoD-dependent transcription. Mol Cells.

[24] Mishra AP, Siva AB, Gurunathan C, Komala Y, Lakshmi BJ (2020). Impaired liver regeneration and lipid homeostasis in CCl_4_ treated WDR13 deficient mice. Lab Anim Res.

[25] Liang W, Menke AL, Driessen A (2014). Establishment of a general NAFLD scoring system for rodent models and comparison to human liver pathology. PLoS One.

[26] Ran FA, Hsu PD, Wright J, Agarwala V, Scott DA, Zhang F (2013). Genome engineering using the CRISPR-Cas9 system. Nat Protoc.

[27] Lee DS, Choi H, Han BS (2016). c-Jun regulates adipocyte differentiation via the KLF15-mediated mode. Biochem Biophys Res Commun.

[28] Miyao M, Kotani H, Ishida T (2015). Pivotal role of liver sinusoidal endothelial cells in NAFLD/NASH progression. Lab Invest.

[29] Muramatsu-Kato K, Itoh H, Kohmura-Kobayashi Y (2015). Undernourishment in utero primes hepatic steatosis in adult mice offspring on an obesogenic diet; involvement of endoplasmic reticulum stress. Sci Rep.

[30] Pompili S, Vetuschi A, Gaudio E (2020). Long-term abuse of a high-carbohydrate diet is as harmful as a high-fat diet for development and progression of liver injury in a mouse model of NAFLD/NASH. Nutrition.

[31] Duarte JA, Carvalho F, Pearson M (2014). A high-fat diet suppresses de novo lipogenesis and desaturation but not elongation and triglyceride synthesis in mice. J Lipid Res.

[32] Boden G, Chen X, Ruiz J, White JV, Rossetti L (1994). Mechanisms of fatty acid-induced inhibition of glucose uptake. J Clin Invest.

[33] Homko CJ, Cheung P, Boden G (2003). Effects of free fatty acids on glucose uptake and utilization in healthy women. Diabetes.

[34] Turner N, Cooney GJ, Kraegen EW, Bruce CR (2014). Fatty acid metabolism, energy expenditure and insulin resistance in muscle. J Endocrinol.

[35] Linden AG, Li S, Choi HY (2018). Interplay between ChREBP and SREBP-1c coordinates postprandial glycolysis and lipogenesis in livers of mice. J Lipid Res.

[36] Wang Y, Viscarra J, Kim SJ, Sul HS (2015). Transcriptional regulation of hepatic lipogenesis. Nat Rev Mol Cell Biol.

[37] Abdul-Wahed A, Guilmeau S, Postic C (2017). Sweet sixteenth for ChREBP: established roles and future goals. Cell Metab.

[38] Guinez C, Filhoulaud G, Rayah-Benhamed F (2011). O-GlcNAcylation increases ChREBP protein content and transcriptional activity in liver. Diabetes.

[39] Horie T, Nishino T, Baba O (2013). MicroRNA-33 regulates sterol regulatory element-binding protein 1 expression in mice. Nat Commun.

[40] Hu Z, Shen WJ, Cortez Y (2013). Hormonal regulation of microRNA expression in steroid producing cells of the ovary, testis and adrenal gland. PLoS One.

[41] Li Y, Zhang J, He J, Zhou W, Xiang G, Xu R (2016). MicroRNA-132 cause apoptosis of glioma cells through blockade of the SREBP-1c metabolic pathway related to SIRT1. Biomed Pharmacother.

[42] Yang H, Li Q, Lee JH, Shu Y (2012). Reduction in Tcf7l2 expression decreases diabetic susceptibility in mice. Int J Biol Sci.

[43] Boj SF, van Es JH, Huch M (2012). Diabetes risk gene and Wnt effector Tcf7l2/TCF4 controls hepatic response to perinatal and adult metabolic demand. Cell.

[44] Tian L, Shao W, Ip W, Song Z, Badakhshi Y, Jin T (2019). The developmental Wnt signalling pathway effector β-catenin/TCF mediates hepatic functions of the sex hormone estradiol in regulating lipid metabolism. PLoS Biol.

[45] Hengist A, Koumanov F, Gonzalez JT (2019). Fructose and metabolic health: governed by hepatic glycogen status?. J Physiol.

[46] Wakil SJ, Abu-Elheiga LA (2009). Fatty acid metabolism: target for metabolic syndrome. J Lipid Res.

[47] Takamoto I, Kubota N, Nakaya K (2014). TCF7L2 in mouse pancreatic beta cells plays a crucial role in glucose homeostasis by regulating beta cell mass. Diabetologia.

[48] Lane EA, Choi DW, Garcia-Haro L, Levine ZG, Tedoldi M, Walker S (2019). HCF-1 regulates de novo lipogenesis through a nutrient-sensitive complex with ChREBP. Mol Cell.

[49] Pan JH, Cha H, Tang J (2021). The role of microRNA-33 as a key regulator in hepatic lipogenesis signalling and a potential serological biomarker for NAFLD with excessive dietary fructose consumption in C57BL/6N mice. Food Funct.

