# Supplementary material for: Tcf7l2 in hepatocytes regulates de novo lipogenesis in diet-induced non-alcoholic fatty liver disease in mice
Source: Diabetologia. 2023 Feb 10;66(5):931–54. doi: 10.1007/s00125-023-05878-8 (PMC10036287; doi:10.1007/s00125-023-05878-8)
Supplement: Supplementary file 1 — (PDF 1.66 MB) [file 125_2023_5878_MOESM1_ESM.pdf]

Electronic Supplementary Material (ESM)

Tcf7l2 in hepatocytes regulates de novo lipogenesis in diet-induced non-alcoholic fatty liver disease in mice

Da Som Lee[1,2], Tae Hyeon An[2,3], Hyunmi Kim[2,3], Eunsun Jung[4], Gyeonghun Kim[5], Seung Yeon Oh[6], Jun Seok Kim[7], Hye Jin Chun[8], Jaeeun Jung[9], Eun-Woo Lee[2,3], Baek-Soo Han[3,10], Dai Hoon Han[11], Yong-Ho Lee[8,12], Tae-Su Han[3,4], Keun Hur[13], Chul-Ho Lee[3,14], Dae-Soo Kim[9], Won Kon Kim[2,3], Jun Won Park[15], Seung-Hoi Koo[7], Je Kyung Seong[5,6], Sang Chul Lee[2,3], Hail Kim[1], Kwang-Hee Bae[2,3], Kyoung-Jin Oh[2,3]

Affiliations

[1]Graduate School of Medical Science and Engineering, Korea Advanced Institute of Science and Technology, Daejeon, Republic of Korea

[2]Metabolic Regulation Research Center, Korea Research Institute of Bioscience and Biotechnology (KRIBB), Daejeon, Republic of Korea

[3]Department of Functional Genomics, KRIBB School of Bioscience, University of Science and Technology (UST), Daejeon, Republic of Korea

[4]Biotherapeutics Translational Research Center, Korea Research Institute of Bioscience and Biotechnology (KRIBB), Daejeon, Republic of Korea

[5]College of Veterinary Medicine, Seoul National University, Seoul, Republic of Korea

[6]Korea Mouse Phenotyping Center (KMPC), Seoul National University, Seoul, Republic of Korea

[7]Division of Life Sciences, Korea University, Seoul, Republic of Korea

[8]Department of Systems Biology, Glycosylation Network Research Center, Yonsei University, Seoul, Republic of Korea

[9]Environmental Diseases Research Center, Korea Research Institute of Bioscience and Biotechnology (KRIBB), Daejeon, Republic of Korea

[10]Biodefense Research Center, Korea Research Institute of Bioscience and Biotechnology (KRIBB), Daejeon, Republic of Korea

[11]Department of Surgery, Yonsei University College of Medicine, Seoul, Republic of Korea

[12]Department of Internal Medicine, Yonsei University College of Medicine, Seoul, Republic of Korea

[13]Department of Biochemistry and Cell Biology, School of Medicine, Kyungpook National University, Daegu, Republic of Korea

[14]Laboratory Animal Resource Center, Korea Research Institute of Bioscience and Biotechnology (KRIBB), Daejeon, Republic of Korea

[15]Division of Biomedical Convergence, College of Biomedical Science, Kangwon National University, ChunCheon-si, Gangwon-do, Republic of Korea

Da Som Lee, Tae Hyeon An and Hyunmi Kim contributed equally to this study.

Corresponding authors:

Hail Kim

Email: hailkim@kaist.edu

Kwang-Hee Bae

Email: khbae@kribb.re.kr

Kyoung-Jin Oh

Email: kjoh80@kribb.re.kr

ESM Methods

Human liver tissue samples Human liver tissue samples in ESM Fig. 1a were collected from 32 individuals who underwent hepatectomy or cholecystectomy at the university-affiliated Severance Hospital, Yonsei University College of Medicine, Republic of Korea, between September 2014 and October 2019, as previously described [1]. The non-tumor regions were isolated and used for RNA analysis. Liver histology was assessed by an experienced pathologist and, according to diagnostic criteria [21], the samples were put into the following groups: (1) normal (n=13); (2) simple steatosis (NAFLD; n=10); and (3) NASH (n=9). The diagnostic criteria for NASH included the presence of steatosis, ballooning, lobular inflammation, and fibrosis without history of excessive alcohol intake (< 210 g/week for men and 140 g/week for women) and hepatitis B or C virus infection. All individuals gave informed consent, and the study protocol was approved by the Institutional Review Board at Severance Hospital (IRB No 4–2014–0674).

Animal experiments Seven-week-old male C57BL/6N mice were purchased from ORIENT BIO (ORIENT BIO, Republic of Korea). *Tcf7l2* heterozygous null mice (*Tcf7l2*$^{+/–}$ mice; *Tcf7l2*$^{tm2a(EUCOMM)Wtsi}$; the EUCOMM Consortium, www.mousephenotype.org/data/genes/MGI:1202879, accessed on 8 September 2022) were backcrossed with C57BL/6N mice at least five times. To generate a conditional ready (floxed) allele by excising the L1L2_Bact_P promoter-driven cassette composed of an FRT flanked lacz/neomycin cassette, the mice were crossed with C57BL/6N transgenic mouse lines expressing the FLP recombinase. The heterozygous floxed (*Tcf7l2*$^{f/+}$) mice were used to generate homozygous *Tcf7l2*$^{f/f}$ mice. To generate liver-specific *Tcf7l2* knockout (liver KO, *Alb-Cre;Tcf7l2*$^{f/f}$) mice, *Tcf7l2*$^{f/f}$

mice were crossed with *albumin* (*Alb*)-*Cre* mice to induce the liver-specific deletion of exon 5. Six-week-old male *Alb-Cre;Tcf7l2*$^{f/f}$ mice and their WT littermates were given either an HFD (catalogue no. D12492; Research Diet, USA) or HCD (catalogue no. TD.98090; Envigo, USA) for the indicated periods. For GTTs and ITTs, mice were fasted for 16 h or 6 h, respectively, and injected intraperitoneally with 1.5 g/kg of glucose or 1 U/kg of insulin. Blood glucose levels were measured in blood samples taken from the tail vein (LifeScan; One-Touch, USA). Oxygen consumption ($\dot{V}\mathrm{O}_2$), carbon dioxide production ($\dot{V}\mathrm{CO}_2$), respiratory exchange ratio (RER, $\dot{V}\mathrm{CO}_2/\dot{V}\mathrm{O}_2$), and energy expenditure were measured in mice at 10 weeks using the Oxylet Pro system (Panlab, Spain). To minimize the effects of subjective bias, we tried to allocate 10 or more animals to experimental group. However, the number of mice used in the experiment was dependent on the number of individuals born at that time. The phenotype that should be derived together was analysed by dividing the number of individuals in the same experimental set. The number of animals or samples in each experimental group was indicated in each figure legend. Refer to each figure legend.

Mice were placed and measured in an individual metabolic chamber with free access to food and water for 24 h. All mice were maintained on a 12 h light/dark cycle in temperature- and humidity-controlled conditions, and were allowed free access to food and water. All animal procedures were approved by the Institutional Animal Care and Use Committee of the Korea Research Institute of Bioscience & Biotechnology (KRIBB-AEC-21051), and were performed according to the guidelines for the Care and Use of Laboratory Animals published by the US National Institutes of Health.

Mouse primary hepatocyte culture Primary hepatocytes were isolated from 8-week-old male C57BL/6N mice by collagenase perfusion [3]. Cells were plated in 6-well

plates with medium 199 (catalogue no. M4530; Sigma-Aldrich) supplemented with 10% (vol./vol.) fetal bovine serum (FBS), 1% (vol./vol.) antibiotics, and 10 nmol/l dexamethasone for 6 h. After attachment, cells were infected with adenovirus for 24 h (for adenovirus expressing *gfp*, *Tcf7l2*, and *Cre*). Subsequently, the cells were maintained in medium 199 without 10% FBS, and were treated with 25 mmol/l glucose (catalogue no. G7021; Sigma-Aldrich) and/or 100 nmol/l insulin (catalogue no. I6634; Sigma-Aldrich) for 48 h, 250 µmol/l palmitic acid (catalogue no. P9767; Sigma-Aldrich) for 24 h, 300 µmol/l AICAR (catalogue no. A611700; Toronto Research Chemicals, Canada) for 1 h, or 1 µmol/l T0901317 (catalogue no. T2320; Sigma-Aldrich) for 24 h.

Quantitative PCR Total RNA from human liver tissues, mouse tissues or mouse primary hepatocytes was extracted using the easy-spin Total RNA Extraction Kit (catalogue no. 17221; iNtRON Biotechnology, Republic of Korea). Complementary DNA (cDNA) was transcribed from 2 µg of total RNA using M-MLV Reverse Transcriptase (catalogue no. M1705; Promega, USA). Quantitative PCR (qPCR) was performed using the SYBR green PCR kit in a C1000 Touch Thermal Cycler (Bio-Rad Laboratories, USA). All data were normalised to the expression of ribosomal *L32*. Primer sequences are listed in ESM Table 1. MicroRNA (miRNA) expression was analysed as previously described [4]. Briefly, 0.5 ug of total RNA was polyadenylated and converted into cDNA using the PrimeScript RT reagent kit (catalogue no. RR037; TaKaRa, Japan). For quantitation of miRNAs, cDNA was amplified with miRNA primers and *A1S* primer. Primer sequences are listed in ESM Table 2.

Western blotting Western blot analysis of extracts from mouse livers and whole-cells was performed as previously described [5]. Proteins were separated by

electrophoresis on SDS–polyacrylamide gels and transferred to polyvinylidene difluoride (PVDF) membranes (catalogue no. 1620177; Bio-Rad Laboratories, USA). And then the membranes were blocked with 5% (wt/vol.) skim milk diluted in TRIS-buffered saline supplemented with Tween-20 (25 mmol/l Tris-HCl, 150 mmol/l NaCl and 0.1% [vol./vol.] Tween-20) and probed with primary antibodies diluted in TRIS-buffered saline with 0.1% (vol./vol.) Tween-20. Antibodies for TCF7L2, protein kinase B (AKT), Phospho-AKT, glycogen synthase kinase-3β (GSK3β), Phospho-GSK3β, FAS, ACC, AMP-activated protein kinase (AMPK), Phospho-AMPK were obtained from Cell Signaling Technology. Antibodies for HSP90, PKLR, HA-probe and ADGRE1 were from Santa Cruz (USA), antibody for SREBP1 was from BD Bioscience (USA), antibody for ChREBP was from Novus (USA), antibody for monoclonal ANTI-FLAG M2 was from Sigma, antibody for Mono-and polyubiquitinylated conjugates monoclonal antibody (FK2) was from Enzo Life Sciences (USA) and antibody for ACTA2 was from abcam. The specific signals were amplified by horseradish peroxidase-conjugated secondary antibody (Santa Cruz, USA) diluted 1:3000 in TRIS-buffered saline with 0.1% (vol./vol.) Tween-20 supplemented with 5% (wt/vol.) skim milk. Primary and secondary antibodies were used according to manufacturer's instructions and are listed in ESM Table 3.

Metabolite measurements Plasma insulin and IGF-1 levels were measured with a Mouse Insulin ELISA Kit (catalogue no. 80-INSMSE01; ALPCO, USA) and Mouse IGF1 ELISA Kit (catalogue no. EMIGF1; Thermo Fisher Scientific), respectively. Total lipids from mouse livers or mouse primary hepatocytes were extracted using the Folch method as described previously [3]. Briefly, 50 mg of frozen liver tissues were homogenized in with chloroform-methanol (2:1) mixture. Lipids were extracted using

sequential extraction with chloroform : methanol : 0.9% (wt/vol.) NaCl solution (5:5:2). A portion of organic phase was evaporated for 30 min or 1 h and dissolved in DMSO solution. Hepatic triglyceride (TG), cellular TG, plasma TG, and NEFA levels were measured with colorimetric assay kits (TG: catalogue no. 461-09092; NEFA: catalogue no. 438-91691; Wako, Japan). Hepatic glycogen was measured using an EnzyChrom Glycogen Assay Kit (catalogue no. E2GN-100; BioAssay Systems, USA). Hepatic β-hydroxybutyrate was measured with a β-hydroxybutyrate fluorometric assay kit (catalogue no. 700740; Cayman Chemical, USA).

Immunofluorescence staining Mouse pancreas tissues in ESM Fig. 1e and ESM Fig. 2d were fixed with 4% (wt/vol.) paraformaldehyde overnight and subsequently processed for paraffin sections. The embedded pancreas sections were cut into 4 μm. For antigen retrieval, slides were boiled in Sodium Citrate Buffer (10 mmol/l Sodium Citrate, 0.1% (vol./vol.) Tween 20, pH 6.0) at 95°C for 20 min. The sections were stained with anti-insulin (catalogue no. 53-9769-82; Thermo Fisher Scientific) and anti-glucagon (catalogue no. ab92517; Abcam, USA) antibodies overnight at 4°C. After washing three times with PBS, sections were incubated with DAPI (catalogue no. H-1800; Vector Laboratories, USA) and secondary antibodies (catalogue no. A21442; Invitrogen, USA) at room temperature for 1 h. Finally, the fluorescence images were taken using an OLYMPUS IX73 microscope (Olympus, Japan). Insulin-positive areas (beta cells), glucagon-positive areas (alpha cells) and pancreatic areas were quantified using Image J software (v 1.50i; https://imagej.nih.gov/ij/download.html). Antibody information is listed in ESM Table 4.

Histological analysis Liver tissues were fixed with 4% (wt/vol.) paraformaldehyde. For Oil Red O staining (catalogue no. O0625; Sigma-Aldrich, UK), the fixed tissues were snap-frozen in isopentane that had been precooled in liquid nitrogen [6]. The tissues were sliced into 10-μm-thick cryo-sections, which were stained with Oil Red O. For H&E staining, the tissues were embedded in paraffin, cut into sections, and stained with H&E. Liver fibrosis was detected with Sirius red staining (catalogue no. ab150681; Abcam, USA) according to the manufacturer's instructions. NAFLD Activity Score (NAS) and fibrosis stage were calculated as the sum of the scores for steatosis (0-3), lobular inflammation (0-3), hepatocyte ballooning (0-2), as assessed by H&E staining, and fibrosis (0-4) assessed by Sirius red staining, as commonly used in preclinical animal models and in patients [2, 7]. Histological samples were blinded using numbers and the slides analysed by an experienced pathologist.

Fatty acid uptake assay Mouse primary hepatocytes isolated from *Tcf7l2*$^{f/f}$ or C57BL/6N mice were transfected with the indicated plasmid DNA vectors using Lipofectamine 3000 transfection reagent (catalogue no. L3000015; Invitrogen, USA). Fatty acid uptake in mouse primary hepatocytes was measured with the Fatty Acid Uptake Assay Kit (catalogue no. K408-100; Biovision, USA) according to the manufacturer's instructions. Fluorescent fatty acid probes (green) were detected by a fluorescence microscope (DM IL LED FLUO; Leica Microsystems, Germany) and fluorescence intensity was quantified using Image J software (v 1.50i).

Glucose uptake assay Mouse primary hepatocytes isolated from *Tcf7l2*$^{f/f}$ mice were infected with adenoviruses expressing *gfp* only and *Cre* and treated with 1 nmol/l insulin for 30 min. 2-Deoxyglucose (2DG) levels in mouse primary hepatocytes were

measured using the 2-DG Uptake Measurement Kit (catalogue no. CSR-OKP-PMG-K01; Cosmo Bio, Japan) according to the manufacturer's instructions.

Analysis of oxygen consumption rate via Seahorse assay Fatty acid oxidation in mouse primary hepatocytes was assessed by analysing oxygen consumption rate (OCR). XF24 cell culture microplates (catalogue no. 100777004; Agilent Technologies, USA) were coated with collagen (catalogue no. 11179179001; Roche, Germany). Mouse primary hepatocytes were seeded on collagen-coated XF24 cell culture microplates 72 h before the experiment. The cells were maintained in a glucose-free culture medium. After the addition of BSA or 250 μmol/l palmitate and/or 100 μmol/l etomoxir (catalogue no. E1905; Sigma-Aldrich), cells were treated with 2.5 μmol/l oligomycin (catalogue no. O4876; Sigma-Aldrich), 10 μmol/l fluoro-carbonylcyanide phenylhydrazone (FCCP) (catalogue no. C2920; Sigma-Aldrich), and 2 μmol/l rotenone (catalogue no. R8875; Sigma-Aldrich) plus 5 μmol/l antimycin A (catalogue no. A8674; Sigma-Aldrich). The OCRs were determined with an XF24 extracellular flux analyzer (Seahorse Bioscience, USA) and normalised to the amount of protein in each sample.

DNL measurements For *in vitro* DNL analysis, mouse primary hepatocytes were treated with 37 kBq of ^{14}C-labelled glucose (catalogue no. NEC042; PerkinElmer, USA) and 10 nmol/l insulin for 48 h. Lipids were extracted from cells with chloroform. ^{14}C radioactivity was measured using the Tri-Carb 2910 TR liquid scintillation analyser (PerkinElmer, USA). For in vivo DNL analysis, mice were fasted for 24 h, then refed an HCD for 12 h. Subsequently, they were intraperitoneally injected with ^{14}C-labelled sodium acetate (555 kBq/mouse; catalogue no. NEC553; PerkinElmer) or 3% (vol./vol.)

ethanol in saline (Con) and euthanised 1 h post injection. Lipids were extracted using the Folch method [3] and incorporation rates of [1-^{14}C] acetic acid into lipids were measured using the Tri-Carb 2910 TR liquid scintillation analyser.

mRNA sequencing Total RNA from mouse livers was extracted using the easy-spin Total RNA Extraction Kit (iNtRON Biotechnology, Republic of Korea). Libraries were prepared from 2 ug of total RNA using the SMARTer Stranded RNA-Seq Kit (Clontech Laboratories, USA). The isolation of mRNA was performed using the Poly(A) RNA Selection Kit (LEXOGEN, USA). Quantification was performed using the library quantification kit using a StepOne Real-Time PCR System (Life Technologies, USA). High-throughput sequencing was performed as paired-end 100 sequencing using HiSeq 2500 (Illumina, USA). The alignment files were used for assembling transcripts, estimating their abundances and detecting differential expression of genes or isoforms using cufflinks. Differentially expressed gene was determined based on counts from unique and multiple alignments using coverage in Bedtools (v 2.25.0; https://bedtools.readthedocs.io/en/latest/index.html). The read count (RC) data were processed based on the quantile normalization method using EdgeR (v 3.20.1; https://bioconductor.org/packages/release/bioc/html/edgeR.html) within R (v 3.4.4; www.r-project.org) using Bioconductor (v 3.6; https://bioconductor.org/install/). The mRNA sequencing and analysis were performed by eBiogen (Republic of Korea). From the results obtained by setting the RC (log_2) value to 5 or more, genes with a log_2 fold change of greater than 1.5 (log_2 fold-change cut-off >1.5) in the KO vs wild-type comparisons were selected and were analysed using Gene Set Enrichment Analysis (GSEA; www.gsea-msigdb.org/gsea/msigdb/mouse/annotate.jsp, accessed on 1 November 2021), based on canonical pathways gene sets derived from the

Reactome pathway database (www.gsea-msigdb.org/gsea/msigdb/mouse/genesets.jsp?collection=CP:REACTOME, accessed on 1 November 2021).

Plasmids and recombinant adenoviruses Expression vectors used in this study, including mouse *Tcf7l2*, and adenovirus expressing TCF7L2 (Ad-*Tcf7l2*) and adenovirus expressing *gfp* (Ad-*gfp*) have been described previously [3]. Expression vectors for mouse *Tcf7l2*, nuclear *Srebf1c* and *Mlxipl*(α) were amplified by RT-PCR using liver RNA derived from C57BL/6N mice and inserted into pcDNA3-Flag or pcDNA3-HA expression vectors. The pGL4-6X *SRE*-luc (containing six copies of *SRE*) and pGL4-4X *ChoRE*-luc (containing four copies of *ChoRE*) constructs were a kind gift from Prof. S-H Koo (Korea University, Korea). Mouse *Fasn* (−2000/+200), *Pklr* (−191/+200), *Srebf1c* (−1195/+77), miR-33-5p (−852/+16), miR-33-5p (−1412/+16), *Mlxipl*(α) (−2538/+124) and *Mlxipl*(β) (−536/+352) promoter sequences were amplified by PCR using mouse genomic DNA and inserted into the pGL4-luciferase reporter vector. miRNA mimics (mmu-miR-33-5p, mmu-miR-132-3p, mmu-miR-212-3p and negative control) were purchased from GenePharma (Shanghai, China). Primer sequences are listed in ESM Table 5. In total, 50 μmol/l miRNA mimics were transfected into cells using Lipofectamine 3000 transfection reagent (Invitrogen) according to the manufacturer's instructions. Adenoviruses expressing *gfp* only, *Tcf7l2* and *Cre* have been described previously [3]. For animal experiments, the viruses were purified on a CsCl gradient, dialysed against PBS buffer containing 10% (vol./vol.) glycerol, and stored at −80°C.

Luciferase assay Luciferase assays were performed to determine the effects of TCF7L2 on the sterol regulatory element binding protein 1 (SREBP1)c, ChREBP/max-

like protein X (MLX) transcriptional activities and the miR-33-5p promoter activity in HEPG2 cells (catalogue no. HB-8065; ATCC, USA) and *Srebf1* 3′UTR promoter activity in HEK293T cells (catalogue no. CRL-3216; ATCC, USA). HEPG2 and HEK293T cells were tested to be mycoplasma-negative using BioMycoX Mycoplasma PCR Detection Kit (catalogue no. D-50; CellSafe, Republic of Korea). Human hepatoma cell line HEPG2 was transfected with 200 ng of luciferase construct, 50 ng of *β-galactosidase*, and 5–50 ng of TCF7L2, nuclear SREBP1c, ChREBP, or MLX expression vector using TransIT-LT1 Reagent (catalogue no. MIR-2304; Mirus Bio, USA). For SREBF1c 3'UTR luciferase assay, human kidney HEK293T was transfected with 200 ng of luciferase construct, 50 ng of *β-galactosidase*, and 50 nmol/l of miRNAs using Lipofectamine 2000 (catalogue no. 11668-019; Invitrogen, USA). Total cell lysates were prepared 48 h after transfection, and the promoter activity was measured using a luciferase reporter assay system (catalogue no. E1910; Promega, USA). The luciferase activity was normalised to β-galactosidase activity.

Protein stability assay HEPG2 cells were transfected with HA-tagged TCF7L2 and Flag-tagged ChREBPα for 48 h and treated with 10 μmol/l MG132 (catalogue no. M1157; AG Scientific, USA) or DMSO (catalogue no. D2650; Sigma-Aldrich) for 3 h.

Immunoprecipitation and wheat germ agglutinin purification Total lysates from liver tissues were centrifuged at 13,000 rpm for 10 min and the supernatant was extracted. For immunoprecipitation (IP), 3 mg of protein lysates were incubated with carbohydrate response element binding protein (ChREBP) antibody for 16 h and 30 μl of protein G plus A agarose beads (catalogue no. IP05; Millipore, USA) was added to each sample followed by rotation for an additional 2 h at 4°C. For wheat germ

agglutinin (WGA, a specific GlcNAc lectin) precipitation, 3 mg of protein lysates were incubated with 30 μl of WGA agarose beads (catalogue no. AL-1023; Vector Laboratories) for 16 h at 4°C. Beads were washed 3 times and eluted in 2× sample buffer without β-mercaptoethanol.

***Tcf7l2*-KO in alpha-mouse-liver-12 cell lines** To generate the *Tcf7l2*-knockout alpha-mouse-liver-12 (AML12) cell lines (catalogue no. CRL-2254; ATCC), we developed a clustered regularly interspaced short palindromic repeats (CRISPR)/Cas9 system for gene editing using standard methods [8]. The single-guide RNA (sgRNA) sequence was cloned into pSpCas9(BB)-2A-Puro(PX459), which was a gift from F. Zhang (Broad Institute of MIT and Harvard, Cambridge, USA). The target guide RNA (gRNA) sequence was 5′-AGCAATGAACACTTCACCCC-3′ (*Tcf7l2* exon 5; Genome Reference Consortium Mouse Reference 39 [GRCm39;]: 19:55,896,921–55,896,940; https://asia.ensembl.org/Mus_musculus/Info/Annotation). The vector expressing gRNA was transfected into AML12 cells, and then cells were selected using puromycin. Cells were tested to be mycoplasma-negative using BioMycoX Mycoplasma PCR Detection Kit (catalogue no. D-50; CellSafe, Republic of Korea). Genomic DNA was extracted from cells using the Exgene Tissue SV Kit (catalogue no. 104-152; GeneAll, Republic of Korea). Gene knockout was verified by PCR and western blot analysis. Cells were transfected with the pGL4-*Pklr* (−191/+200) promoter and then co-treated with 25 mmol/l glucose and 40 μmol/l OSMI-1 (catalogue no. ab235455; Abcam, UK) for 24 h.

Chromatin immunoprecipitation Cross-linking, nuclear isolation and chromatin immunoprecipitation (ChIP) assays were performed on AML12 cell line samples and

mouse primary hepatocyte samples, using previously reported methods [9]. Briefly, cells were treated with 1% (wt/vol.) formaldehyde for 20min to cross-link histones to DNA. Cross-linked cells were lysed with cell lysis buffer (0.1% [vol./vol.] NP40, 10 mmol/l KCl, 1.5 mmol/l $MgCl_2$, 1 mmol/l DTT, 25 mmol/l HEPES pH 7.8, and a protease inhibitor cocktail). Subsequently, the supernatants were removed, and the pellets were lysed with nuclear lysis buffer (1% [vol./vol.] Triton X-100, 140 mmol/l NaCl, 1 mmol/l EDTA, 0.1% [wt/vol.] Na-deoxycholate, 0.1% [wt/vol.] SDS, 50 mmol/l HEPES pH7.9, and a protease inhibitor cock-tail). The nuclear lysates were sonicated at 20% power output of the processor (catalogue no. VC505; Sonics & Materials, USA) and the supernatants were collected as chromatin samples. For each immunoprecipitation reaction, the supernatant proteins were immunoprecipitated with anti-TCF7L2 (catalogue no. 2569; Cell Signaling Technology, UK). We used anti-rabbit immunoglobulin G (IgG) as a negative control. The precipitated DNA fragments were analysed by PCR using primers against relevant mouse promoters. PCR primer pairs used were as follows: primer pairs containing the binding regions of TCF7L2 in the miR-33-5p promoter; Primer1: forward, 5′-TACTGTGCCACTGAGCCTC-3′ and reverse, 5′-CCTTGCTGTGGCATCTGCT-3′, Primer2: forward, 5′-TGTGTGTGTGAG TCATGGTAC-3′ and reverse, 5′-AGCACCCACATCTAACCAAC-3′.

Statistical analysis Statistical differences between two experimental groups were evaluated by the two-tailed unpaired Student's *t* test. One-way ANOVA with Tukey's multiple comparison test was performed using GraphPad Prism 8.0.1 (GraphPad Software, USA) when comparing three or more groups, as reported in the figure legends. Data are shown as mean±SD or mean±SEM. A *p* value <0.05 was considered statistically significant.

ESM References

1. Kim SH, Kim G, Han DH, et al (2017) Ezetimibe ameliorates steatohepatitis via AMP activated protein kinase-TFEB-mediated activation of autophagy and NLRP3 inflammasome inhibition. Autophagy 13:1767-1781. https://doi.org/ 10.1080 /15548627.2017.1356977
2. Kleiner DE, Brunt EM, Van Natta M et al (2005) Design and Validation of a Histological Scoring System for Nonalcoholic Fatty Liver Disease. Hepatology 41(6):1313-1321. https://doi.org/10.1002/hep.20701
3. Oh KJ, Park J, Kim SS, Oh H, Choi CS, Koo SH (2012) TCF7L2 modulates glucose homeostasis by regulating CREB- and FoxO1-dependent transcriptional pathway in liver. PLoS Genet 8(9):e1002986. https://doi.org/10. 1371/journal.pgen.1002986
4. Jung E, Seong Y, Jeon B, Kwon YS, Song H (2018) MicroRNAs of miR-17-92 cluster increase gene expression by targeting mRNA-destabilization pathways. Biochim Biophys Acta Gene Regul Mech 1861(7):603-612. https://doi.org/10.1016/j.bbagrm.2018.06.003
5. Byun SK, An TH, Son MJ et al (2017) HDAC11 Inhibits Myoblast Differentiation through Repression of MyoD-Dependent Transcription. Mol Cells 40(9):667-676. https://doi.org/10.14348/molcells.2017.0116
6. Mishra AP, Siva AB, Gurunathan C, Komala Y, Lakshmi BJ (2020) Impaired liver regeneration and lipid homeostasis in CCl_4 treated WDR13 deficient mice. Lab Anim Res 36(1):41. https://doi.org/10.1186/s42826-020-00076-8
7. Liang W, Menke AL, Driessen A et al (2014) Establishment of a general NAFLD scoring system for rodent models and comparison to human liver

pathology. PLoS One 9(12):e115922. https://doi.org/10.1371/journal.pone.0115922

8. Ran FA, Hsu PD, Wright J, Agarwala V, Scott DA, Zhang F (2013) Genome engineering using the CRISPR-Cas9 system. Nat Protoc 8(11):2281–2308. https://doi.org/10.1038/nprot.2013.143

9. Lee DS, Choi H, Han BS et al (2016) c-Jun regulates adipocyte differentiation via the KLF15-mediated mode. Biochem Biophys Res Commun 469(3): 552–558. https://doi.org/10.1016/j.bbrc.2015.12.035

ESM Tables

ESM Table 1. Primer sequences used for qPCR

Gene	Forward (5’- 3’)	Reverse (5 ’- 3’)
hTcf7l2	CATATGGTCCCACCACATCATACG	GAACTATGGAGTGAGCCGAC
hGapdh	AGCCAAAAGGGTCATCATCTC	GGACTGTGGTCATGAGTCCTTC
mTcf7l2	CTCACGCCTCTCATCACGTA	TCCTGTCGTGATTGGGTACA
mL32	GGCCTCTGGTGAAGCCCAAGATCG	CCTCTGGGTTTCCGCCAGTTTCGC
mSrebf1c	TACTTCTTGTGGCCCGTACC	TCAGGTCATGTTGGAAACCA
mMlxipl(α)	CGACACTCACCCACCTCTTC	TTGTTCAGCCGGATCTTGTC
mMlxipl(β)	TCTGCAGATCGCGTGGAG	CTTGTCCCGGCATAGCAAC
mSlc2a2	GCCTGTGTATGCAACCATTG	GAAGATGGCAGTCATGCTCA
mGck	GAAGACCTGAAGAAGGTGATGAGC	GTCTATGTCTTCGTGCCTTACAGG
mPklr	GAGTCGGAGGTGGAAATTGT	GTCCACCCACACTGTCTTTG
mFasn	TGGGTTCTAGCCAGCAGAGT	ACCACCAGAGACCGTTATGC
mAcaca	CTCCAGGACAGCACAGATCA	TGACTGCCGAAACATCTCTG
mAcly	GCCAGCGGGAGCACATC	CTTTGCAGGTGCCACTTCAT
mScd1	GTCAGGAGGGCAGGTTTC	GAGCGTGGACTTCGGTTC
mElovl6	TGCCATGTTCATCACCTTGT	TACTCAGCCTTCGTGGCTTT
mGpam	CAACACCATCCCCGACATC	GTGACCTTCGATTATGCGATCA
mDgat2	AGTGGCAATGCTATCATCATCGT	TCTTCTGGACCCATCGGCCCCAGGA
mPpara	AATGCAATTCGCTTTGGAAG	GGCCTTGACCTTGTTCATGT
mCpt1a	CCAGGCTACAGTGGGACATT	GAACTTGCCCATGTCCTTGT
mAcadm	AGGTTTCAAGATCGCAATGG	CTCCTTGGTGCTCCACTAGC
mAcox1	CAGGAAGAGCAAGGAAGTGG	CCTTTCTGGCTGATCCCATA
mPpargc1a	ATGTGTCGCCTTCTTGCTCT	ATCTACTGCCTGGGGACCTT
mPnpla2	TGTGGCCTCATTCCTCCTAC	TCGTGGATGTTGGTGGAGCT
mLipe	TCTATGCGCAGGAGTGTGTC	TTGACATCAGAGGGTGTGGA
mPlin2	GGAGTGGAAGAGAAGCATCG	TGGCATGTAGTCTGGAGCTG
mCd36	GCAAAGAAGGAAAGCCTGTG	CCAATGGTCCCAGTCTCATT
mSlc27a1	GGGAAGAGCCTCCTCAAGTT	TACCTGCTGTGCACCACAAT
mSlc27a2	TGCTGTCGGGAGAGCAAACT	AGAGTCCAACCTCACCTTTGGG
mSlc27a4	ATCAACACCAACCTTAGGCG	AACCCTTGTCTGGGTGACTG
mSlc27a5	GTGCTGACCTTGGACCAGGA	GCAGCCATGCCAACCTTACC
mFabp3	TGGCTAGCATGACCAAGCCT	GTTCCACTTCTGCACATGGA
mFabp4	GTCACCATCCGGTCAGAGAG	TCGACTTTCCATCCCACTTC
mFabp5	ACCTGGGAGAGAAGTTTGAT	GTTATCGTGCTCTCCTTCCC
mSrebf2	AAACCTCAGGCCAAGAAGAA	GAACTGCTGGAGAATGGTGA
mHmgcs2	GGCCTCACTTCTCTCTCACC	CTTGGACACTCGGAATGAAA
mHmgcr	TGGGCATGAACATGATCTCT	CTCGTCCTTCGATCCAATTT
mLdlr	TCCACATGGACCCACACACC	GGTCACCTCCGTGTCGAGAG
mTnf	GTCCCCAAAGGGATGAGAAG	CACTTGGTGGTTTGCTACGA
mIl1b	ACCCTGCAGCTGGAGAGTGT	TTGACTTCTATCTTGTTGAAGACAAACC
mIl6	GGGAAATCGTGGAAATGAGAAAA	TGGTAGCATCCATCATTTCTTTG
mItgam	ACACAGTGTGCTCCAGTATGA	GCCCAGGGATATGTTCACAGC
mCxcl1	ACTGCACCCAAACCGAAGTC	TGGGGACACCTTTTAGCATCTT
mAdgre1	CTTTGGCTATGGGCTTCCAGTC	GCAAGGAGGACAGAGTTTATCGTG

Gene	Forward (5’- 3’)	Reverse (5 ’- 3’)
mTimp1	ATTCAAGGCTGTGGGAAATG	CTCAGAGTACGCCAGGGAAC
mCcl2	AGGTCCCTGTCATGCTTCTG	GCTGCTGGTGATCCTCTTGT
mCcl3	TTCTCTGTACCATGACACTCTGC	CGTGGAATCTTCCGGCTGTAG
mCol1a1	CCTGGCAAAGACGGACTCAAC	GCTGAAGTCATAACCGCCACTG
mCol1a2	GTAACTTCGTGCCTAGCAACA	CCTTTGTCAGAATACTGAGCAGC
mCol3a1	CTGTAACATGGAAACTGGGGAAA	CCATAGCTGAACTGAAAACCACC
mVim	TTTCTCTGCCTCTGCCAAC	TCTCATTGATCACCTGTCCATC
mDes	CTAAAGGATGAGATGGCCCG	GAAGGTCTGGATAGGAAGGTTG
mActa2	GGCTCTGGGCTCTGTAAGG	CTCTTGCTCTGGGCTTCATC
mTgfb1	TGACGTCACTGGAGTTGTACGG	GGTTCATGTCATGGATGGTGC
mIgf1	GTGTGGACCGAGGGGCTTTTACTTC	GCTTCAGTGGGGCACAGTACATCTC
mIgf1r	GTGGGGGCTCGTGTTTCTC	GATCACCGTGCAGTTTTCCA

ESM Table 2. miRNA primer sequences used for qPCR

miRNA	Sequence (5' - 3')
mmu-miR-33-5p	GCGCATTGTAGTTGCATTGCA
mmu-miR-132-3p	TAACAGTCTACAGCCATGGTCG
mmu-miR-212-3p	TAACAGTCTCCAGTCACGGCCA
primer A1S	AATGATACGGCGACCACCGAGAT

ESM Table 3. Antibodies used for Western blotting, Immunoprecipitation and Chromatin immunoprecipitation

ANTIBODY	SOURCE	IDENTIFIER	Dilution
TCF4/TCF7L2 (C48H11)	Cell signaling Technology	Cat# 2569	1:1000
HSP90α/β (H-114)	Santa Cruz	Cat# sc-7947	1:5000
Akt	Cell signaling technology	Cat# 9272	1:1000
Phospho-Akt (Ser473)	Cell signaling technology	Cat# 9271	1:1000
GSK3β (27c10)	Cell signaling technology	Cat# 9315	1:1000
Phospho-GSK3β (Ser9)	Cell signaling technology	Cat# 9336	1:1000
ACC (C83B10)	Cell signaling technology	Cat# 3676	1:3000
FAS (C20G5)	Cell signaling technology	Cat#3180	1:3000
SREBP1	BD Biosciences	Cat#557036	1:1000
ChREBP	NOVUS	Cat# NB400-135	1:1000
PKLR (E-2) (referred to as 'L-PK' in Figure)	Santa Cruz	Cat# sc-133222	1:1000
Monoclonal ANTI-FLAG M2 (referred to as 'FLAG' in Figure)	Sigma Aldrich	Cat# F3165	1:3000
HA-probe	Santa Cruz	Cat# Sc-7392	1:3000
Mono-and polyubiquitinylated conjugates monoclonal antibody (FK2)	Enzo Life Sciences	Cat# BML-PW0150-0100	1:3000
AMPK	Cell signaling technology	Cat# 2532	1:1000
Phospho-AMPK (Thr172)	Cell signaling technology	Cat# 2535	1:1000
F4/80	Santa Cruz	Cat# sc-26643-R	1:1000
αSMA	abcam	Cat# ab5694	1:1000
m-IgGκ BP-HRP	Santa Cruz	Cat# sc-516102	1:3000
mouse anti-rabbit IgG-HRP	Santa Cruz	Cat# sc-2357,	1:3000

ESM Table 4. Antibodies used for Immunofluorescence staining

ANTIBODY	SOURCE	IDENTIFIER	Dilution
Insulin Monoclonal Antibody (ICBTACLS), Alexa Fluor 488	Thermo Fisher Scientific	Cat# 53-9769-82	1:500
Anti-Glucagon Antibody	Abcam	Cat# ab92517	1:500
Alexa Flour 594 chicken anti-rabbit IgG	Invitrogen	Cat# A21442	1:500

ESM Table 5. Sequences for miRNA mimics

Gene	Sense (5’- 3’)	Antisense (5 ’- 3’)
mmu-miR-33-5p	GUGCAUUGUAGUUGCAUUGCA	CAAUGCAACUACAAUGCACUU
mmu-miR-132-3p	UAACAGUCUACAGCCAUGGUCG	ACCAUGGCUGUAGACUGUUAUU
mmu-miR-212-3p	UAACAGUCUCCAGUCACGGCC	CCGUGACUGGAGACUGUUAUU
NC mimic	UUCUCCGAACGUGUCACGUTT	ACGUGACACGUUCGGAGAATT

ESM Table 6. Enriched reactome gene set in the liver of *Alb-Cre;TCF7L2*$^{f/f}$ mice under refeeding conditions

REACTOME	Gene set	Size	Overlap	*p*-value	FDR q-value
mmu556833	Metabolism of lipids	743	37	1.57E-14	2.54E-11
mmu1474244	Extracellular matrix organization	301	19	9.39E-10	7.58E-07
mmu163765	ChREBP activates metabolic gene expression	8	5	7.61E-09	4.10E-06
mmu8957322	Metabolism of steroids	153	13	1.30E-08	5.25E-06
mmu9006934	Signaling by Receptor Tyrosine Kinases	519	22	6.60E-08	2.13E-05
mmu73857	RNA Polymerase II Transcription	1375	38	1.38E-07	3.17E-05
mmu1655829	Activation of gene expression by SREBF (SREBP)	42	7	3.06E-07	7.07E-05
mmu9012999	RHO GTPase cycle	450	19	5.52E-07	8.95E-05
mmu211897	Cytochrome P450 - arranged by substrate type	66	8	5.53E-07	8.95E-05
mmu8978868	Fatty acid metabolism	177	12	5.54E-07	8.95E-05

ESM Table 7. Gene symbols of ChREBP and SREBP1c target genes from RNA-sequencing data

Gene symbol	Description
G0s2	G0/G1 Switch 2
Pklr	pyruvate kinase liver and red blood cell
Acaca	acetyl-CoA carboxylase
G6pc	glucose-6-phosphatase, catalytic
Klf10	Kruppel Like Factor 10
Adipor2	Adiponectin Receptor 2
Hif1a	Hypoxia Inducible Factor 1 Subunit Alpha
Fasn	fatty acid synthase
Acly	ATP citrate lyase
Thrsp	thyroid hormone responsive spot 14
Scd1	stearoyl-CoA desaturase-1
Elovl6	elongation of very long chain fatty acids protein 6
Slc2a2	solute carrier family 2 (facilitated glucose transporter), member 2
Srebf1	sterol regulatory element-binding transcription factor 1
Gpam	glycerol-3-phosphate acyltransferase 1
Rbl2	RB Transcriptional Corepressor Like 2
Gck	glucokinase
Pdk1	pyruvate dehydrogenase kinase, isoenzyme 1

ESM Table 8. Gene symbols of SREBP2 target genes from RNA-sequencing data

Gene symbol	Description
Srebf2	sterol regulatory element-binding transcription factor 2
Hmgcs2	3-Hydroxy-3-Methylglutaryl-CoA Synthase 2
Hmgcr	3-Hydroxy-3-Methylglutaryl-CoA Reductase
Ldlr	low density lipoprotein receptor
Fads1	Fatty acid desaturase 1
Fdft1	Farnesyl-Diphosphate Farnesyltransferase 1
Fdps	Farnesyl Diphosphate Synthase

ESM Figure & Figure legends

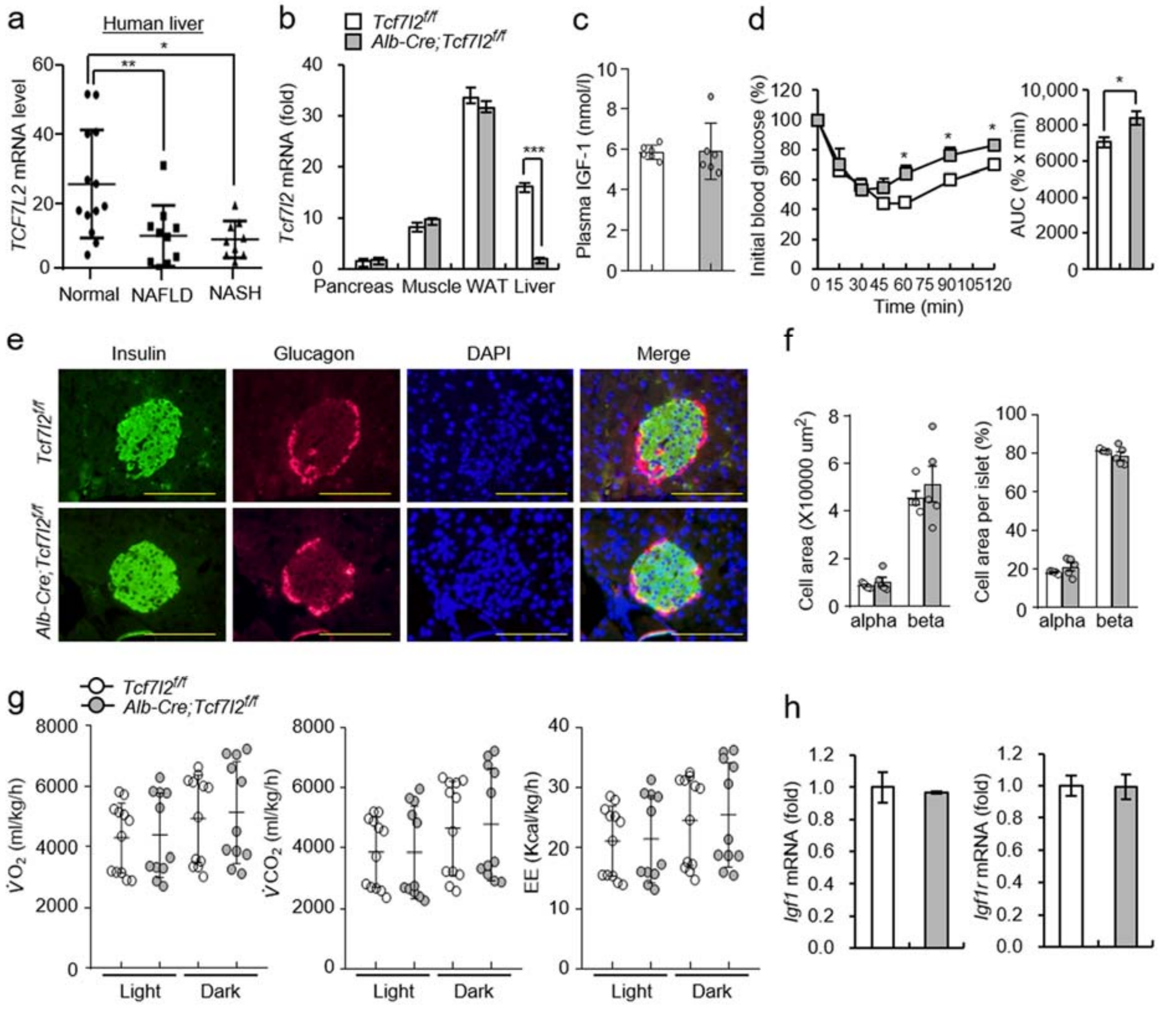

ESM Fig. 1 Metabolic phenotypes of *Alb-Cre;Tcf7l2*$^{f/f}$ mice on a normal chow diet (NCD). (**a**) Quantitative PCR (qPCR) analysis showing mRNA levels of *Tcf7l2* in the liver from healthy controls (*n*=13), NAFLD patients (*n*=10) and NASH patients (*n*=9). **p*<0.05, NAFLD patients vs healthy controls and **p*<0.05, NASH patients vs healthy controls by one-way ANOVA with Tukey's post hoc test. (**b**) qPCR analysis showing mRNA levels of *Tcf7l2* in peripheral tissues of 10-week-old *Tcf7l2*$^{f/f}$ (*n*=3) and *Alb-Cre;Tcf7l2*$^{f/f}$ (*n*=3) mice. (**c**) Plasma IGF-1 levels of 10-week-old *Tcf7l2*$^{f/f}$ (*n*=6) and *Alb-Cre;Tcf7l2*$^{f/f}$ (*n*=5) mice fasted for 6 h. (**d**) 11-week-old *Tcf7l2*$^{f/f}$ (*n*=9) and *Alb-Cre;Tcf7l2*$^{f/f}$ (*n*=6) mice fed an NCD. Insulin tolerance test (ITT, 1 U/kg body weight) and glucose area-under-the curve (AUC). Basal blood glucose level was defined as 100%. (**e**, **f**) 10-week-old *Tcf7l2*$^{f/f}$ (*n*=6) and *Alb-Cre; Tcf7l2*$^{f/f}$ (*n*=6) mice were fed an NCD. Representative images of pancreatic islets stained with insulin (green) and glucagon (red). Nuclei were stained with DAPI (blue). Scale bars = 100 μm (**e**). Quantification of the percentages of insulin-positive (beta cell) or glucagon-positive (alpha cell) area per islet (**f**). (**g**) $\dot{V}O_2$, $\dot{V}CO_2$ and energy expenditure (EE) in 10-week-old *Tcf7l2*$^{f/f}$ (*n*=12) and *Alb-Cre;Tcf7l2*$^{f/f}$ (*n*=11) mice fed an NCD. (**h**) qPCR analysis showing mRNA levels of *Igf1* and *Igf1r* in the livers of 10-week-old *Tcf7l2*$^{f/f}$ (*n*=6) and

Alb-Cre;Tcf7l2$^{f/f}$ (*n*=5) mice fasted for 6 h. Data in (**a**), (**c**), (**d**), (**f**) and (**g**) are presented as mean±SEM; data in (**b**) and (**h**) are presented as mean±SD; *p<0.05, **p<0.01, ***p<0.001, analysed by *t* test

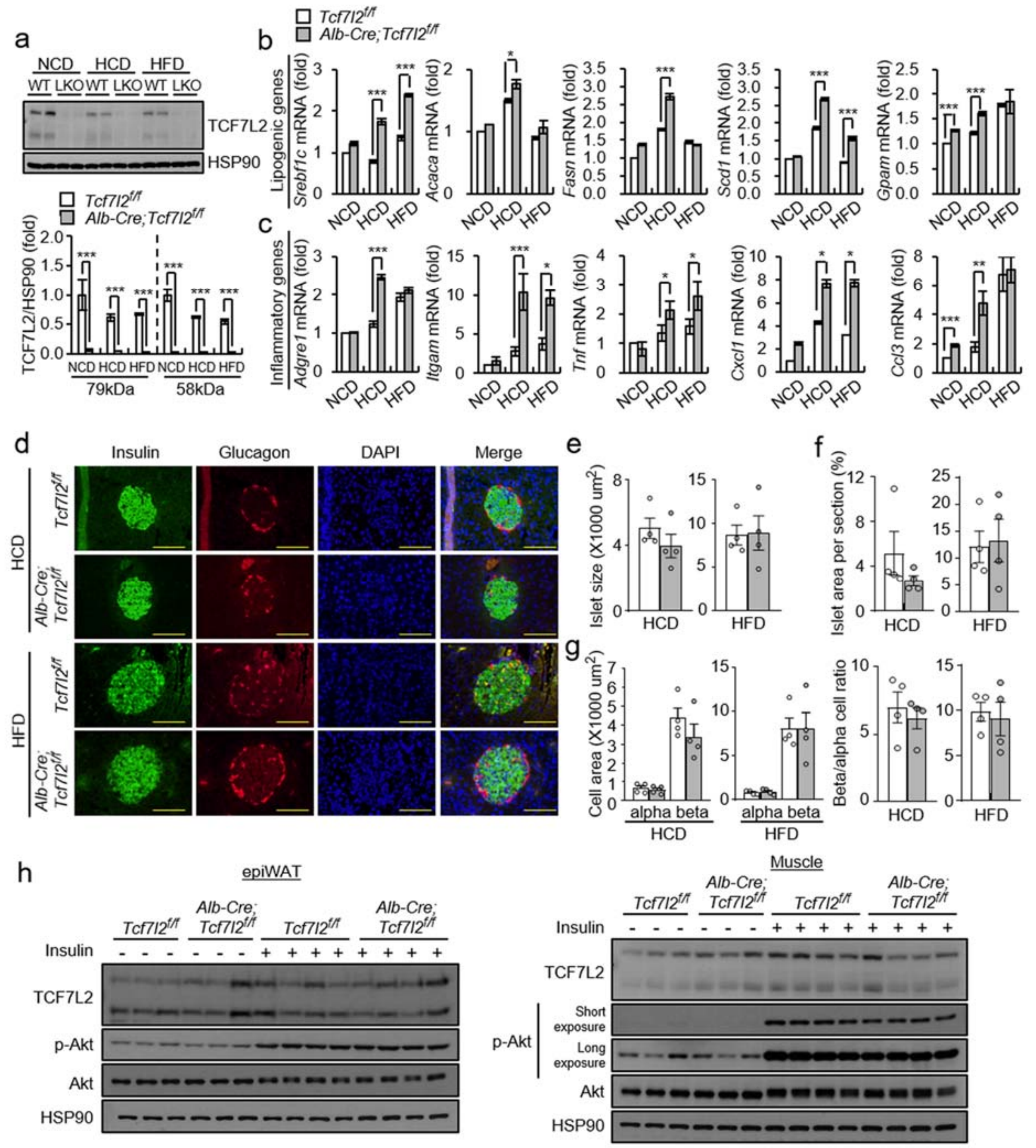

ESM Fig. 2 Metabolic phenotypes of *Alb-Cre;Tcf7l2^f/f^* mice fed an HCD or HFD for 22 weeks as diet-induced NAFLD/NASH models. (**a**) Representative western blot showing TCF7L2 expression in the liver of *Tcf7l2^f/f^* and *Alb-Cre;Tcf7l2^f/f^* mice fed an NCD, HCD or HFD for 22 weeks. The TCF7L2/HSP90 ratio is presented. (**b**, **c**) 6-week-old *Tcf7l2^f/f^* (*n*=4) and *Alb-Cre;Tcf7l2^f/f^* (*n*=4) mice were fed an NCD for 22 weeks. 6-week-old *Tcf7l2^f/f^* (*n*=7) and *Alb-Cre;Tcf7l2^f/f^* (*n*=7) mice were fed an HCD for 22 weeks. 6-week-old *Tcf7l2^f/f^* (*n*=7) and *Alb-Cre;Tcf7l2^f/f^* (*n*=5) mice were fed an HFD for 22 weeks. qPCR analysis showing hepatic expression of genes involved in lipogenesis (**b**) and inflammation (**c**). (**d**–**g**) 6-week-old *Tcf7l2^f/f^* (*n*=4) and *Alb-Cre; Tcf7l2^f/f^* (*n*=4) mice were given an HCD for 22 weeks. 6-week-old *Tcf7l2^f/f^* (*n*=4) and *Alb-Cre;Tcf7l2^f/f^* (*n*=4) mice were given an HFD for 22 weeks. Representative images of pancreatic islets stained with insulin (green) and glucagon (red). Nuclei were stained with DAPI (blue). Scale bars = 100 μm (**d**). Quantification of islet size (**e**). The proportion of islet

area per section (**f**). Quantification of insulin-positive (beta cell) or glucagon-positive (alpha cell) area per islet and the ratio of beta/alpha cell areas (**g**). (**h**) Representative western blot showing insulin signalling pathway in epididymal white adipose tissues (epiWAT) and skeletal muscle. 6-week-old *Tcf7l2*$^{f/f}$ (*n*=8) and *Alb-Cre;Tcf7l2*$^{f/f}$ (*n*=9) mice were fed an HCD for 22 weeks. Mice were fasted for 6 h, then injected intraperitoneally with insulin (10 U/kg body weight) or saline for 10 min. Data in (**a**–**c**) are presented as mean±SD; data in (**e**–**g**) are presented as mean±SEM; *p<0.05, **p<0.01, ***p<0.001, analysed by *t* test

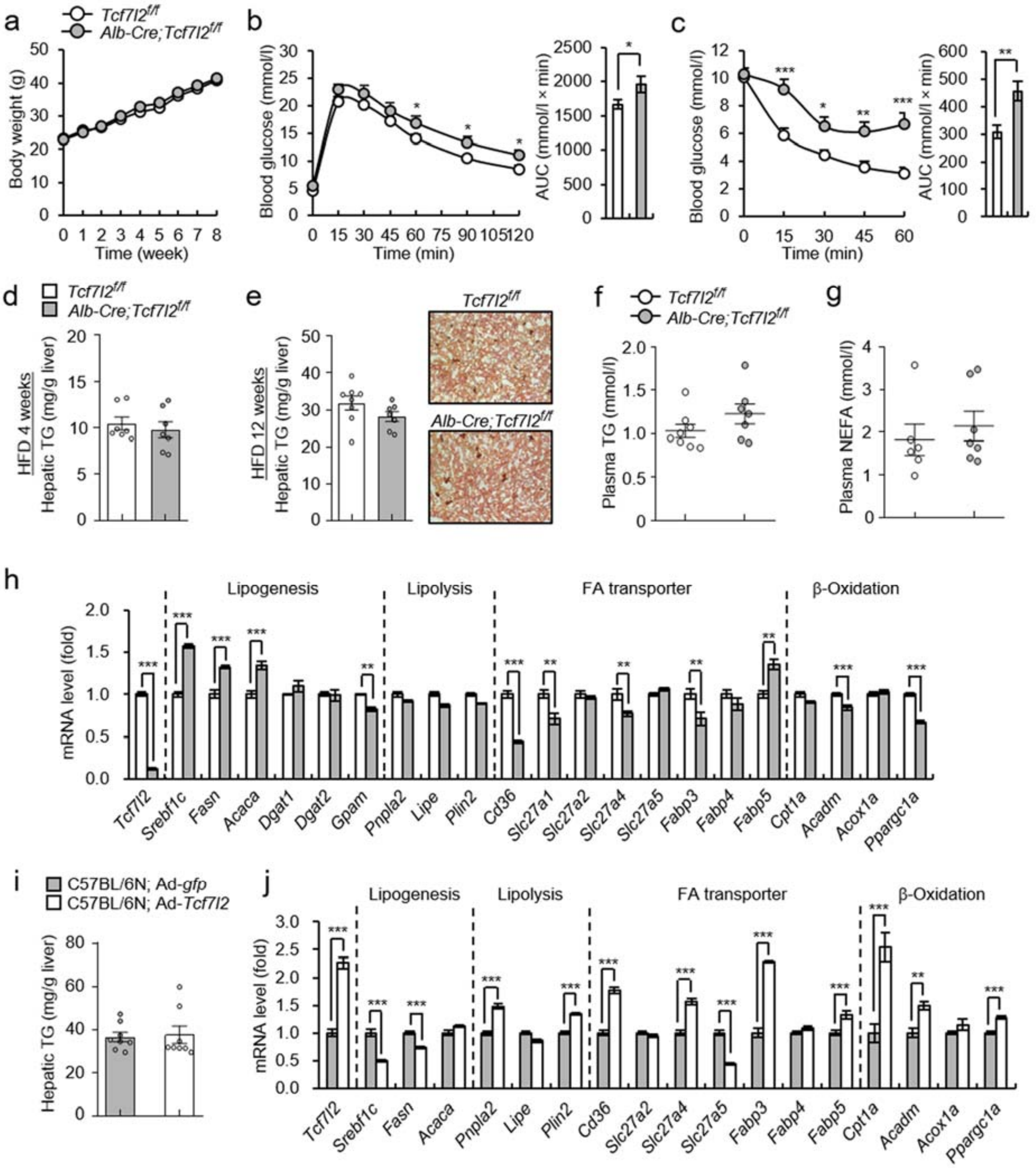

ESM Fig. 3 Effects of hepatic *Tcf7l2* on hepatic lipid metabolism under HFD conditions. (**a**–**c**) *Tcf7l2$^{f/f}$* (*n*=15) and *Alb-Cre;Tcf7l2$^{f/f}$* (*n*=14) mice were fed an HFD for 12 weeks. (**a**) Body weight changes during periods of HFD feeding. (**b**) Glucose tolerance test (GTT, 1.5 g/kg body weight) and glucose AUC. (**c**) ITT (1 U/kg body weight) and glucose AUC. (**d**) Hepatic triglyceride (TG) levels in *Tcf7l2$^{f/f}$* (*n*=7) and *Alb-Cre;Tcf7l2$^{f/f}$* (*n*=7) mice fed an HFD for 4 weeks. (**e**–**g**) *Tcf7l2$^{f/f}$* (*n*=8) and *Alb-Cre;Tcf7l2$^{f/f}$* (*n*=7) mice were fed an HFD for 12 weeks. Hepatic TG levels and Oil Red O staining of frozen liver sections in mice (**e**). Plasma TG levels (**f**). Plasma NEFA levels (**g**). (**h**) *Tcf7l2$^{f/f}$* (*n*=5) and *Alb-Cre;Tcf7l2$^{f/f}$* mice (*n*=7) were fed an HFD for 12 weeks. qPCR analysis showing hepatic expression levels of genes involved in lipogenesis, lipolysis, fatty acid transporter and β-oxidation in mice. (**i, j**) 6-week-old mice were fed an HFD for 12 weeks and were infected with Ad-*gfp* (*n*=8) or Ad-*Tcf7l2* (*n*=8) for 5 days. Hepatic TG levels in mice (**i**). qPCR analysis showing hepatic expression levels of

genes involved in lipogenesis, lipolysis, fatty acid transporter and β-oxidation in mice (**j**). Data in (**a**–**g**) and (**i**) are presented as mean±SEM and data in (**h**) and (**j**) are presented as mean±SD; *p<0.05, **p<0.01, ***p<0.001, analysed by *t* test

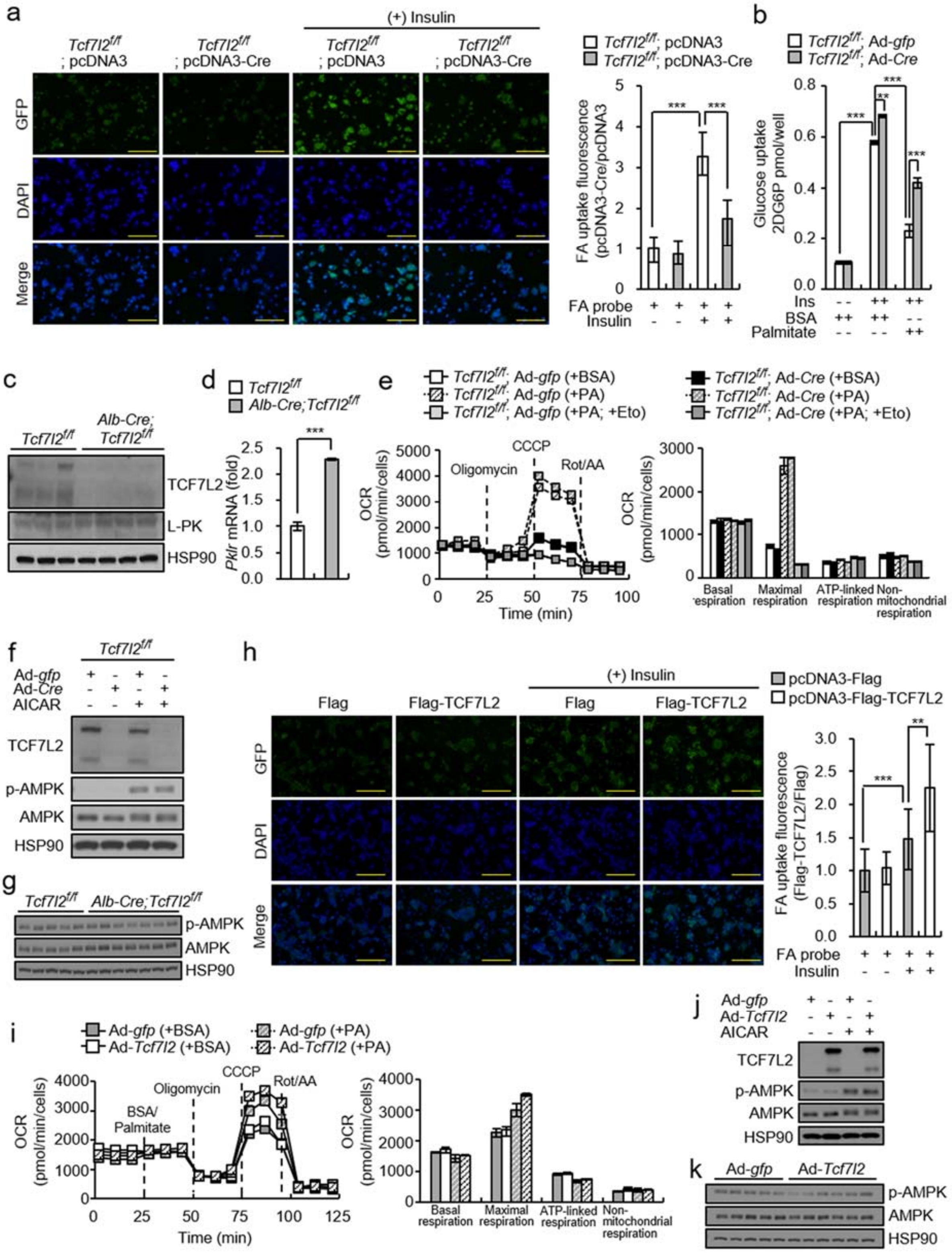

ESM Fig. 4 Effects of hepatic *Tcf7l2* on fatty acid uptake and β-oxidation. (**a**) Fluorescence images showing fatty acid uptake (green) into primary hepatocytes isolated from *Tcf7l2*$^{f/f}$ mice. Mouse primary hepatocytes were transfected with pcDNA3 or pcDNA3-Cre and treated with 10 nmol/l insulin for 30 min. Nuclei were labelled with DAPI (blue). Quantification of fluorescent fatty acid uptake. Scale bars = 100 μm. ****p*<0.001, assessed by one-way ANOVA with Tukey's post hoc test. (**b**) Measurement of 2-deoxyglucose (2-DG) uptake in primary hepatocytes derived from

Tcf7l2$^{f/f}$ mice. Mouse primary hepatocytes were infected with Ad-*gfp* or Ad-*Cre* and treated 1 nmol/l Insulin, BSA or 300 μmol/l palmitate for 6 h. ***p*<0.01, ****p*<0.001, assessed by one-way ANOVA with Tukey's post hoc test. (**c**, **d**) *Tcf7l2*$^{f/f}$ (*n* = 5) and *Alb-Cre;Tcf7l2*$^{f/f}$ mice (*n*=7) were given an HFD for 12 weeks. Representative western blot showing protein levels of TCF7L2 and L-PK in mouse livers (**c**). qPCR analysis showing mRNA levels of *Pklr* in mouse livers (**d**). (**e**) Measurement of fatty acid oxidation using oxygen consumption rate (OCR). Mouse primary hepatocytes derived from *Tcf7l2*$^{f/f}$ mice were infected with Ad-*gfp* or Ad-*Cre*, incubated with BSA or PA, and treated with etomoxir (Eto), oligomycin, FCCP, rotenone and antimycin A. (**f**, **g**) Representative western blot showing effects of *Tcf7l2* knockout on p-AMPK and AMPK protein levels. Primary hepatocytes isolated from *Tcf7l2*$^{f/f}$ mice were infected with Ad-*gfp* or Ad-*Cre* and treated with 300 μmol/l AICAR for 1 h (**f**). *Tcf7l2*$^{f/f}$ (*n*=5) and *Alb-Cre;Tcf7l2*$^{f/f}$ mice (*n*=7) were fed an HFD for 12 weeks (**g**). (**h**) Fluorescence images showing fatty acid uptake (probe: green) into mouse primary hepatocytes transfected pcDNA-Flag or pcDNA-Flag-TCF7L2 and treated with 10 nmol/l insulin for 30 min. Nuclei were labelled with DAPI (blue). Quantification of fatty acid uptake fluorescence. Scale bars = 100 μm. ***p*<0.01, ****p*<0.001, assessed by one-way ANOVA with Tukey's post hoc test. (**i**) Measurement of fatty acid oxidation using OCR. Mouse primary hepatocytes isolated from 10-week-old mice were infected with Ad-*gfp* or Ad-*Cre*, incubated with BSA or PA and treated with oligomycin, FCCP and rotenone. (**j**) Representative western blot showing protein levels of TCF7L2, p-AMPK and AMPK in mouse primary hepatocytes infected with Ad-*gfp* or Ad-*Tcf7l2* and treated with 300 μmol/l AICAR for 1 h. (**k**) Representative western blot showing protein levels of p-AMPK and AMPK in the liver of mice given an HFD for 12 weeks and infected with Ad-*gfp* or Ad-*Tcf7l2* for 5 days. Data in (**a**), (**b**) and (**h**) are presented as mean±SEM; Data in (**d**), (**e**) and (**i**) are presented as mean±SD; ***p*<0.01, ****p*<0.001, analysed by *t* test

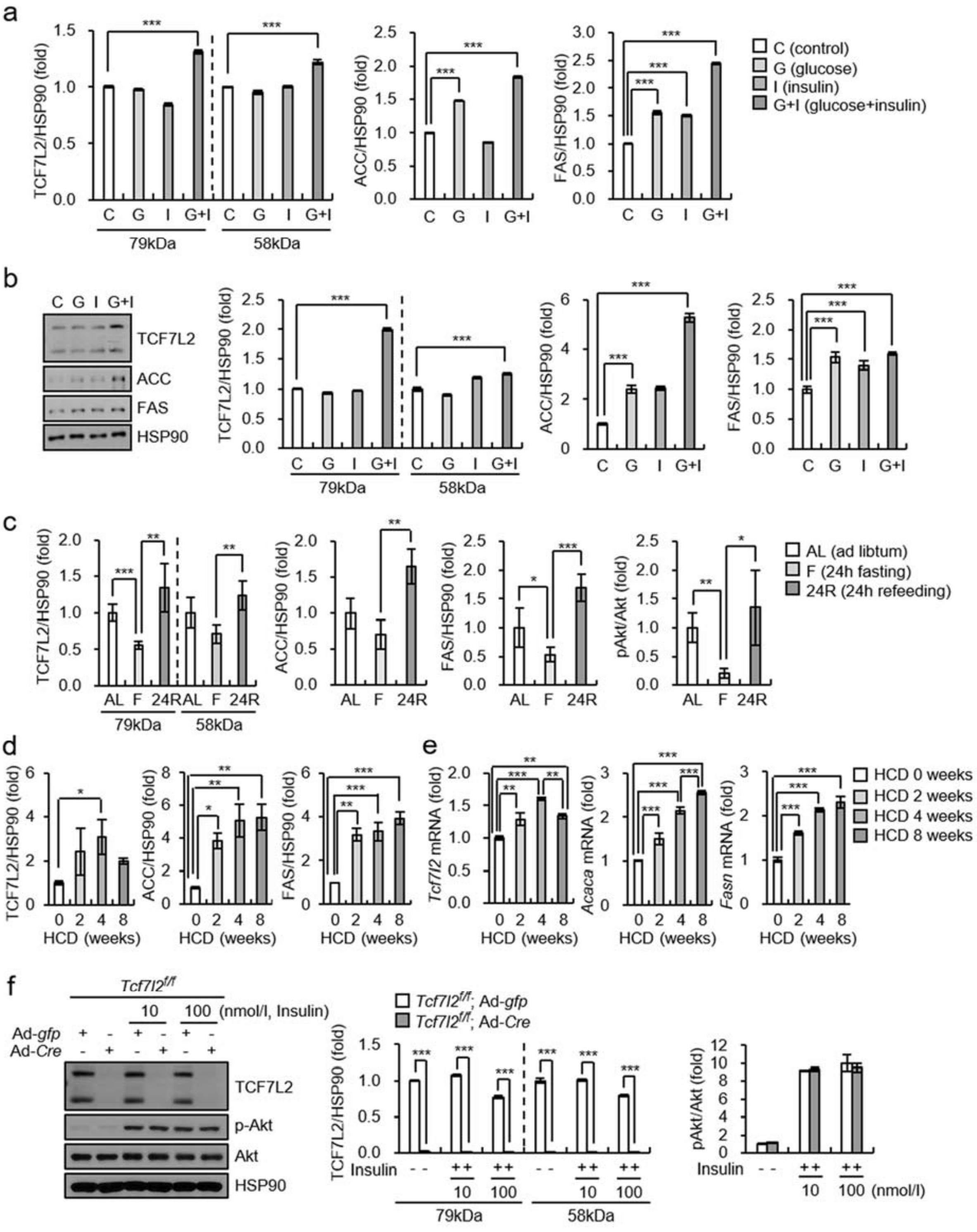

ESM Fig. 5 Expression pattern of hepatic *Tcf7l2* following glucose/insulin treatment and HCD feeding and effects of hepatic *Tcf7l2* knockout on insulin signalling. (**a**) Primary hepatocytes isolated from C57BL/6N mice were treated with 25 mmol/l glucose (G) and/or 10 nmol/l insulin (I) or saline (C: control) for 48 h. The quantified relative protein levels of representative western blots in Fig. 3b: TCF7L2/HSP90, ACC/HSP90 and FAS/HSP90 ratios. ***$p<0.001$, assessed by one-way ANOVA with Tukey's post hoc test. (**b**) Primary hepatocytes isolated from C57BL/6N mice were treated with 25 mmol/l glucose (G) and/or 100 nmol/l insulin (I) or saline (C: control) for 48 h. Representative western blot showing protein levels of TCF7L2, ACC and FAS

is shown alongside the quantified relative protein levels: TCF7L2/HSP90, ACC/HSP90 and FAS/HSP90 ratios. ***p<0.001, assessed by one-way ANOVA with Tukey's post hoc test. (**c**) The quantified relative protein levels of representative western blots in Fig. 3b showing TCF7L2, ACC, FAS, p-Akt and Akt levels in the liver of 10-week old C57BL/6N mice under ad libitum (AL; n=4), 24 h fasted (F; n=4) or 24 h refed (24R; n=4) conditions: TCF7L2/HSP90, ACC/HSP90, FAS/HSP90 and p-AKT/AKT ratios. *p<0.05, **p<0.01, ***p<0.001, assessed by one-way ANOVA with Tukey's post hoc test. (**d, e**) C57BL/6N mice were fed an NCD or HCD for 2, 4 and 8 weeks (n=5 per group). The quantified relative protein levels of representative western blots in Fig. 3c showing protein levels of TCF7L2, ACC and FAS: TCF7L2/HSP90, ACC/HSP90 and FAS/HSP90 ratios (**d**). qPCR analysis showing mRNA levels of *Tcf7l2*, *Acaca* and *Fasn* in mouse livers (**e**). *p<0.05, **p<0.01, ***p<0.001, assessed by one-way ANOVA with Tukey's post hoc test. (**f**) Primary hepatocytes from *Tcf7l2*$^{f/f}$ mice were infected with Ad-*gfp* or Ad-*Cre* and treated with 10 nmol/l or 100 nmol/l insulin for 10 min. Representative western blot showing cell-autonomous effects of *Tcf7l2* depletion on insulin signalling pathway is shown alongside the quantified relative protein levels: TCF7L2/HSP90 and p-Akt/Akt ratios. Data in (**a**–**e**) are presented as mean±SEM; Data in (**f**) are presented as mean±SD; ***p<0.001, analysed by t test

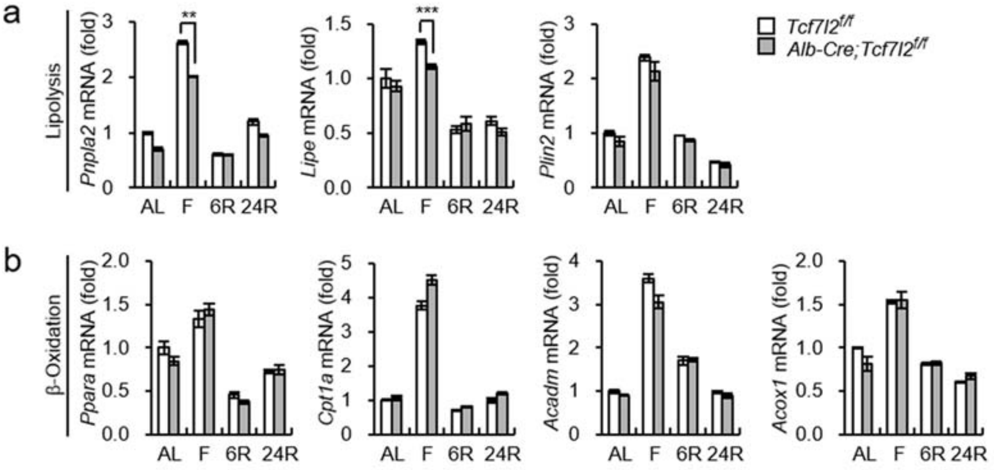

ESM Fig. 6 Effects of hepatic *Tcf7l2* deficiency on levels of lipid metabolism-related genes. 10-week-old *Tcf7l2*$^{f/f}$ (*n*=5) and *Alb-Cre;Tcf7l2*$^{f/f}$ (*n*=7) mice were fed regular chow ad libitum (AL). 10-week-old *Tcf7l2*$^{f/f}$ (*n*=6) and *Alb-Cre;Tcf7l2*$^{f/f}$ (*n*=7) mice were fasted for 24 h (F). 10-week-old *Tcf7l2*$^{f/f}$ (*n*=6 per group) and *Alb-Cre;Tcf7l2*$^{f/f}$ (*n*=7 per group) mice were fasted for 24 h (F) and refed for either 6 h (6R) or 24 h (24R). (**a**, **b**) qPCR analysis showing expression levels of genes involved in lipolysis (**a**) and β–oxidation (**b**) in mouse livers. Data in (**a**) and (**b**) are presented as mean±SD; ***p*<0.01, ****p*<0.001, analysed by *t* test

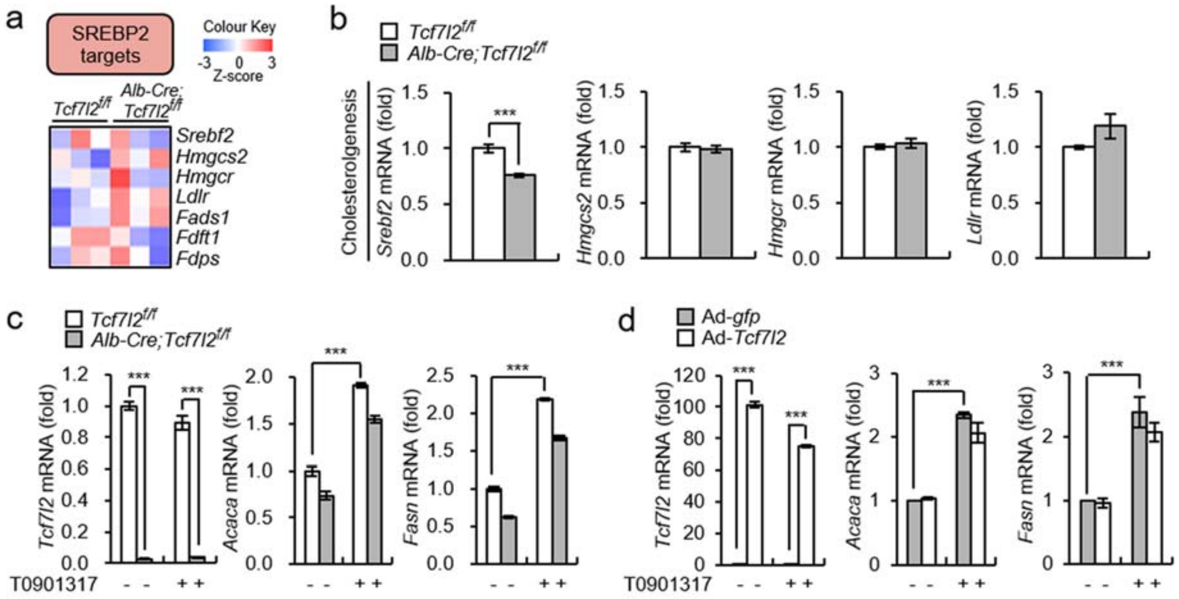

ESM Fig. 7 Effects of hepatic *Tcf7l2* on genes involved in cholesterolgenesis and LXRα-mediated lipogenesis. (**a**) Heatmaps visualising hepatic expression of SREBP2 target genes in *Tcf7l2*$^{f/f}$ and *Alb-Cre;Tcf7l2*$^{f/f}$ mice under refeeding conditions (See ESM Table 8 for gene symbols). (**b**) qPCR analysis showing the hepatic expression levels of genes involved in cholesterolgenesis in *Tcf7l2*$^{f/f}$ (*n*=6) and *Alb-Cre;Tcf7l2*$^{f/f}$ (*n*=7) mice under refeeding conditions. (**c**) qPCR analysis showing expression of *Tcf7l2*, *Acaca* and *Fasn* in primary hepatocytes of *Tcf7l2*$^{f/f}$ and *Alb-Cre;Tcf7l2*$^{f/f}$ mice. Cells were treated with 1 μmol/l T0901317 (LXR agonist) for 24 h. ***p<0.001, assessed by one-way ANOVA with Tukey's post hoc test. (**d**) qPCR analysis showing expression of *Tcf7l2*, *Acaca* and *Fasn* in mouse primary hepatocytes infected with Ad-*gfp* or Ad-*Tcf7l2* and treated with 1 μmol/l T0901317 for 24 h. ***p<0.001, assessed by one-way ANOVA with Tukey's post hoc test. Data in (**c**) and (**d**) are presented as mean±SEM; Data in (**b**) are presented as mean±SD; ***p<0.001, analysed by *t* test

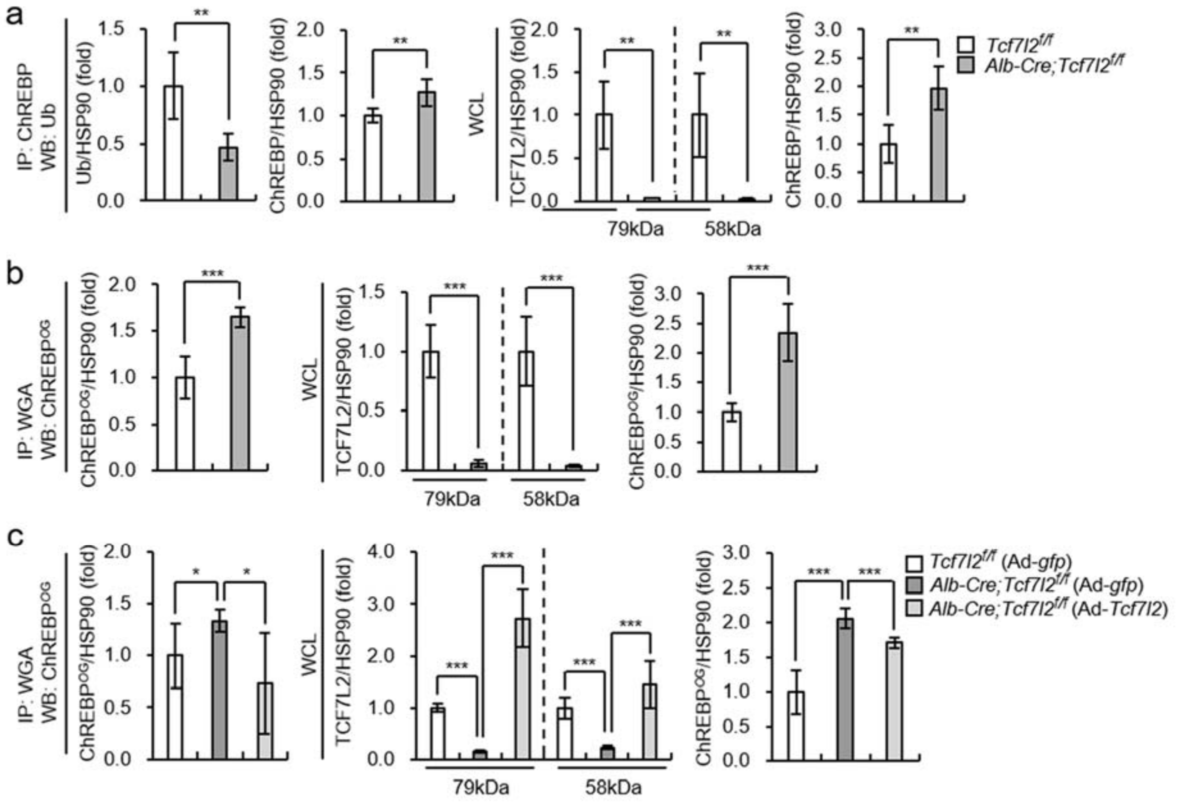

ESM Fig. 8 O-GlcNAcylation and protein stability of ChREBP in the livers of *Alb-Cre;Tcf7l2$^{f/f}$* mice. (**a**) *Tcf7l2$^{f/f}$* (*n*=3) and *Alb-Cre;Tcf7l2$^{f/f}$* (*n*=3) mice were subjected to HCR for 24 h. Liver protein samples were immunoprecipitated with anti-ChREBP. The quantified relative protein levels of representative western blot in Fig. 6j: ubiquitin (Ub)/HSP90 (IP), ChREBP/HSP90 (IP), TCF7L2/HSP90 (WCL) and ChREBP/HSP90 (WCL) ratios. IP, immunoprecipitation; WCL, whole-cell lysate. ***p*<0.01, assessed by one-way ANOVA with Tukey's post hoc test. (**b**) *Tcf7l2$^{f/f}$* (*n*=3) and *Alb-Cre;Tcf7l2$^{f/f}$* (*n*=3) mice were subjected to HCR for 24 h. Liver protein samples were immunoprecipitated with anti-WGA. The quantified relative protein levels of representative western blot in Fig. 6k: ChREBP/HSP90 (IP), TCF7L2/HSP90 (WCL) and ChREBP/HSP90 (WCL) ratios. IP, immunoprecipitation; WCL, whole-cell lysate. ****p*<0.001, assessed by one-way ANOVA with Tukey's post hoc test. (**c**) *Tcf7l2$^{f/f}$* and *Alb-Cre;Tcf7l2$^{f/f}$* mice were infected with Ad-*gfp* or adenovirus expressing *Tcf7l2* (Ad-*Tcf7l2*) via the tail vein and then subjected to HCR for 24 h (*n*=3 per group). Liver protein samples were immunoprecipitated with WGA. The quantified relative protein levels of representative western blot in Fig. 6l: ChREBP/HSP90 (IP), TCF7L2/HSP90 (WCL) and ChREBP/HSP90 (WCL) ratios. IP, immunoprecipitation; WCL, whole-cell lysate. **p*<0.05, ****p*<0.001, assessed by one-way ANOVA with Tukey's post hoc test. Data in (**a**–**c**) are presented as mean±SEM.

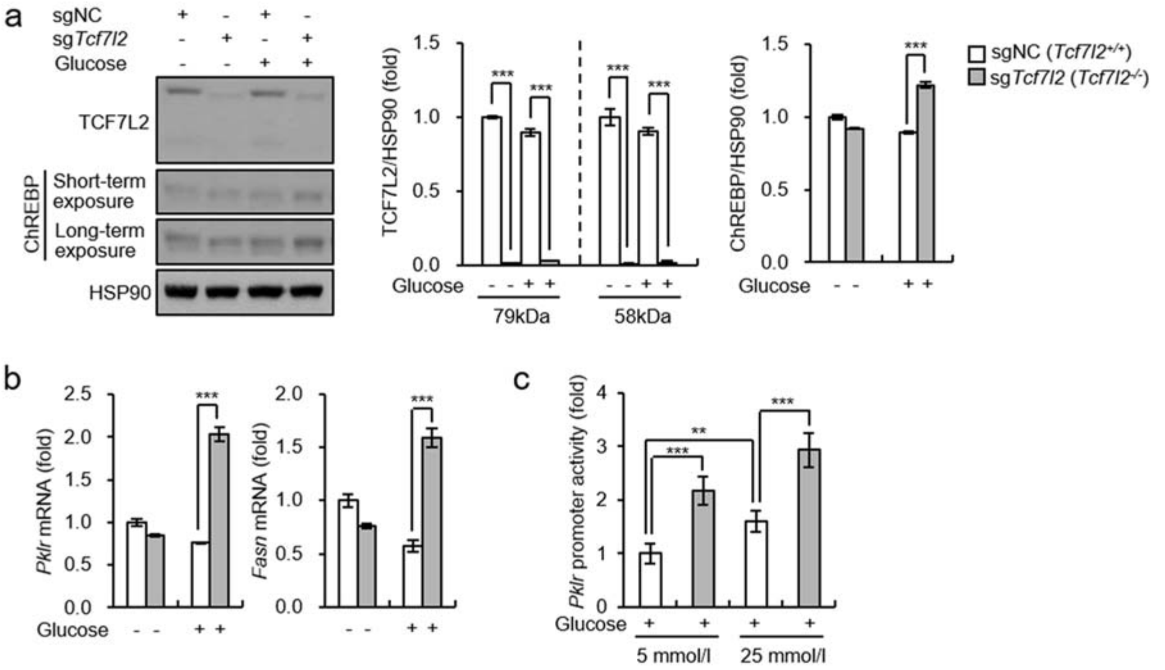

ESM Fig. 9 High glucose-induced levels of ChREBP protein and its target factors in *Tcf7l2* knockout AML12 cell lines. *Tcf7l2* WT (single-guide negative control [sgNC]; *Tcf7l2*$^{+/+}$) and *Tcf7l2* KO (sg*Tcf7l2*; *Tcf7l2*$^{-/-}$) AML12 cell lines were generated using the CRISPR-Cas9 system. (**a**) Representative western blot showing TCF7L2 and ChREBP protein levels in sgNC and sg*Tcf7l2* AML12 cells treated with 25 mmol/l glucose for 24 h. The TCF7L2/HSP90 and ChREBP/HSP90 ratio. (**b**) qPCR analysis showing expression of *Pklr* and *Fasn* in sgNC and sg*Tcf7l2* AML12 cells treated with 25 mmol/l glucose for 24 h. (**c**) Luciferase assay showing effects of *Tcf7l2* knockout on *Pklr* promoter activity under low glucose (5 mmol/l) or high-glucose (25 mmol/l) conditions. ***p*<0.01, ****p*<0.001, assessed by one-way ANOVA with Tukey's post hoc test. Data in (**a**) and (**b**) are presented as mean±SD; ****p*<0.001, analysed by *t* test. Data in (**c**) are presented as mean±SEM.

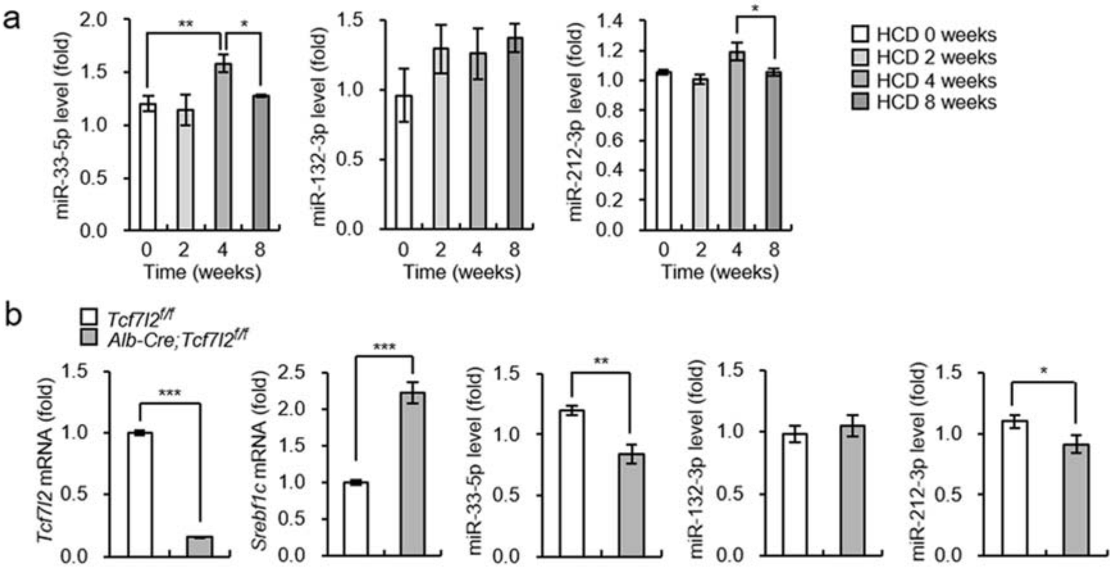

ESM Fig. 10 Expression patterns of *Tcf7l2*, *Srebf1c* and miRs during HCD feeding. (**a**) C57BL/6N mice were fed an NCD or HCD for 2, 4 and 8 weeks (n=5 per group). qPCR analysis showing expression of miR-33-5p, miR-132-3p and miR-212-3p in mouse livers. *p<0.05, **p<0.01, assessed by one-way ANOVA with Tukey's post hoc test. (**b**) 6-week-old *Tcf7l2*$^{f/f}$ (n=7) and *Alb-Cre;Tcf7l2*$^{f/f}$ (n=7) mice were fed an HCD for 22 weeks. qPCR analysis showing expression of *Tcf7l2*, *Srebf1c*, miR-33-5p, miR-132-3p and miR-212-3p in mouse livers. Data in (**a**) are presented as mean±SEM; Data in (**b**) are presented as mean±SD; *p<0.05, **p<0.01, ***p<0.001, analysed by t test

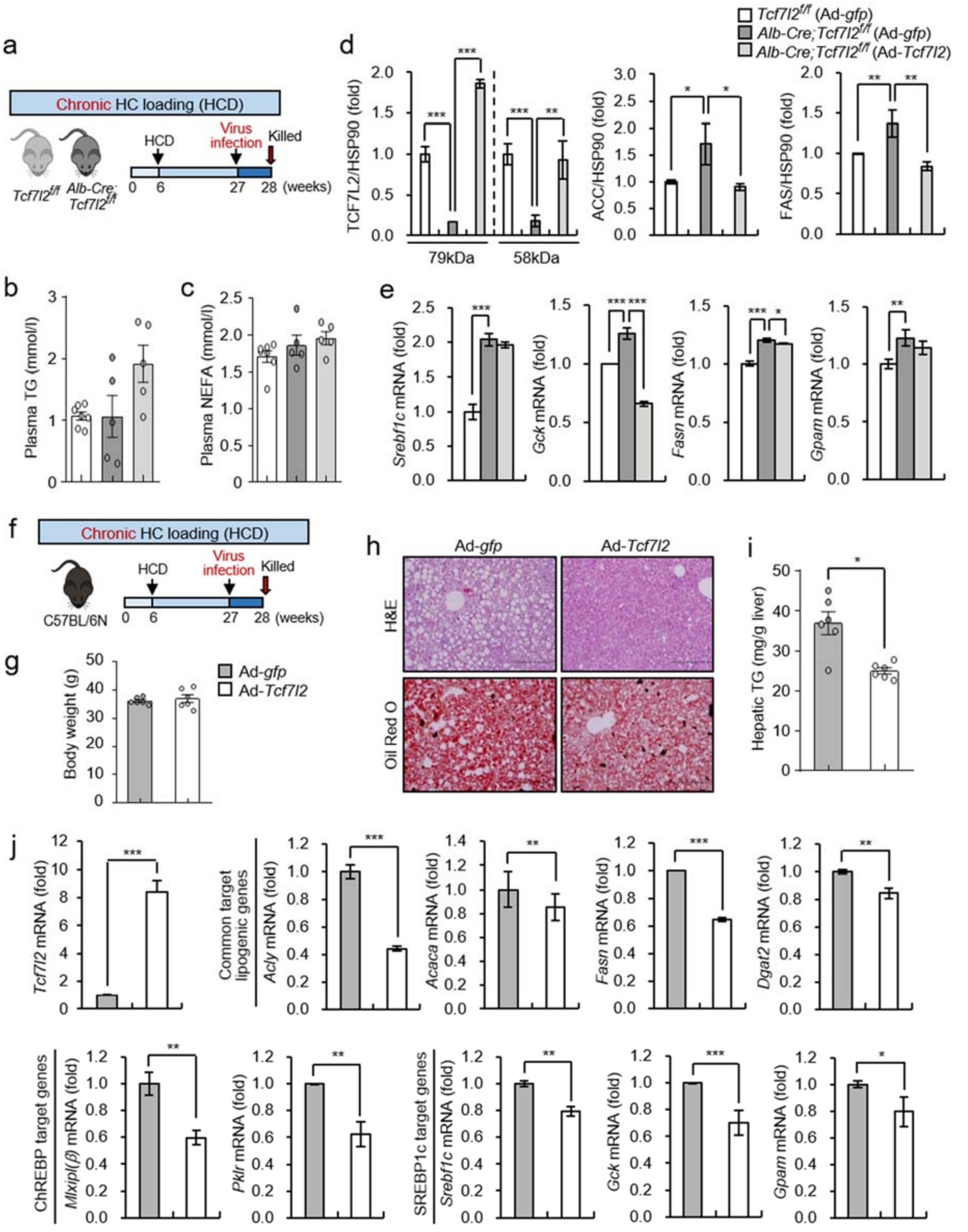

ESM Fig. 11 Hepatic *Tcf7l2* expression restores hepatic DNL gene expression increased by chronic HCD feeding. (**a**–**e**) 6-week-old *Tcf7l2*$^{f/f}$ and *Alb-Cre;Tcf7l2*$^{f/f}$ mice were fed an HCD for 22 weeks. At 21 weeks of HCD, *Tcf7l2*$^{f/f}$ mice were infected with Ad-*gfp* (*n*=7) and *Alb-Cre;Tcf7l2*$^{f/f}$ mice were infected with Ad-*gfp* (*n*=5) or Ad-*Tcf7l2* (*n*=5). A schematic diagram showing experimental design (**a**). Plasma TG levels (**b**). Plasma NEFA levels (**c**). The quantified relative protein levels of representative western blot in Fig. 8j: TCF7L2/HSP90, ACC/HSP90 and FAS/HSP90 ratios. *p<0.05, **p<0.01, ***p<0.001, assessed by one-way ANOVA with Tukey's post hoc test (**d**).

qPCR analysis showing mRNA levels of *Srebf1c*, *Gck*, *Fasn* and *Gpam* in mouse livers. *$p<0.05$, **$p<0.01$, ***$p<0.001$, assessed by one-way ANOVA with Tukey's post hoc test (**e**). (**f**–**j**) 6-week-old C57BL/6N mice were fed an HCD for 22 weeks. At 21 weeks of HCD, mice were infected with Ad-*gfp* (*n*=7) or Ad-*Tcf7l2* (*n*=8). A schematic diagram showing experimental design (**f**). Body weights (**g**). Representative H&E and Oil Red O staining of liver sections; (×20 magnification, Scale bars = 200 μm) (**h**). Hepatic TG levels (**i**). qPCR analysis showing effects of hepatic *Tcf7l2* expression on specific target genes and commonly shared lipogenic target genes of SREBP1c and ChREBP in the livers (**j**). Data in (**b**), (**c**), (**d**), (**e**), (**g**) and (**i**) are presented as mean±SEM; data in (**j**) are presented as mean±SD; *$p<0.05$, **$p<0.01$, ***$p<0.001$, analysed by *t* test

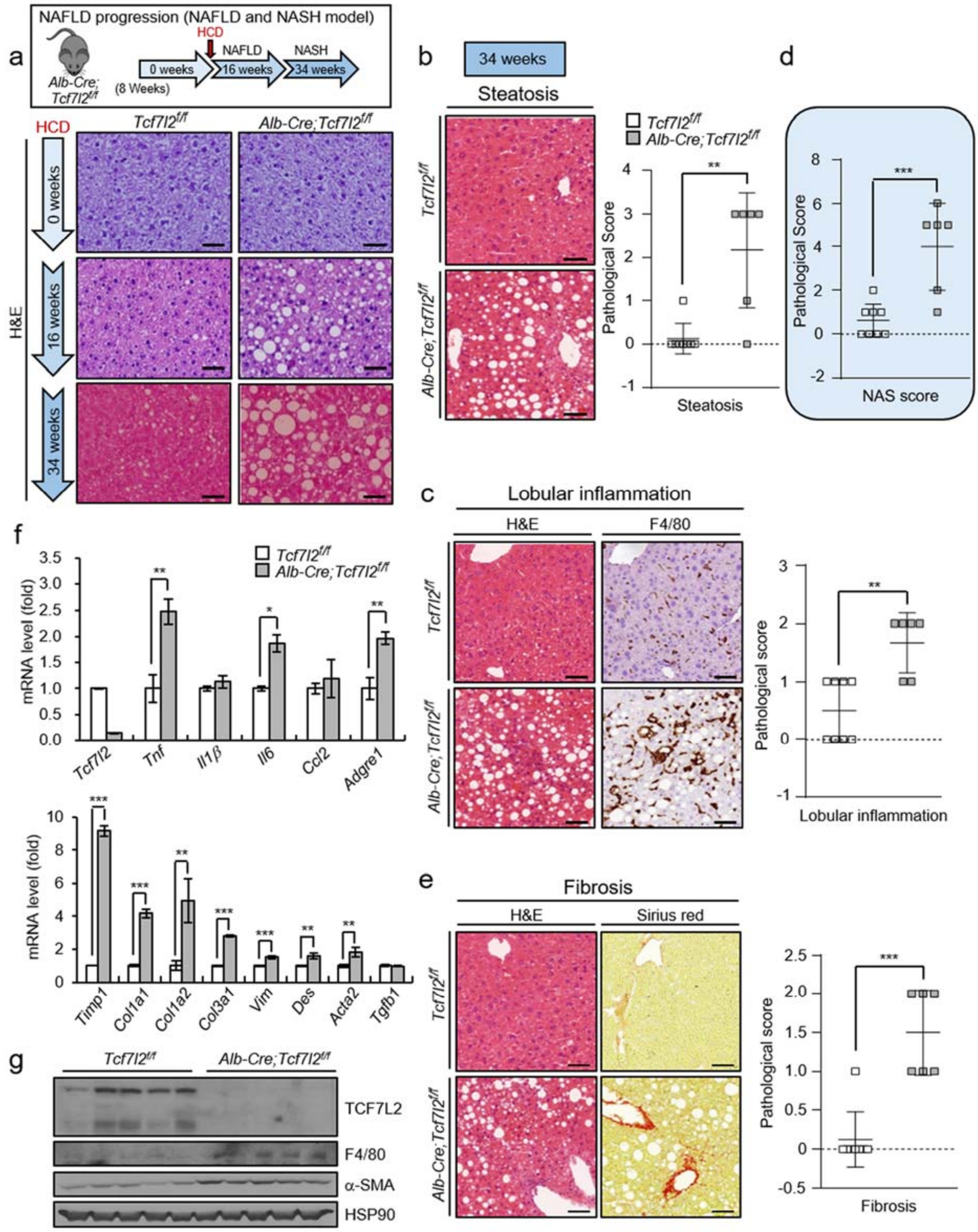

ESM Fig. 12 NASH phenotypes in *Alb-Cre;Tcf7l2^f/f^* mice fed an HCD. 8-week-old *Tcf7l2^f/f^* (*n*=11) and *Alb-Cre;Tcf7l2^f/f^* (*n*=8) mice were fed an HCD for 34 weeks. Among them, *Tcf7l2^f/f^* (*n*=2) and *Alb-Cre;Tcf7l2^f/f^* (*n*=2) mice were killed to observe liver histology at 16 weeks of HCD. The rest of *Tcf7l2^f/f^* (*n*=9) and *Alb-Cre;Tcf7l2^f/f^* (*n*=6) mice were killed at 34 weeks of HCD. (**a**) A schematic of experimental design for observing NAFLD progression. Representative images of livers stained with H&E; 20× magnification. (**b**–**e**) Histology and pathological score of steatosis (**b**), lobular inflammation (**c**), the NAFLD Activity Score (NAS) (**d**) that represents the sum of

scores for steatosis, lobular inflammation and ballooning, and fibrosis (**e**) in the liver sections from *Tcf7l2*$^{f/f}$ and *Alb-Cre;Tcf7l2*$^{f/f}$ mice fed an HCD for 34 weeks; 40× magnification. (**f**) qPCR analysis showing mRNA levels of inflammation and fibrosis markers in the liver of *Tcf7l2*$^{f/f}$ and *Alb-Cre;Tcf7l2*$^{f/f}$ mice. (**g**) Representative western blot showing protein levels of TCF7L2, inflammatory F4/80 and fibrotic α-SMA in the livers of mice fed an HCD for 34 weeks. Data in (**b**–**e**) are presented as mean±SEM and data in (**f**) are presented as mean±SD; *p<0.05, **p<0.01, ***p<0.001, analysed by *t* test
